# Supplementary material for: Predicting the presence of infectious virus from PCR data: A meta-analysis of SARS-CoV-2 in non-human primates
Source: PLoS Pathog. 2024 Apr 29;20(4):e1012171. doi: 10.1371/journal.ppat.1012171 (PMC11081500; doi:10.1371/journal.ppat.1012171)
Supplement: S1 Methods — (PDF) [file ppat.1012171.s001.pdf]

Supplementary Methods

Comprehensive literature search

To construct our database, we conducted comprehensive literature searches on 11 March 2021, which was the chosen cutoff date for inclusion in the database. One author (CS) screened the Web of Science (Core Collection) and PubMed for articles published in English with the following search string: (SARS-CoV-2 OR COVID-19) AND (primate* OR macaque* OR monkey* OR "macaca" OR "chlorocebus"). On the same day, CS also jointly screened bioRxiv and medRxiv, with two separate search strings: (i) (SARS-CoV-2 OR COVID-19) AND (primate* OR macaque* OR monkey*), and (ii) (SARS-CoV-2 OR COVID-19) AND ("macaca" OR "chlorocebus"). These searches returned 163 records from Web of Science, 761 from PubMed, and 259 from bioRxiv and medRxiv. An additional 60 records were obtained from Google Scholar searches and citation trackers (before 11 March 2021) using the following search terms: SARS-CoV-2, COVID-19, macaque, monkey, non-human primate (**S1 Fig**). In total, this returned 1,243 results, with 866 unique records after removing duplicates. Of those, the following were immediately excluded: (i) articles published before 2020, (ii) article types that do not generate primary data (e.g., opinions, reviews), and (iii) articles with clearly irrelevant titles based on our predefined eligibility/inclusion criteria (described in the **Methods**). CS inspected the abstracts of the remaining 275 studies and the full texts of 122 records, according to the same eligibility criteria.

Data collection process

For each included article and every infected individual, the following study design details were obtained from the text, figures, supplementary files, or the corresponding authors, when available: primate species, rhesus macaque origin (Chinese or Indian), ID, sex, age (years or months), age class, treatment group, viral inoculum strain, inoculation route(s), and route specific inoculation dose(s). For every sample, the following information was recorded when available: sample value (converted to log10, when quantitative and reported otherwise), sample time (day post infection, starting at 0), sample type (e.g., swab, tissue), sample location (e.g., liver), sample units (e.g., viral RNA copies/mL), method of quantification (e.g., RT-qPCR, plaque assay), target gene (for PCR), cell line (e.g., VeroE6, for viral culture), and limit of detection and/or quantification. We standardized all ID names as follows: [the first and last initial of the study's first author] _ [the ID name as assigned in the original study, if available].

When raw data was not published or methodological/biological details were missing or inconsistently reported, we contacted the corresponding author(s) using a standardized email template (paired with study-specific questions) to resolve discrepancies and/or request raw data. When clarification and/or raw data were obtained, the details were updated accordingly. In instances where clarification was not obtained but conflicting information existed in the article, we recorded the information most consistent with the methods section. Unclarified information that was missing entirely from the article materials is considered unknown.

Standardizing age class and inoculation doses

We developed a consistent method to assign age classes for all studies, following methods used in prior studies (1–5). When age was reported in months and years, juvenile rhesus and cynomolgus macaques include individuals with ages <5 years, adults include 5-19 years, and geriatrics include ≥20 years. Juvenile African green monkeys include ages <5 years, adults include 5-14 years, and geriatrics include ≥15 years. When reported age ranges spanned multiple assignments, we used the reported age class if available, otherwise we considered it unknown.

Since inoculation doses are frequently reported as either plaque forming units (pfu) or tissue culture infectious dose 50 (TCID50), we converted all inoculation doses reported in TCID50 to pfu using a standard conversion factor (1 TCID50=0.69 pfu) (6).

Confirming RNA types

To confirm whether articles quantified full-length genomic RNA, subgenomic RNA, or total RNA (since reporting practices varied across studies), all relevant primer and probe sequences were extracted from each study or from referenced articles. We used SnapGene software (from Insightful Science; available at snapgene.com) to map all sequences to the SARS-CoV-2/human/CHN/Wu-1 reference genome. Any assays with both forward and reverse primers located within the ORF1ab gene were considered to amplify full-length genomic RNA, as this gene is not located on any canonical subgenomic RNAs. Assays with both forward and reverse primers located within any individual gene downstream of ORF1ab were considered to amplify total RNA, as these sequences can be found on both subgenomic and full-length genomic RNA. Assays where the forward primer was located in the 5' UTR but the reverse primer was located within a gene downstream of ORF1ab were classified as amplifying subgenomic RNA.

Justification and prior description for candidate predictors

Below, we provide further justification for the selection of all candidate predictors, including descriptions of all assigned priors. For the sgRNA models (that predict sgRNA results from totRNA), we use γ/δ to signify parameters for the logistic component and α/β for the linear component. For the culture model (that predicts culture positivity from totRNA quantities), we use γ/ψ to signify the relevant parameters.

Primary predictors: We required all sgRNA models to include total RNA copy number (T) as a predictor, given this is the primary effect of interest. We expect the probability of detecting sgRNA to increase as total RNA copy numbers increase, which is supported by studies that find more sgRNA positive samples for those with large quantities of total RNA (7,8). Given that we expect this relationship to be positive, we assign the following prior: $\delta_T \sim N(2,1)$. We also expect sgRNA copy numbers to increase with total RNA copy numbers, as observed in other studies that find positive linear relationships between them (8–10). We assign the following prior:

$\beta_T \sim Gamma(2,0.5)$. We evaluated culture models including total RNA, sgRNA (SG), or both as primary predictors. We expect the likelihood of detecting infectious virus to increase with increasing quantities of either RNA type, so we set the priors to be: $\Psi_T, \Psi_{SG} \sim N(2, 1)$.

Age, sex, and non-human primate species: As with other viruses, age (AGE) and sex (SEX) are hypothesized to affect individual responses to SARS-CoV-2 infection, including within-host infection kinetics and disease severity. Inter-species variability in infection dynamics has also been noted among non-human primate species (SP) and other animal models (11). These differences may affect observed relationships between assays. However, given the complexity of these biological interactions, we do not assign *a priori* expectations about the direction of these effects, and we set all associated priors to be $N(0,1)$.

Inoculation dose: Since sgRNA is not typically packaged into virions (12) and thus should not meaningfully exist in viral stocks, we expect to find detectable levels of total RNA earlier than sgRNA after inoculation (i.e., lower probabilities of sgRNA detection per total RNA quantity for higher doses; $\delta_{DOSE} \sim N(-1,0.5)$). Larger inoculum doses could also result in (at least initially) higher levels of total RNA relative to sgRNA ($\beta_{DOSE} \sim N(-0.25,1)$). Exposure dose affects virion quantity and hence also total RNA quantity, which in turn may affect the probability of culture positivity through the presence of residual inoculum or other effects on infection kinetics. We make no *a priori* predictions on the direction of this effect ($\Psi_{DOSE} \sim N(0,1)$).

Day post infection: sgRNA levels likely remain low for some time in recently infected tissues. They may rise as virions infect local cells and replicate, and the ratio between total RNA and sgRNA may stabilize (8). These dynamic changes may be especially pronounced when large quantities of virions (and thus also genomic RNA) are introduced simultaneously, as in animal infection experiments. Consequently, we expect newly infected tissues to contain small, likely undetectable quantities of sgRNA, whereas tissues infected one or more days prior, which have experienced sufficient replication, likely contain more sgRNA copies. For infectious virus, residual inoculum-derived virus may actually increase the probability of culture positivity in infected tissues relative to non-inoculated tissues (where virions must be produced). We derived a categorical predictor with three levels: DPI[1], DPI[2], and DPI[3]. For inoculated tissues, we distinguish between the first day (DPI[1]) and all other days post infection (DPI[2]). Since infection timing is unknown for non-inoculated tissues, we group all days into one level (DPI[3]). **S3 Table** lists which tissues were considered inoculated for each exposure procedure. We used the following priors: $\delta_{DPI[1]} \sim N(-1,1)$, $\delta_{DPI[2]} \sim N(1,1)$, $\delta_{DPI[3]} \sim N(0,1)$, $\beta_{DPI[1]} \sim N(-0.5,1)$ $\beta_{DPI[2]} \sim N(0.5,1)$, $\beta_{DPI[3]} \sim N(0,1)$, $\Psi_{DPI[1]} \sim N(1,1)$, $\Psi_{DPI[2]} \sim N(0.5,1)$, $\Psi_{DPI[3]} \sim N(0,1)$.

Sample type: Differences in the processing and content of non-invasive (e.g., swabs, biofluids) and invasive (e.g., whole tissues obtained at necropsy) samples may affect assay readouts, and so we include sample type as a candidate predictor. We make no *a priori* predictions on their differences, and instead we assign non-informative priors ($\delta_{ST}, \beta_{ST}, \Psi_{ST} \sim N(0,1)$).

Target gene: The RT-qPCR target gene affects quantification of SARS-CoV-2 copy numbers (8,10,13,14), which stems from the nature of PCR in addition to the organizational structure and transcription mechanisms of the coronavirus genome. The production and abundance of sgRNA species varies by gene (e.g., sgRNA N > sgRNA E (15)), and sgRNA assays only amplify gene-specific sgRNA transcripts. In contrast, total RNA assays can amplify not only full-length genomic RNA and gene-specific sgRNA, but also other, larger sgRNAs that contain the target sequence. Given the many possible combinations of target gene pairs for total RNA and sgRNA assays, in our pooled dataset, we derived a generalizable predictor based on the number of transcripts available for amplification for each protocol. We distinguished between totRNA assays that amplify most (i.e., targets the Nucleocapsid gene; termed 'totRNA-high') or few sgRNA species (i.e., targets the Envelope gene; 'totRNA-low'). We also distinguish between sgRNA assays that target highly expressed (i.e., sgN; 'sgRNA-high') or less expressed sgRNA species (i.e., sgE, sg7; 'sgRNA-low'). This predictor thus has the following four levels: (1) totRNA-high/sgRNA-high, (2) totRNA-low/sgRNA-high, (3) totRNA-high/sgRNA-low, and (4) totRNA-low/sgRNA-low. We expect sgRNA-high assays to have higher probabilities of sgRNA detection and higher quantities of sgRNA per totRNA quantity ($\delta_{TG[1],}$ $\delta_{TG[2]}$~$N(1,1)$; $\beta_{TG[1],}$ $\beta_{TG[2]}$~$N(0.5,1)$) than sgRNA-low assays ($\delta_{TG[3],}$ $\delta_{TG[4]}$~$N(-1,1)$; $\beta_{TG[3],}$ $\beta_{TG[4]}$~$N(-0.5,1)$). For all culture models containing total RNA, we simply used the total RNA target gene as the predictor. When predicting culture from sgRNA, we used the sgRNA target gene as the predictor. Given SARS-CoV-2's genomic structure, we expect decreasing per-sample viral loads for totRNA protocols targeting the Nucleocapsid ($\Psi_{TG[1]}$~$N(-1,1)$), Envelope ($\Psi_{TG[2]}$~$N(0,1)$), and Spike ($\Psi_{TG[3]}$~$N(1,1)$) genes, respectively. Since these stoichiometric ratios apply in any infected cell, we expect the relative probability of culture positivity to be higher for target genes with lower expression, such that the probability is highest for Spike ($\Psi_{TG[3]}$~$N(1,1)$), intermediate for Envelope ($\Psi_{TG[2]}$~$N(0,1)$), and lowest for Nucleocapsid ($\Psi_{TG[1]}$~$N(-1,1)$).

Cell line: Viral infectivity can vary by cell line, which can influence the likelihood of detecting infectious virus in a sample. Our data includes three distinct cell lines (VeroE6, Vero76, VeroE6-TMPRSS2) which we group into a three-level categorical predictor (CELL) for the culture model only. Given evidence of increased SARS-CoV-2 entry in cells expressing TMPRSS2 (16,17), we expect the probability of detection to be highest for VeroE6-TMPRSS2 cells ($\Psi_{CELL[3]}$~$N(1,1)$). We use the same non-informative prior for the other two ($\Psi_{CELL[1]}$~$N(0,1)$, $\Psi_{CELL[2]}$~$N(0,1)$).

Culture assay: The sensitivity of endpoint dilution (TCID50) and plaque assays can also vary, with plaque assays typically having lower sensitivity (18). We distinguish between these protocols in a binary predictor (ASSAY), with endpoint dilution treated as the reference. We assign the following prior (Ψ_{ASSAY} ~$N(-0.5,1)$).

Article and lab effects: We assigned the same non-informative priors for the mean and standard deviation of each article's error term in the linear component ($\overline{\sigma} \sim N(0, 1)$; $\sigma_{sd} \sim Exp(1)$). We also assigned the same non-informative priors for all lab effect terms ($N(0, 0.5)$).

Approximate leave-one-out cross-validation

Beyond standard 10-fold cross-validation used in our main analyses, we also evaluated model performance and conducted model selection using Pareto-Smoothed Importance Sampling Approximate Leave-One-Out cross-validation (PSIS-LOO) via the 'loo' R package (19). We ran this method for the linear component of the sgRNA model using RStan version 2.21.0 to use additional functionality of the LOO software (20). All other models were run using CmdStan, as described in the main **Methods**.

Pareto-k values indicate the accuracy of PSIS-LOO approximations, where values below 0.7 indicate sufficiently reliable estimates. We use the moment-matching functionality offered by the 'loo' package to correct Pareto-k values exceeding 0.7, after which all PSIS-LOO estimates generated in this study were reliable (adjusted Pareto-k < 0.7).

Cross-validation methods and model evaluation metrics

For 10-fold cross-validation, we assigned folds randomly, although we required each fold to contain similar quantities of data from each article. This allowed us to distribute protocols, demographics, and sampling types more evenly across folds. All statistics for this method were calculated separately for training and test sets to evaluate performance on out-of-sample data and assess potential bias or overfitting, with the exception of ELPD and MCC which were only calculated for test sets.

As outlined in the model description of the main **Methods**, the linear component of the sgRNA models contained article-specific hierarchical error rates. We used the estimates of each article's specific error distribution to generate ELPD values. However, when we calculated median absolute error and the percent of samples falling within given prediction intervals, we did not incorporate article-specific errors. Instead, for each iteration, we sampled the full range of estimated errors by sampling the error distribution of a random included article (uniformly distributed), so these statistics better correspond to performance on new data (e.g., new studies) where prior information on error distributions is unavailable.

Selection procedure and results for the best sgRNA model

In the paragraphs below, we describe our model selection procedure for the sgRNA model, which relied on various performance metrics for each component. Overall, this procedure clearly identified the best model for both the logistic and linear components of the sgRNA model (**Fig 2**). Parallel analyses conducted with Pareto-Smoothed Importance Sampling approximate leave-one-out cross-validation resulted in qualitatively similar outcomes and selection of the same model (**S2, S3 Table**). Sensitivity analyses confirmed qualitatively similar results between informative and non-informative priors for the model selection procedure and for the parameter estimates of the best model (**S12 Fig**). Prediction accuracy and median absolute error (MAE) showed minimal

differences between training and test datasets, for all PCR models considered, offering confidence in the models' generalizability (**S2, S3 Table**).

For the logistic component, the best model performed substantially better than the simple model for all three statistics considered. The best model had considerably higher ELPD (difference: 67.3; **Fig 2, S2 Table**). Prediction accuracy on test data increased overall by 3.4 percent, and MCC increased from 0.75 to 0.82 (**Fig 2, 3C; S2 Table**). The best model also correctly predicted sgRNA detectability with higher probability for more samples and had a substantially higher ELPD, both of which reflect higher certainty for true classifications (**Fig 2, 3C**). Prediction accuracy and Matthews correlation coefficient (MCC) indicate nearly identical performance of the best and full models (**Fig 2**, **S2 Table**). The difference between the estimated log pointwise predictive density (ELPD) for the best and full models falls within two standard errors of the difference (difference: 1.60; standard error: 3.50), again indicating similar model performance (21).

When predicting copy numbers for all sgRNA positive samples with the linear component, the best model showed better performance than the full and simple models on all three statistics considered. Predictive performance of the best model is substantially better than the simple model, with 55.0 versus 48.0 percent of samples falling within the 50 percent prediction interval (**Fig 2, S3 Table**) and a decrease in median prediction error from 0.58 to 0.44 (**Fig 3F**). The ELPD difference is also substantial (-186.2). Posterior predictive checks show a strong correlation between observed and median predicted sgRNA values for the best model (adjusted R^2=0.77), which further supports superior performance of the multiple regression model compared to the simple single regression model (adjusted R^2=0.68). Relative to the full model, the best model has higher ELPD (difference: -4.44) and similar prediction error on test data (0.44 vs. 0.45) (**Fig 2, S3 Table**). A higher percent of (test) samples fall within the model-generated 50 percent prediction interval for the best model (55.0 vs. 54.5).

Selection procedure and results for the best culture model

In the paragraphs below, we describe our model selection procedure for the culture model, which relied on three primary performance metrics. This procedure clearly identified the best culture model. Model selection did not vary between cross-validation methods. Prior choice also did not alter model selection nor did it qualitatively affect parameter estimates (**Fig S12**). Prediction accuracy between training and test sets was comparable (**S4 Table**), again supporting model generalizability.

The evaluation procedure identified the best model as the one containing all but two candidate predictors (**Fig 2**). For all three statistics considered, the best model performed better than the simple model. The best model had considerably higher ELPD, for which the difference was larger than two standard errors (difference: 55.70; standard error: 13.96; **Fig 2, S4 Table**). For totRNA-positive samples, prediction accuracy on test data increased overall by 3.1 percent, but notably the best model correctly classified an additional 7.0 percent of culture positive samples (**Fig 4C**), both of which are reflected in the improvement of MCC from 0.48 to 0.57 (**S4 Table**). The

difference between the ELPD for the best and full models is small (difference: 0.76; standard error: 3.42), and their overall prediction accuracy and MCC are nearly identical (**Fig 2**, **S4 Table**).

Mathematical form of the best sgRNA model

The mathematical form of the best sgRNA model is outlined below, where we use γ/δ to signify parameters for the logistic component and α/β for the linear component. Acronyms are as follows: SG: sgRNA, T: total RNA, DOSE: inoculation dose (log10 pfu), SP: species, TG: target gene (standardized to four levels), and DPI: day post infection.

$$SG_{>LOD,i} \sim Bernoulli(p_i)$$
$$logit(p_i) = \gamma + \delta_T T_i + \delta_{DOSE} DOSE_i + \delta_{TG_i} + \delta_{SP_i}$$
$$SG_{value,i} \sim N(y_i, \sigma_{article_i})$$
$$y_i = \alpha + \beta_T T_i + \beta_{DOSE} DOSE_i + \beta_{TG_i} + \beta_{DPI_i} + \beta_{SP_i}$$
$$\sigma_{article_i} \sim N(\bar{\sigma}, \sigma_{sd})$$

Mathematical form of the best culture model

The mathematical form of the best culture model is outlined below, where we use γ/ψ to signify the relevant parameters. Acronyms are as follows: C: culture, T: total RNA, DOSE: inoculation dose (log10 pfu), DPI: day post infection, AGE: age class, SP: species, CELL: culture cell line, ASSAY: culture assay, and TG: target gene (standardized to three levels).

$$C_{>LOD,i} \sim Bernoulli(p_i)$$
$$logit(p_i) = \gamma + \psi_T T_i + \psi_{DOSE} DOSE_i + \psi_{DPI_i} + \psi_{AGE_i} + \psi_{SP_i} +$$
$$\psi_{CELL_i} + \psi_{ASSAY} ASSAY_i + \psi_{TG_i}$$

Prediction intervals and parameter estimates

To generate prediction intervals for various statistics, including median probabilities of sgRNA or culture positivity and median predicted sgRNA copy numbers, we used all available post-warmup parameter samples from the best model. We used the same procedure to generate the fit lines shown in Figures 3 and 5. Each prediction is generated using grouped parameter samples (e.g., samples from the same chain and iteration) to preserve correlation structure.

Estimating differences between predicted and known outcomes

To compare median predicted sgRNA copies to observed sgRNA or totRNA copies, we modeled the distribution of each of these quantities as normally distributed random variables with unique means and variances.

We also compared totRNA quantities for culture samples with false negative, true positive, and true negative predictions by estimating the population means for each group (captured as a normally distributed variable). For both of these investigations, we calculated the median and quantiles of the distribution of differences between these populations means, where we subtracted estimates only from the same chain and iteration. We fit these Bayesian models using the same procedures as described in the **Methods.**

References

1. Cramer PE, Gentzel RC, Tanis KQ, Vardigan J, Wang Y, Connolly B, et al. Aging African green monkeys manifest transcriptional, pathological, and cognitive hallmarks of human Alzheimer's disease. Neurobiol Aging. 2018 Apr 1;64:92–106.

2. Lee JR, Choe SH, Kim YH, Cho HM, Park HR, Lee HE, et al. Longitudinal profiling of the blood transcriptome in an African green monkey aging model. Aging. 2020 Dec 3;13(1):846–64.

3. Simmons HA. Age-Associated Pathology in Rhesus Macaques (Macaca mulatta). Vet Pathol. 2016 Mar 1;53(2):399–416.

4. Darusman HS, Call J, Sajuthi D, Schapiro SJ, Gjedde A, Kalliokoski O, et al. Delayed response task performance as a function of age in cynomolgus monkeys (Macaca fascicularis). Primates. 2014 Apr 1;55(2):259–67.

5. Koo BS, Lee DH, Kang P, Jeong KJ, Lee S, Kim K, et al. Reference values of hematological and biochemical parameters in young-adult cynomolgus monkey (Macaca fascicularis) and rhesus monkey (Macaca mulatta) anesthetized with ketamine hydrochloride. Lab Anim Res. 2019 Dec;35(1):1–6.

6. Carter J, Saunders VA. Virology: Principles and Applications. John Wiley & Sons; 2007. 383 p.

7. Perera RAPM, Tso E, Tsang OTY, Tsang DNC, Fung K, Leung YWY, et al. SARS-CoV-2 Virus Culture and Subgenomic RNA for Respiratory Specimens from Patients with Mild Coronavirus Disease - Volume 26, Number 11—November 2020 - Emerging Infectious Diseases journal - CDC. 2020;26(11):2701–4.

8. Dimcheff DE, Valesano AL, Rumfelt KE, Fitzsimmons WJ, Blair C, Mirabelli C, et al. Severe Acute Respiratory Syndrome Coronavirus 2 Total and Subgenomic RNA Viral Load in Hospitalized Patients. J Infect Dis. 2021 Oct 15;224(8):1287–93.

9. van Kampen JJA, van de Vijver DAMC, Fraaij PLA, Haagmans BL, Lamers MM, Okba N, et al. Duration and key determinants of infectious virus shedding in hospitalized patients with coronavirus disease-2019 (COVID-19). Nat Commun. 2021 Jan 11;12(1):267.

10. Verma R, Kim E, Martínez-Colón GJ, Jagannathan P, Rustagi A, Parsonnet J, et al. SARS-CoV-2 Subgenomic RNA Kinetics in Longitudinal Clinical Samples. Open Forum Infect Dis. 2021 Jul 1;8(7):ofab310.

11. Chu H, Chan JFW, Yuen KY. Animal models in SARS-CoV-2 research. Nat Methods. 2022 Apr;19(4):392–4.

12. Escors D, Izeta A, Capiscol C, Enjuanes L. Transmissible Gastroenteritis Coronavirus Packaging Signal Is Located at the 5′ End of the Virus Genome. J Virol. 2003 Jul 15;77(14):7890–902.

13. Lieberman JA, Pepper G, Naccache SN, Huang ML, Jerome KR, Greninger AL. Comparison of Commercially Available and Laboratory-Developed Assays for In Vitro Detection of SARS-CoV-2 in Clinical Laboratories. J Clin Microbiol. 2020 Jul 23;58(8):e00821-20.

14. Moreira LVL, Luna LK de S, Barbosa GR, Perosa AH, Chaves APC, Conte DD, et al. Test on stool samples improves the diagnosis of hospitalized patients: Detection of SARS-CoV-2 genomic and subgenomic RNA. J Infect. 2021 May 1;82(5):186–230.

15. Kim D, Lee JY, Yang JS, Kim JW, Kim VN, Chang H. The Architecture of SARS-CoV-2 Transcriptome. Cell. 2020 May 14;181(4):914-921.e10.

16. Hoffmann M, Kleine-Weber H, Schroeder S, Krüger N, Herrler T, Erichsen S, et al. SARS-CoV-2 Cell Entry Depends on ACE2 and TMPRSS2 and Is Blocked by a Clinically Proven Protease Inhibitor. Cell. 2020;181(2):271–80.

17. Matsuyama S, Nao N, Shirato K, Kawase M, Saito S, Takayama I, et al. Enhanced isolation of SARS-CoV-2 by TMPRSS2-expressing cells. Proc Natl Acad Sci. 2020 Mar 31;117(13):7001–3.

18. Smither SJ, Lear-Rooney C, Biggins J, Pettitt J, Lever MS, Olinger GG. Comparison of the plaque assay and 50% tissue culture infectious dose assay as methods for measuring filovirus infectivity. J Virol Methods. 2013 Nov 1;193(2):565–71.

19. Vehtari A, Gelman A, Gabry J. Practical Bayesian model evaluation using leave-one-out cross-validation and WAIC. Stat Comput. 2017 Sep 1;27(5):1413–32.

20. Vehtari A, Gabry J, Magnusson M, Yuling Y, Bürkner PC, Paananen T, et al. loo: Efficient leave-one-out cross-validation and WAIC for Bayesian models [Internet]. 2022. Available from: https://mc-stan.org/loo/

21. Sivula T, Magnusson M, Matamoros AA, Vehtari A. Uncertainty in Bayesian Leave-One-Out Cross-Validation Based Model Comparison [Internet]. arXiv; 2022 [cited 2022 Nov 16]. Available from: http://arxiv.org/abs/2008.10296
