# Supplementary figures and images for: Predicting the presence of infectious virus from PCR data: A meta-analysis of SARS-CoV-2 in non-human primates
Source: PLoS Pathog. 2024 Apr 29;20(4):e1012171. doi: 10.1371/journal.ppat.1012171 (PMC11081500; doi:10.1371/journal.ppat.1012171)

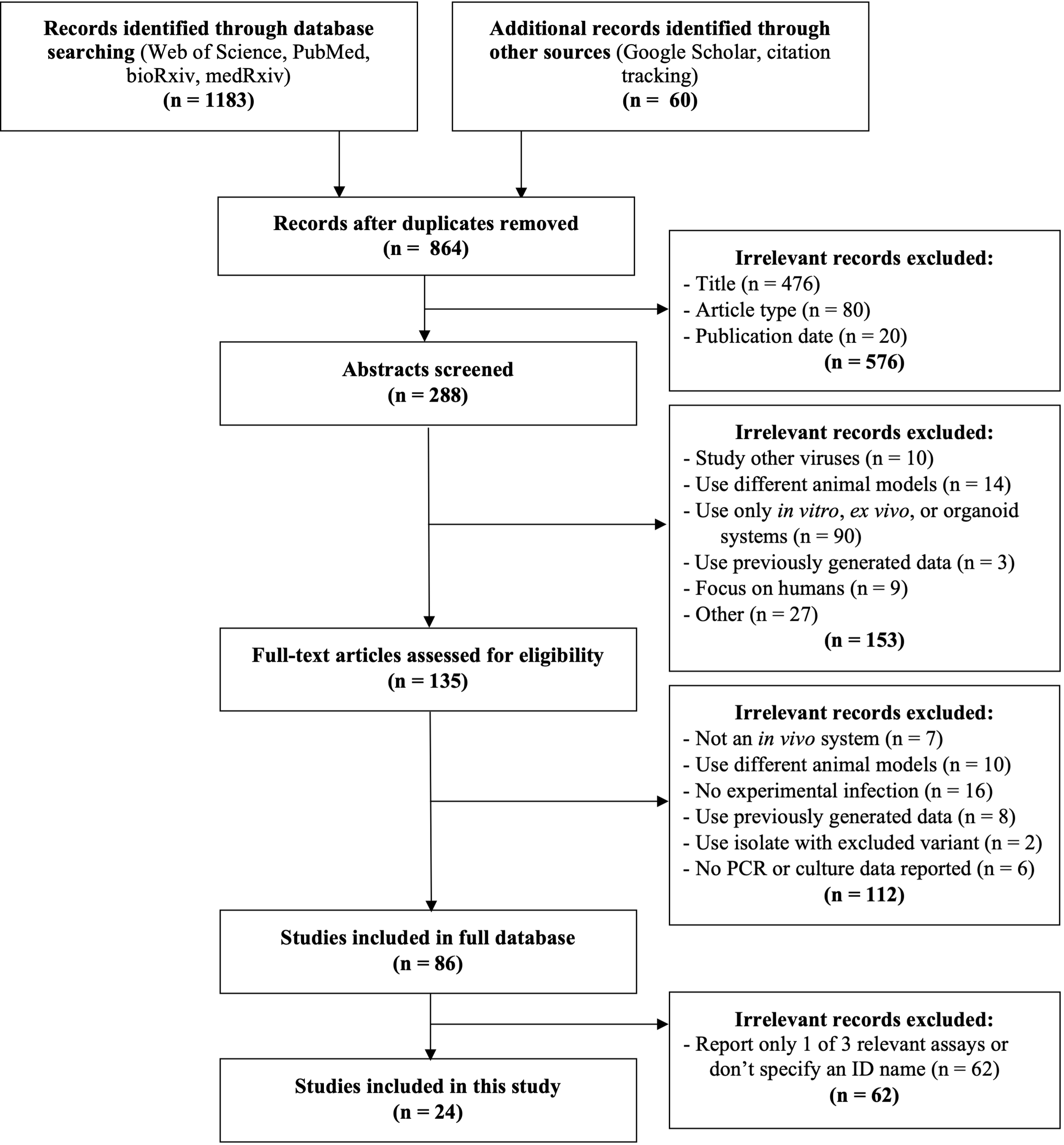

Supplement: S1 Fig — We created this figure by adapting the template flowchart provided in Moher et al. 2009 (34), which offers guidelines and resources for systematic reviews and meta-analyses. We incorporated all of their suggested steps for reporting the results of systematic literature searches, but all of the substantive content (e.g., numbers, exclusion reasons) is based entirely on our literature search. Additional detail on the screening procedure is provided in the S1 Methods. (TIF) [file ppat.1012171.s002.tif]

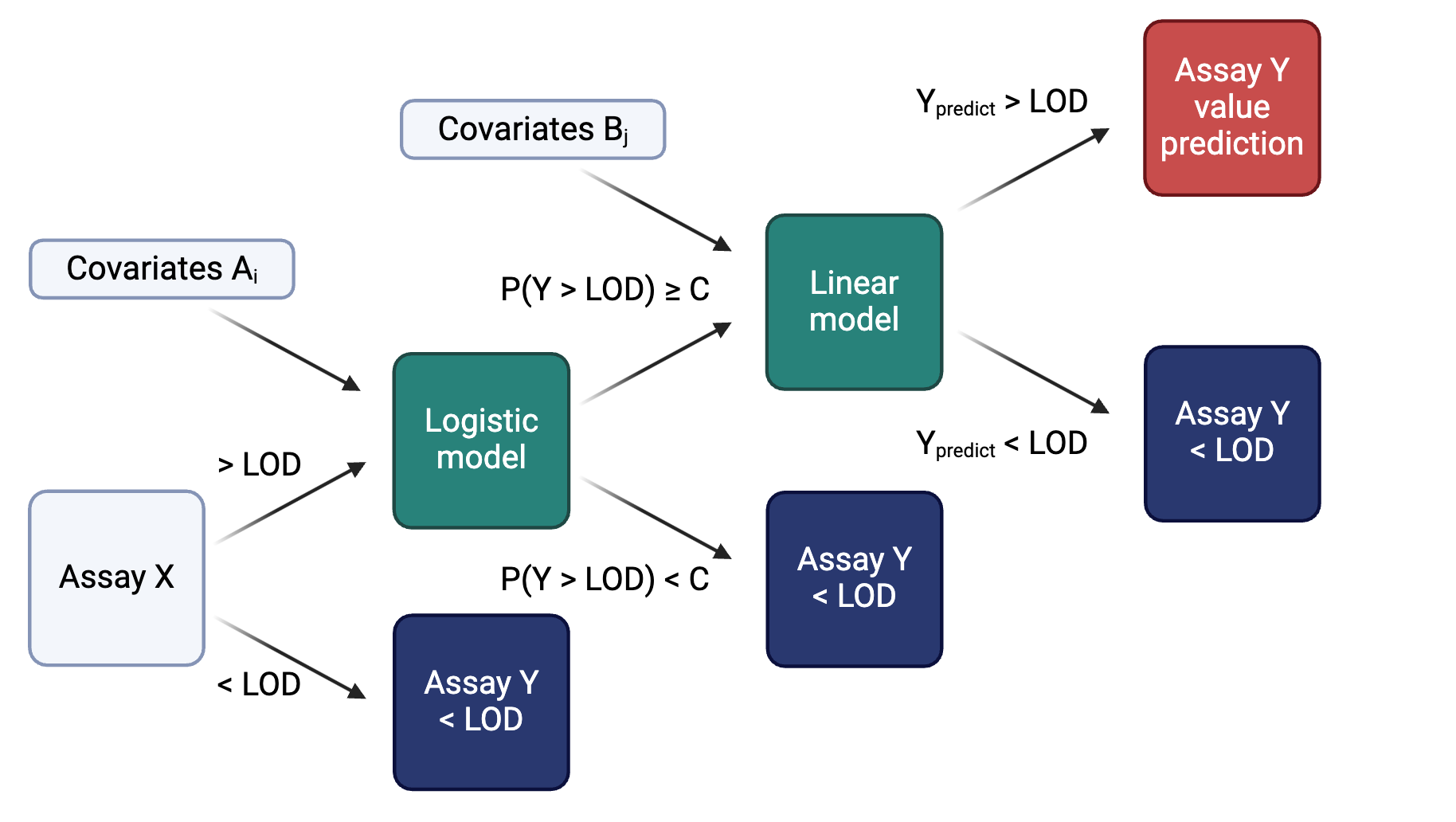

Supplement: S2 Fig — Predictors are grey, model components are green, and predictions are red (positive) or blue (negative). If assay X falls below the limit of detection (< LOD), assay Y is also predicted to fall below the limit of detection. (Note that this particular assumption may not hold for all assay relationships, and modeling adjustments may need to be made in these scenarios.) If assay X falls above the limit of detection (> LOD), then the value of assay X is passed as a predictor to the logistic component of the hurdle model, which uses a set of additional covariates Ai to predict whether assay Y falls above or below the LOD. If the posterior probability of assay Y falling above the limit of detection is less than some assigned threshold C (P(Y > LOD) < C), then the model predicts assay Y falls below the LOD. Otherwise, the model predicts assay Y falls above the LOD. Note that the probability cut-off value C should be selected to balance false positive and false negative rates as appropriate to investigator aims. In this study, we used a standard value of C = 0.5. For samples predicted to fall above the LOD, the linear model component will generate a predicted value of assay Y (Ypredict) based on another set of covariates (Bj). If Ypredict is larger than the reported LOD for assay Y, the model will return the predicted value. Created with BioRender.com. (TIF) [file ppat.1012171.s003.tif]

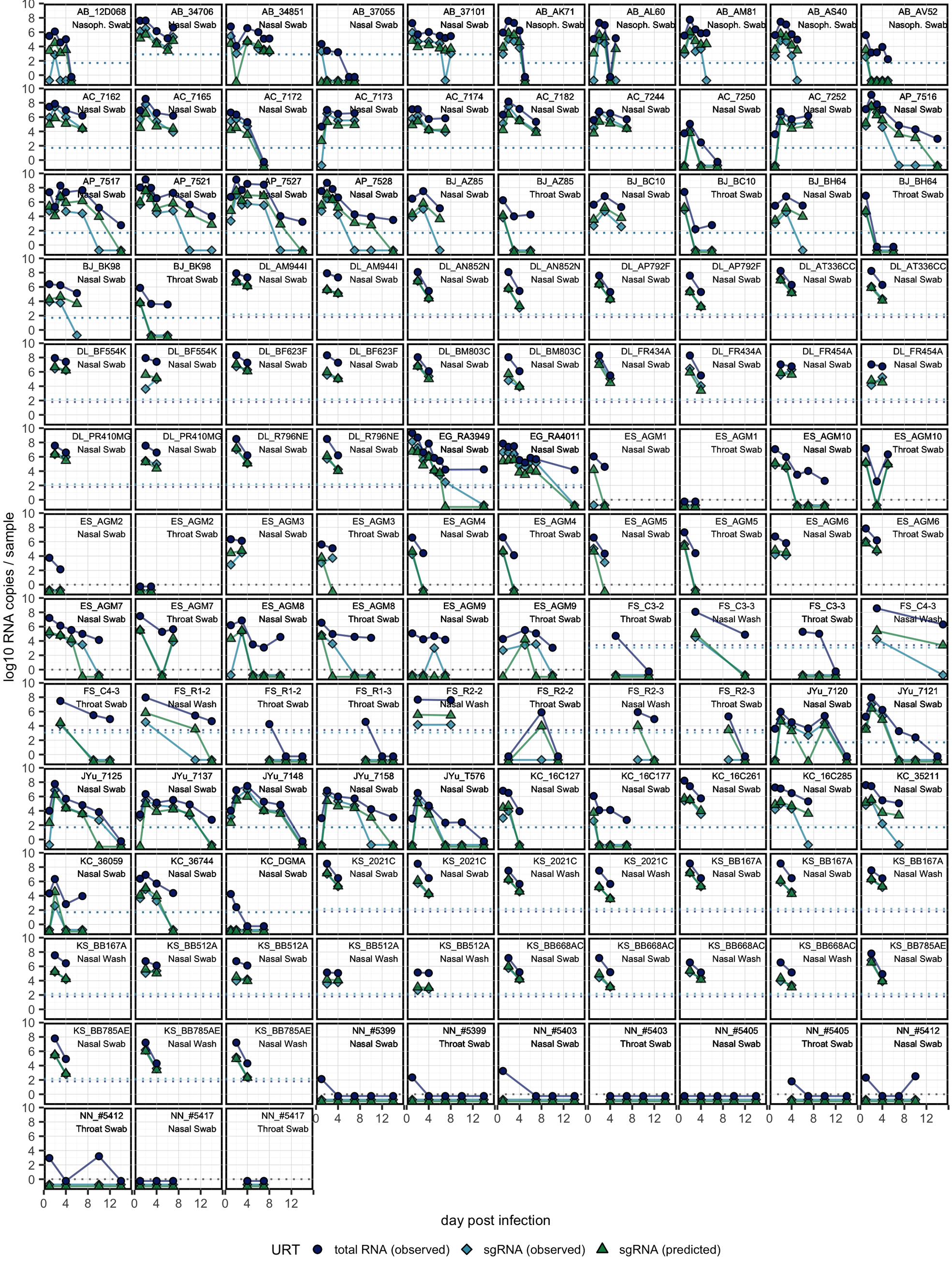

Supplement: S3 Fig — Each panel corresponds with one individual and one non-invasive sample type, indicated in the top right of each panel. Only individuals with both total RNA and sgRNA results for at least two days post infection are plotted. Some individuals were sampled from multiple locations in the upper respiratory tract, in which case they are plotted as neighboring panels. Each line and the accompanying points track the individual’s total RNA (dark blue, circle), observed sgRNA (light blue, diamond), and median predicted sgRNA (green, triangle) trajectories. For some individuals (e.g., KS_2021C), multiple RT-qPCR assays targeting different genes were run on the same sample, which are plotted as distinct panels. All samples observed or predicted to fall below the limit of detection are plotted below 0 at set values for visual clarity (totRNA: -0.5, observed sgRNA: -0.75, predicted sgRNA: -1). When available, the limits of detection (LOD) or quantification (LOQ) for PCR assays are plotted as dotted lines in the assay-specific color. When both the LOD and LOQ were available, only the LOD is plotted. In instances where the total RNA and sgRNA assay LOD are equal, only the sgRNA line is visible. No instances exist in this dataset where the LOD or LOQ is only available for one RNA type. (TIF) [file ppat.1012171.s004.tif]

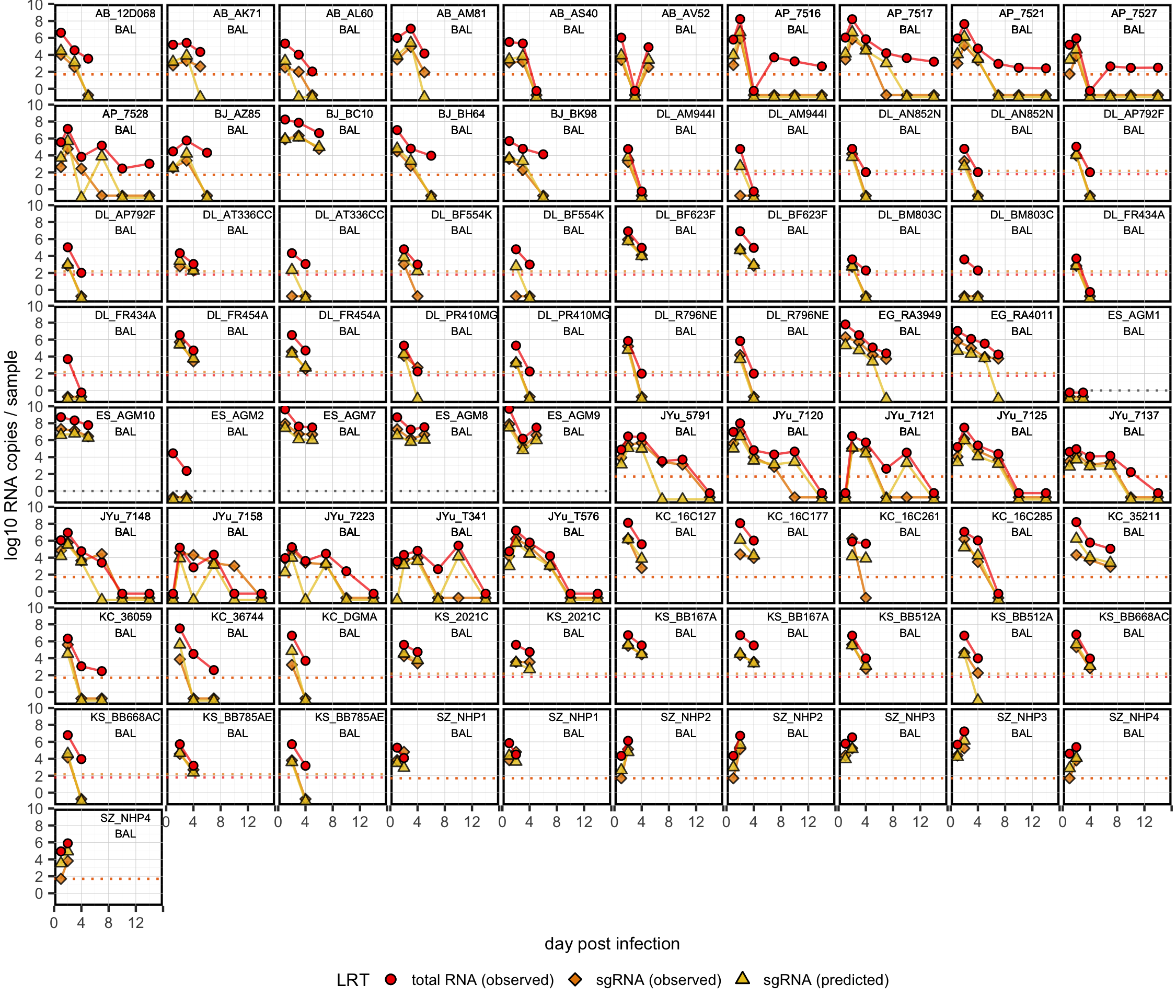

Supplement: S4 Fig — Each panel corresponds with one individual and one non-invasive sample type, indicated in the top right of each panel (‘BAL’: bronchoalveolar lavage). Only individuals with both total RNA and sgRNA results for at least two days post infection are plotted. Each line and the accompanying points track the individual’s total RNA (dark red, circle), observed sgRNA (orange, diamond), and median predicted sgRNA (yellow, triangle) trajectories. For some individuals (e.g., KS_2021C), multiple RT-qPCR assays targeting different genes were run on the same sample, which are plotted as distinct panels. All samples observed or predicted to fall below the limit of detection are plotted below 0 at set values for visual clarity (totRNA: -0.5, observed sgRNA: -0.75, predicted sgRNA: -1). When available, the limits of detection (LOD) or quantification (LOQ) for PCR assays are plotted as dotted lines in the assay-specific color. When both the LOD and LOQ were available, only the LOD is plotted. In instances where the total RNA and sgRNA assay LOD are equal, only the sgRNA line is visible. No instances exist in this dataset where the LOD or LOQ is only available for one RNA type. (TIF) [file ppat.1012171.s005.tif]

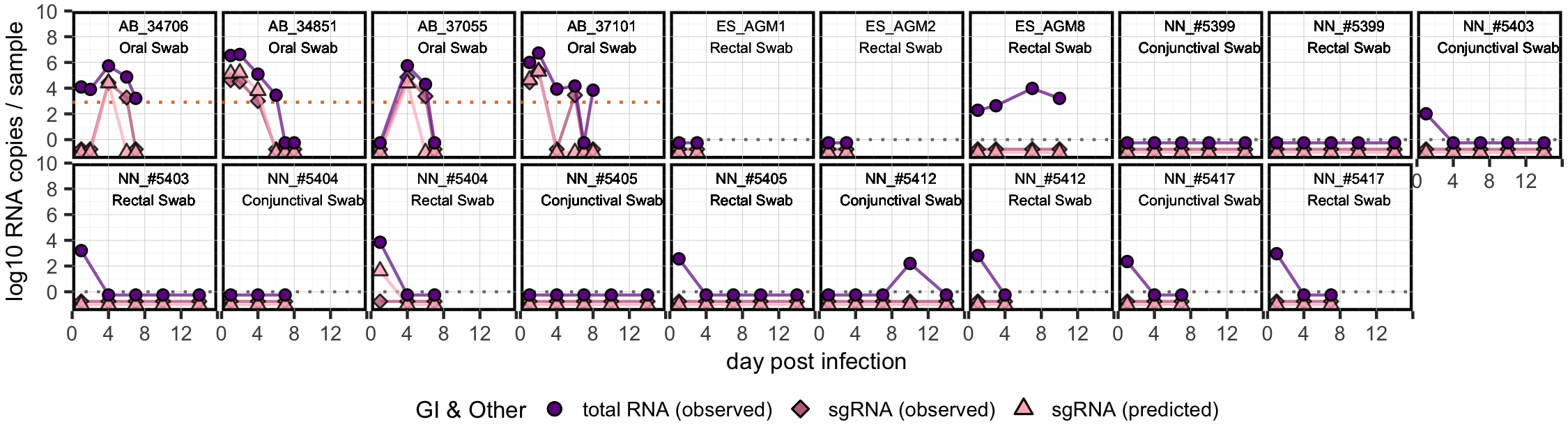

Supplement: S5 Fig — Each panel corresponds with one individual and one non-invasive sample type, indicated in the top right of each panel. Only individuals with both total RNA and sgRNA results for at least two days post infection are plotted. Each line and the accompanying points track the individual’s total RNA (dark purple, circle), observed sgRNA (dark pink, diamond), and median predicted sgRNA (light pink, triangle) trajectories. All samples observed or predicted to fall below the limit of detection are plotted below 0 at set values for visual clarity (totRNA: -0.5, observed sgRNA: -0.75, predicted sgRNA: -1). When available, the limits of detection (LOD) or quantification (LOQ) for PCR assays are plotted as dotted lines in the assay-specific color. When both the LOD and LOQ were available, only the LOD is plotted. In instances where the total RNA and sgRNA assay LOD are equal, only the sgRNA line is visible. No instances exist in this dataset where the LOD or LOQ is only available for one RNA type. (TIF) [file ppat.1012171.s006.tif]

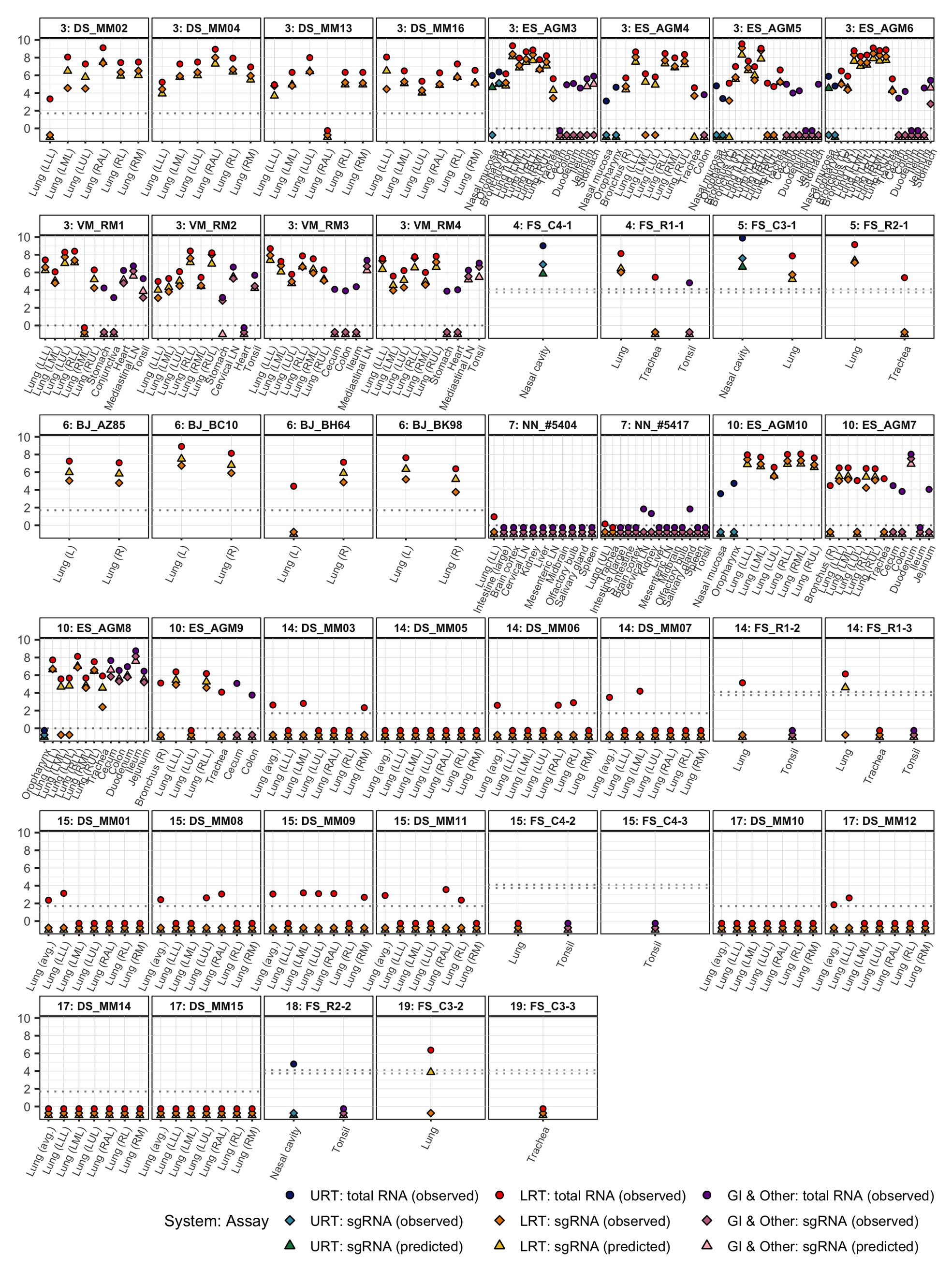

Supplement: S6 Fig — Each panel corresponds with one individual, indicated with text in the panel (day post infection: individual). Each point presents the total RNA (circle), observed sgRNA (diamond), and predicted sgRNA (triangle) values. All samples observed or predicted to fall below the limit of detection are plotted below 0 at set values for visual clarity (totRNA: -0.5, observed sgRNA: -0.75, predicted sgRNA: -1). When available, the limits of detection (LOD) or quantification (LOQ) for PCR assays are plotted as dotted lines in the assay-specific color. When both the LOD and LOQ were available, only the LOD is plotted. In instances where the total RNA and sgRNA assay LOD are equal, only the sgRNA line is visible. No instances exist in this dataset where the LOD or LOQ is only available for one RNA type. (TIF) [file ppat.1012171.s007.tif]

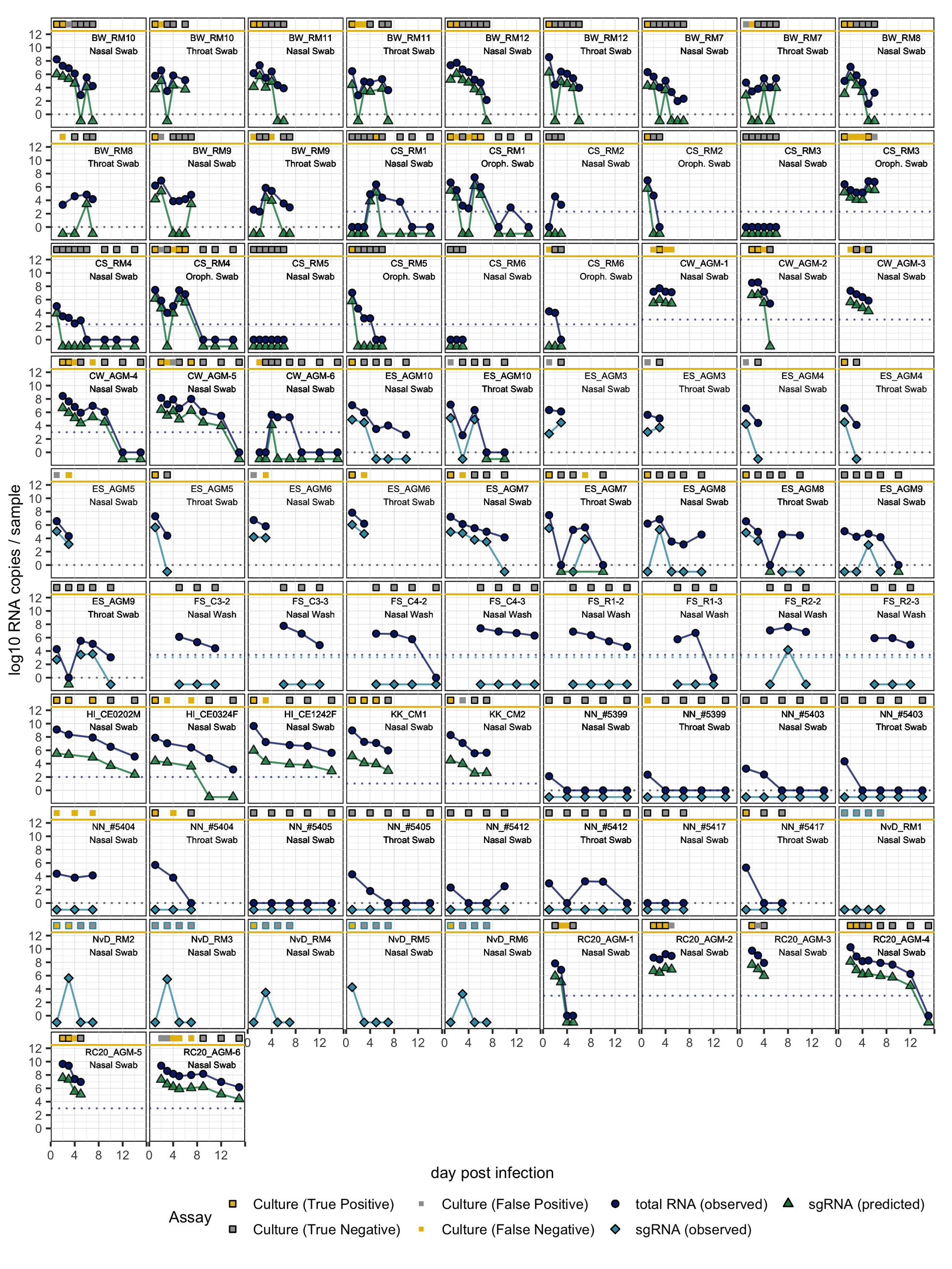

Supplement: S7 Fig — Each panel corresponds with one individual and one non-invasive sample type, indicated in the top right of each panel. Only individuals with culture results for at least two days post infection are plotted. Culture data are plotted as squares above the yellow line at 10 log10 copies. Yellow squares are culture positive samples, while grey squares are culture negative. Squares outlined in black are correct predictions, squares with no outline are incorrect predictions. We did not generate predictions for the culture samples outlined in blue, as they do not have available totRNA results. We also plot observed total RNA values (circle) and observed sgRNA values (diamond), otherwise we plot predicted median sgRNA values generated by our best sgRNA model (triangle). Some individuals were sampled from multiple locations in the upper respiratory tract, in which case they are plotted as neighboring panels. All samples observed or predicted to fall below the limit of detection are plotted below 0 at set values for visual clarity (totRNA: 0, sgRNA: -1). When available, the limits of detection (LOD) or quantification (LOQ) for PCR assays are plotted as dotted lines in the assay-specific color. When both the LOD and LOQ were available, only the LOD is plotted. In instances where the total RNA and sgRNA assay LOD are equal, only the sgRNA line is visible. No instances exist in this dataset where the LOD or LOQ is only available for one RNA type. Individuals from one study cannot be included in this figure due to a data sharing agreement. (TIF) [file ppat.1012171.s008.tif]

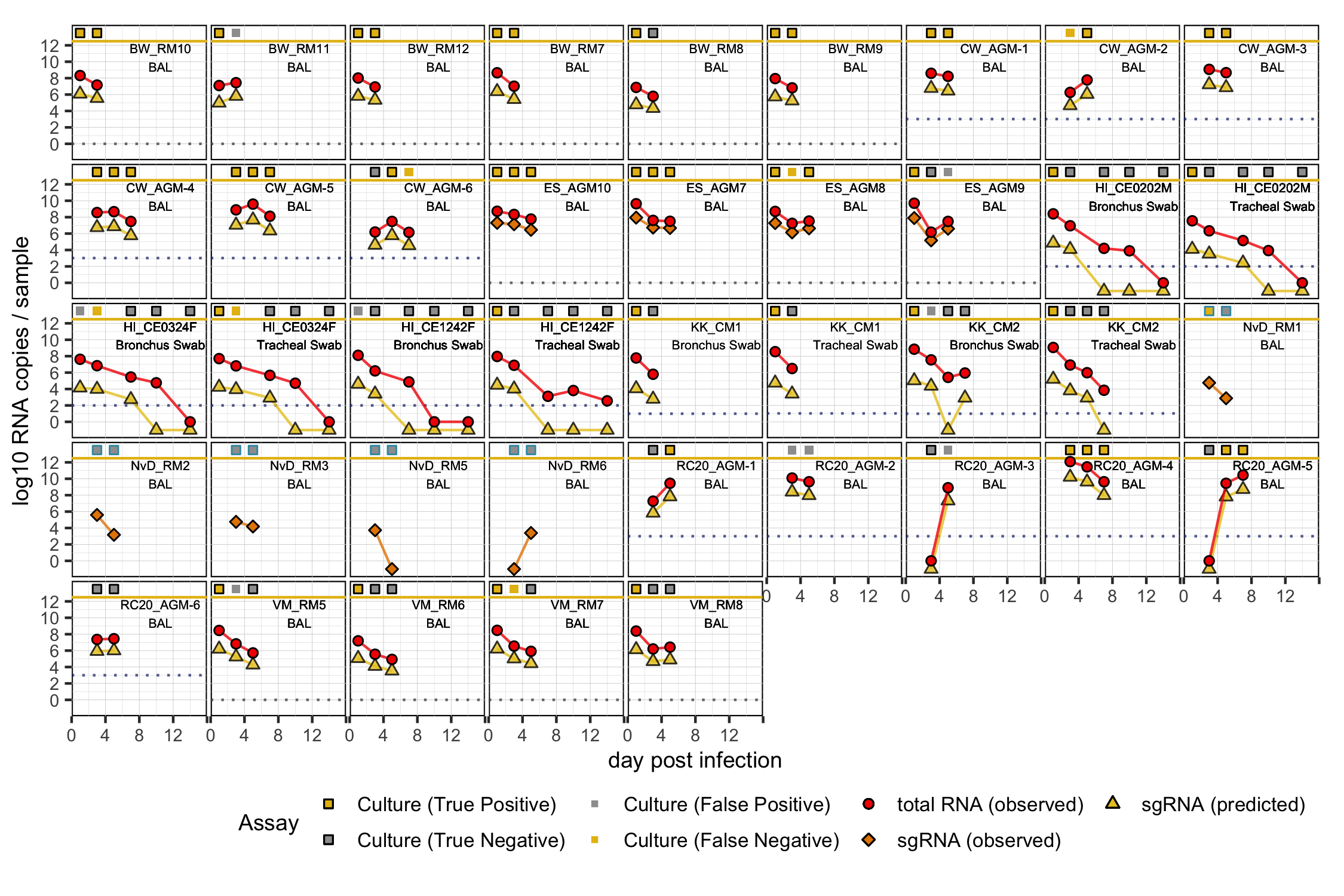

Supplement: S8 Fig — Each panel corresponds with one individual and one non-invasive sample type, indicated in the top right of each panel. Only individuals with culture results for at least two days post infection are plotted. Culture data are plotted as squares above the yellow line at 10 log10 copies. Yellow squares are culture positive samples, while grey squares are culture negative. Squares outlined in black are correct predictions, squares with no outline are incorrect predictions. We did not generate predictions for the culture samples outlined in blue, as they do not have available totRNA results. We also plot observed total RNA values (circle) and observed sgRNA values (diamond) when available, otherwise we plot predicted median sgRNA values generated by our best sgRNA model (triangle). Some individuals were sampled from multiple locations in the lower respiratory tract, in which case they are plotted as neighboring panels. All samples observed or predicted to fall below the limit of detection are plotted below 0 at set values for visual clarity (totRNA: 0, sgRNA: -1). When available, the limits of detection (LOD) or quantification (LOQ) for PCR assays are plotted as dotted lines in the assay-specific color. When both the LOD and LOQ were available, only the LOD is plotted. In instances where the total RNA and sgRNA assay LOD are equal, only the sgRNA line is visible. No instances exist in this dataset where the LOD or LOQ is only available for one RNA type. (TIF) [file ppat.1012171.s009.tif]

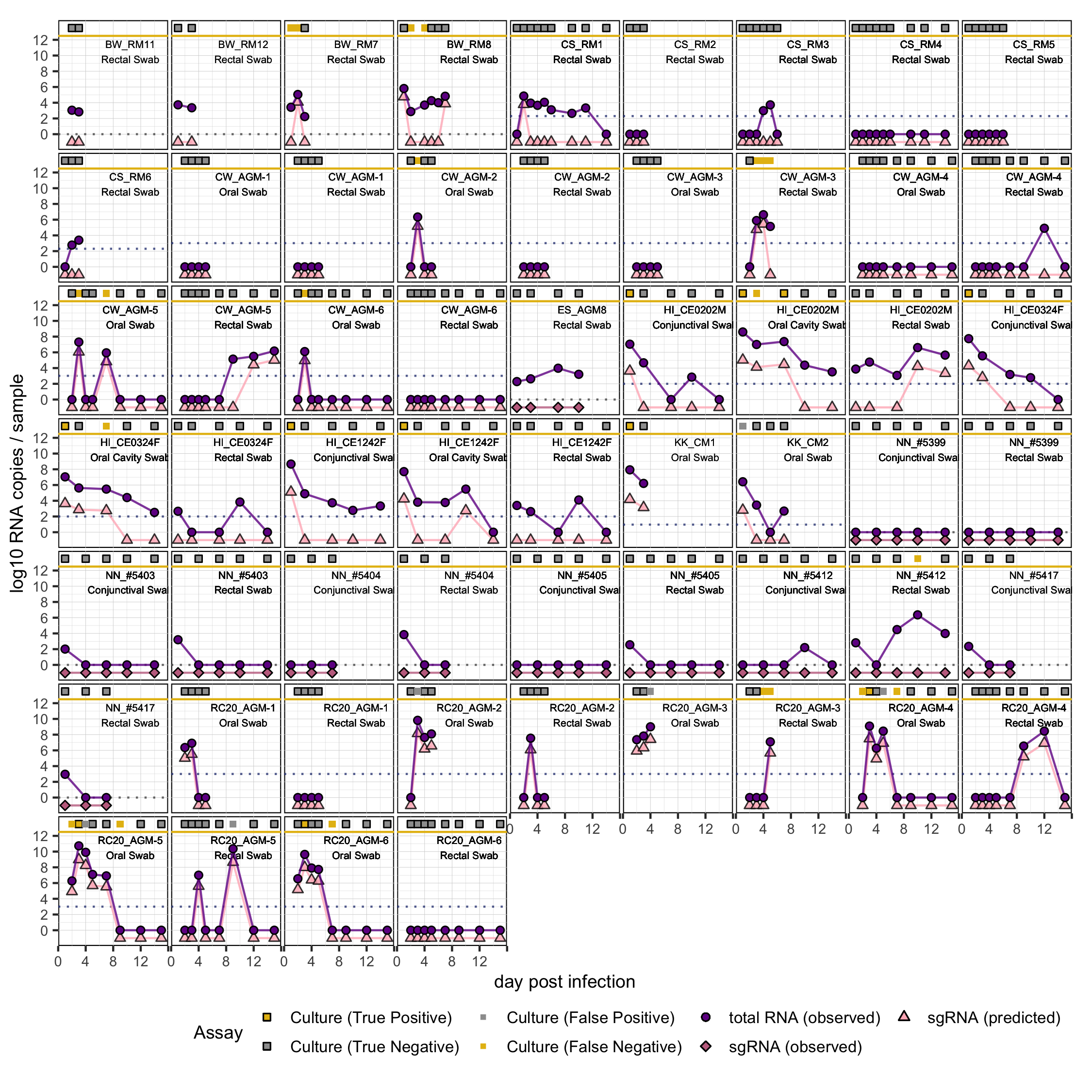

Supplement: S9 Fig — Each panel corresponds with one individual and one non-invasive sample type, indicated in the top right of each panel. Only individuals with culture results for at least two days post infection are plotted. Culture data are plotted as squares above the yellow line at 10 log10 copies. Yellow squares are culture positive samples, while grey squares are culture negative. Squares outlined in black are correct predictions, squares with no outline are incorrect predictions. We also plot observed total RNA values (circle) and observed sgRNA values (diamond) when available, otherwise we plot predicted median sgRNA values generated by our best sgRNA model (triangle). Some individuals were sampled from multiple locations, in which case they are plotted as neighboring panels. All samples observed or predicted to fall below the limit of detection are plotted below 0 at set values for visual clarity (totRNA: 0, sgRNA: -1). When available, the limits of detection (LOD) or quantification (LOQ) for PCR assays are plotted as dotted lines in the assay-specific color. When both the LOD and LOQ were available, only the LOD is plotted. In instances where the total RNA and sgRNA assay LOD are equal, only the sgRNA line is visible. No instances exist in this dataset where the LOD or LOQ is only available for one RNA type. Individuals from one study cannot be included in this figure due to a data sharing agreement. (TIF) [file ppat.1012171.s010.tif]

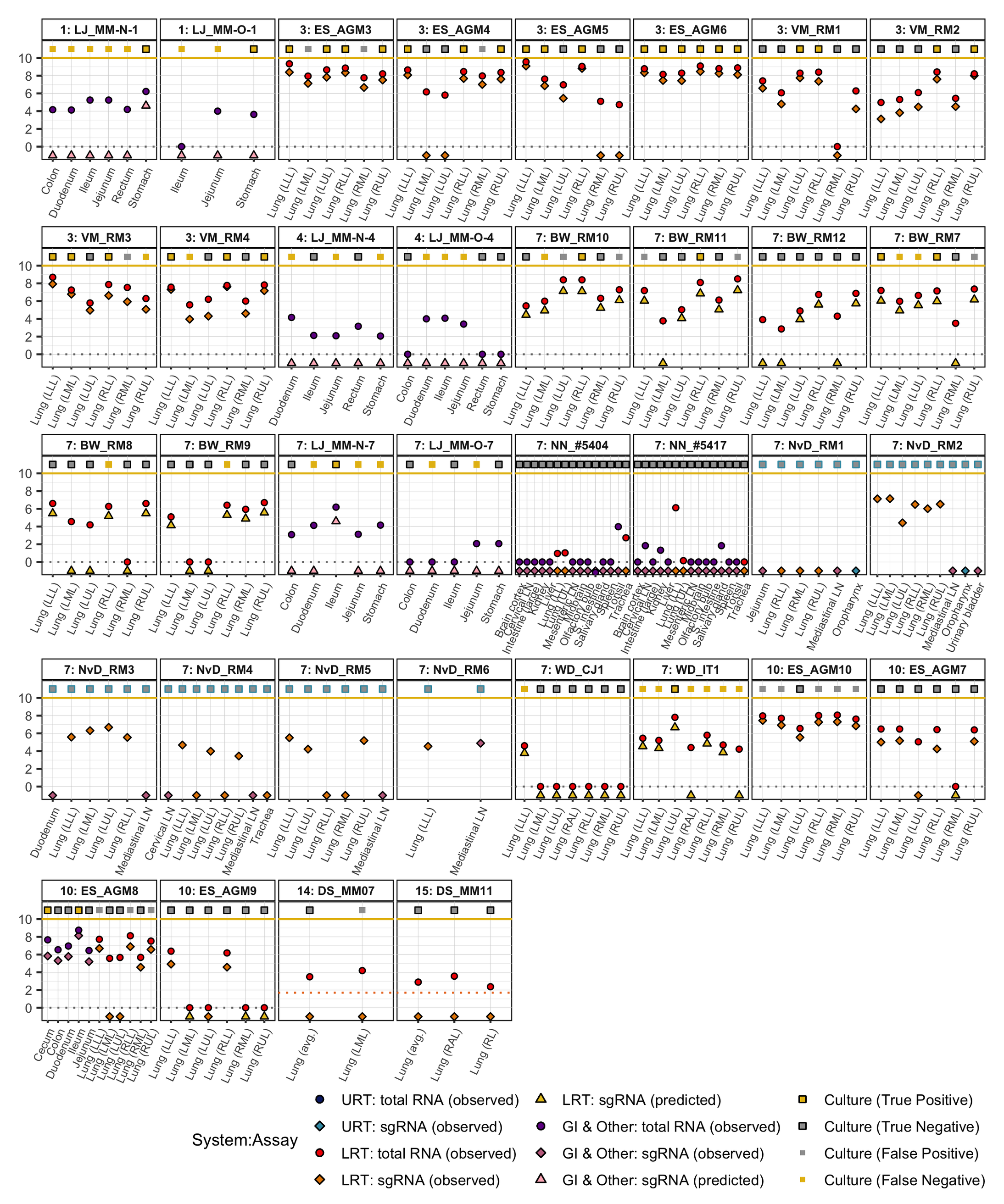

Supplement: S10 Fig — Each panel corresponds with one individual, indicated with text in the panel (day post infection: individual). Culture data are plotted as squares above the yellow line at 10 log10 copies. Yellow squares are culture positive samples, while grey squares are culture negative. Squares outlined in black are correct predictions, squares with no outline are incorrect predictions. We did not generate predictions for the culture samples outlined in blue, as they do not have available totRNA results. We also plot the observed total RNA (circle) and observed sgRNA (diamond) values when available, otherwise we plot predicted median sgRNA values generated by our best sgRNA model (triangle). Color corresponds to the organ system from which the tissue was obtained (URT, upper respiratory tract; LRT, lower respiratory tract; GI & Other, gastrointestinal and other systems). All samples observed or predicted to fall below the limit of detection are plotted below 0 at set values for visual clarity (totRNA: 0, sgRNA: -1). When available, the limits of detection (LOD) or quantification (LOQ) for PCR assays are plotted as dotted lines in the assay-specific color. When both the LOD and LOQ were available, only the LOD is plotted. In instances where the total RNA and sgRNA assay LOD are equal, only the sgRNA line is visible. No instances exist in this dataset where the LOD or LOQ is only available for one RNA type. (TIF) [file ppat.1012171.s011.tif]

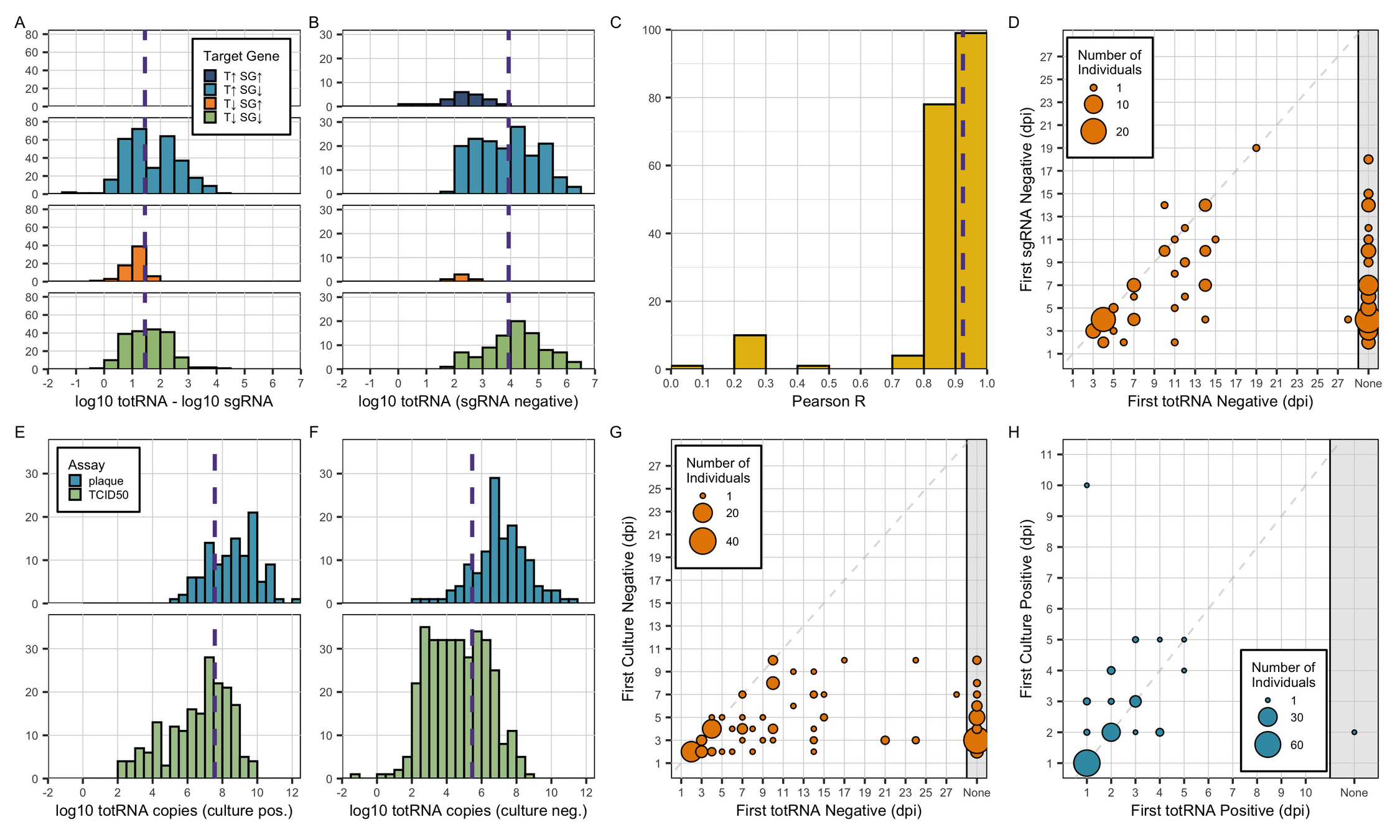

Supplement: S11 Fig — (A) Difference between total RNA and sgRNA copy numbers when both are detectable, stratified by target gene predictor with the following acronyms: “T↑SG↑”: totRNA-high/sgRNA-high; “T↓SG↑”: totRNA-low/sgRNA-high; “T↑SG↓”: totRNA-high/sgRNA-low; “T↓SG↓”: totRNA-high/sgRNA-low. No totRNA-high/sgRNA-high data was available for this investigation. (B) Total RNA copy numbers for all sgRNA negative samples, stratified by target gene as in (A). (C) Pearson correlation coefficients between total RNA and sgRNA copy numbers when both are detectable, for all individual-sample trajectories with at least three sampling days where both were positive. (D) Comparison of the timing of the first negative results from total RNA and sgRNA assays for each available individual-sample trajectory (dpi: day post infection). (E) Total RNA copy numbers (when detectable) for all culture positive samples, stratified by culture assay type. (F) Total RNA copy numbers (when detectable) for all culture negative samples, stratified by culture assay type as in (E). (G) Comparison of the timing of the first negative results from total RNA and culture assays for each available individual-sample trajectory. (H) Comparison of the timing of the first positive results from total RNA and culture assays for each individual-sample trajectory. For panels (A), (B), (C), (E), and (F), the purple dashed line indicates the median for the full distribution (i.e., not stratified by assay or target gene). For panels (D), (G), and (H), the size of each circle indicates the number of individuals with the indicated observation. Individuals in the ‘None’ column were never negative (D, G) or positive (H) for total RNA. Individuals that were never sgRNA negative (D), culture negative (G), or culture positive (H) are not plotted. (TIF) [file ppat.1012171.s012.tif]

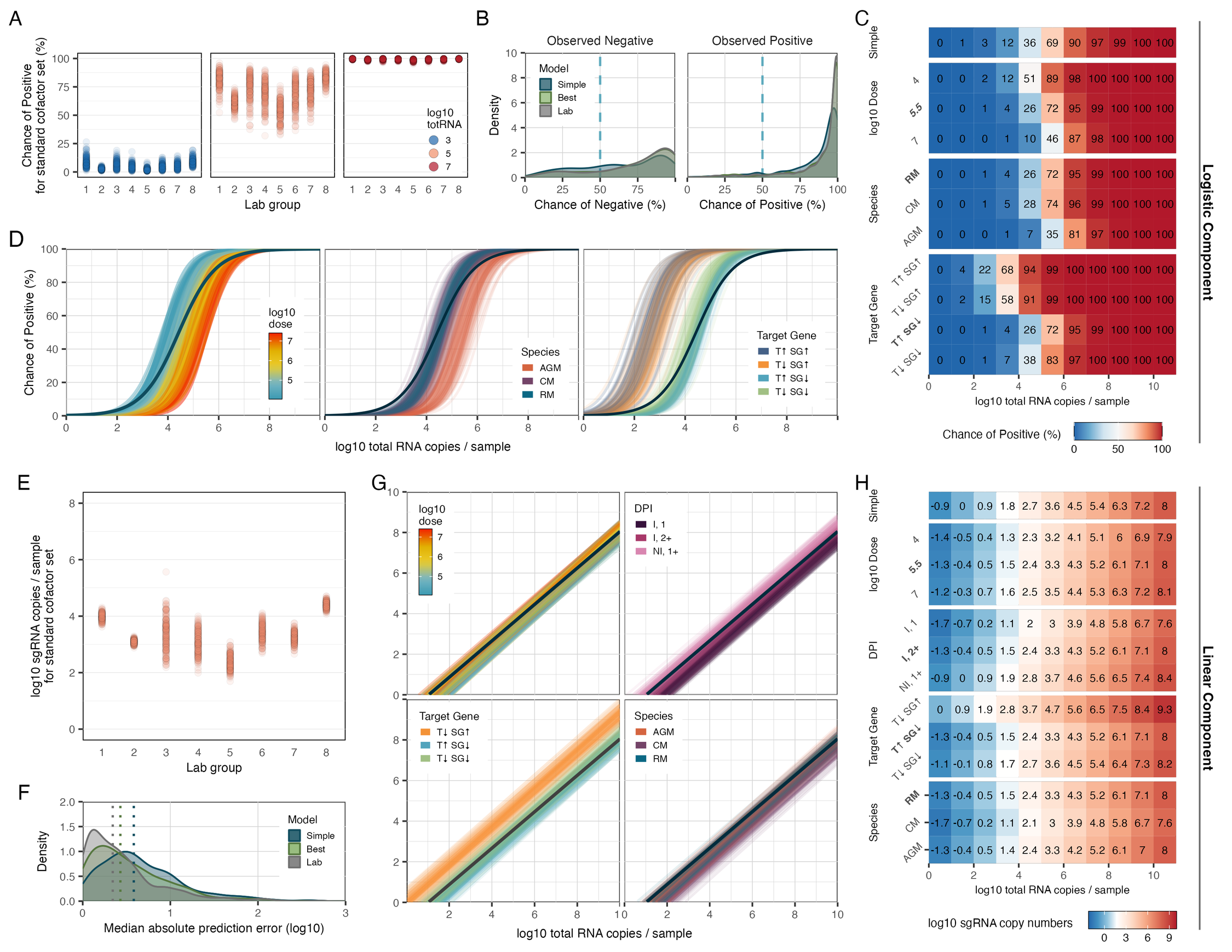

Supplement: S12 Fig — (A) The predicted chances of sgRNA detection for three key totRNA quantities (3 log10, blue; 5 log10, salmon; 7 log10, red), across the eight available lab groups and for the standard cofactor set. The article(s) included in each group are provided in S8 Table. Each point is one out of 200 samples generated for each lab group, with transparency to show the density of points. (B) As in Fig 3B, with additional predictions from the model including a lab effect (‘Lab’, grey). (C and D) As in Fig 3C and 3D, except showing the results from the model including a lab effect. (E) The predicted quantities of sgRNA for a sample with 5 log10 totRNA copies, across the eight available lab groups and for the standard cofactor set. (F) As in Fig 3F, with additional predictions from the model including a lab effect (‘Lab’, grey). (G and H) As in Fig 3G and 3H, except showing the results from the model including a lab effect. In panels C, D, G and H, the predictions are not specific to a particular lab group (i.e., we set the lab effect term to zero to extract general patterns across all labs). (TIF) [file ppat.1012171.s013.tif]

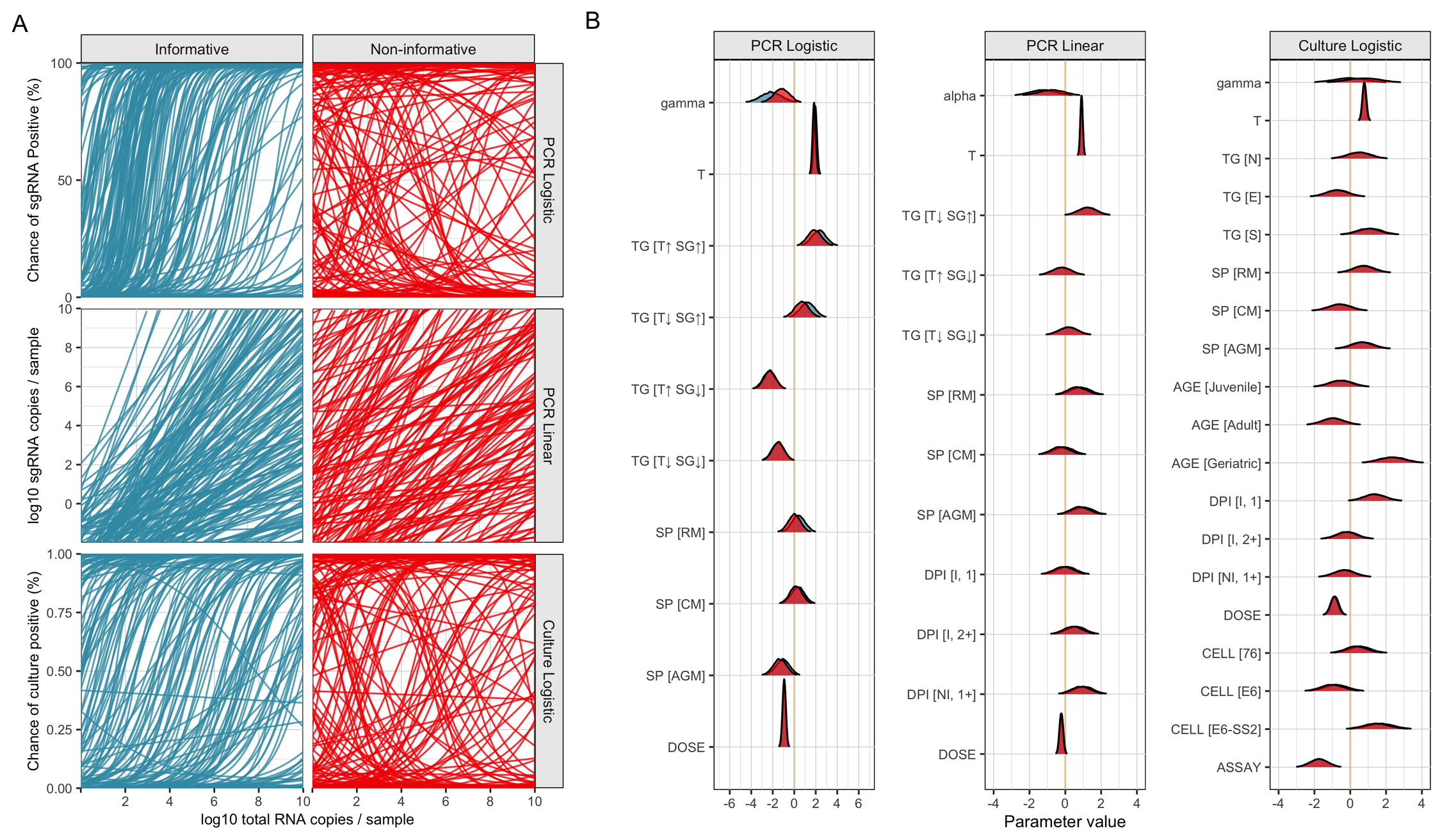

Supplement: S13 Fig — (A) Each line presents an expected model fit generated by sampling the indicated prior distributions. Informative priors are outlined in the Methods and S1 Methods. All parameters were given a N(0,1) prior for all non-informative investigations. Informative priors much better represent a priori understanding of the relationships between total RNA copy numbers and both sgRNA and culture outcomes. (B) Each panel compares the final parameter estimates obtained for the corresponding best model using the different prior types (red: non-informative; blue: informative), where each row is a distinct parameter. Acronyms are as described in Figs 3 and 5. Note that in many instances parameters estimates are almost perfectly overlapping, so only the non-informative (red) priors are visible. (TIF) [file ppat.1012171.s014.tif]

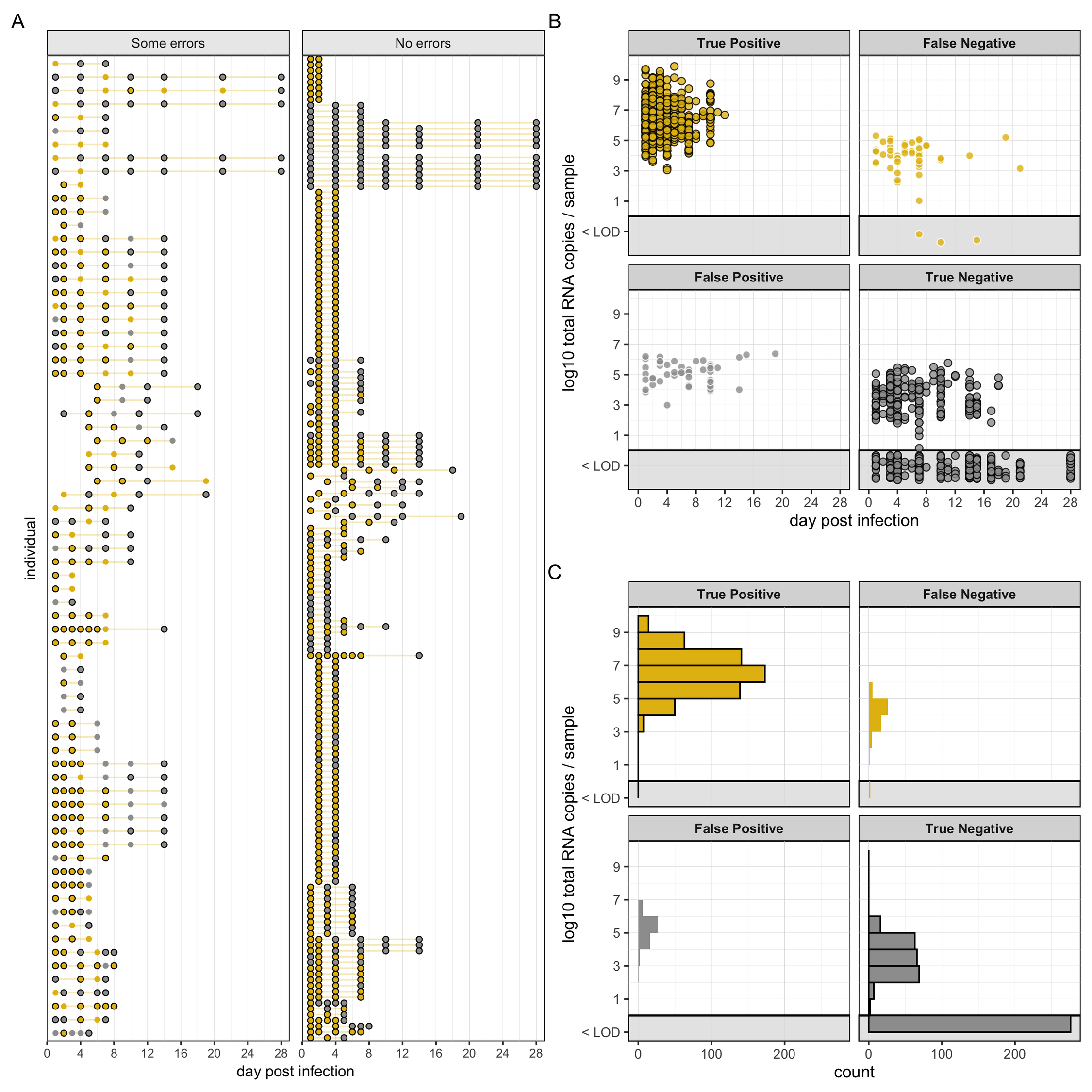

Supplement: S14 Fig — (A) Individual-specific sgRNA trajectories, where each row presents one individual. These are stratified by whether the model misclassifies any samples for that individual (“Some errors”) or whether the model makes no misclassifications (“No errors”). In both (A) and (B), yellow circles indicate positive samples and grey indicates negative samples. Circles with a black outline correspond with correctly classified samples, while no outline indicates incorrectly classified samples. (B) Scatterplot of all samples with sgRNA results, stratified by the elements of a confusion matrix and colored as in (A). The x-axis tracks the day post infection and the y-axis plots log10 total RNA copy numbers. Samples in the grey shaded region along the bottom present all samples where total RNA was undetectable. (C) Histograms of all samples grouped by the elements of a confusion matrix, where log10 total RNA copy numbers per sample is plotted on the y-axis. Bins located in the grey shaded region along the bottom (labelled “<LOD”) include all totRNA-negative samples. (TIF) [file ppat.1012171.s015.tif]

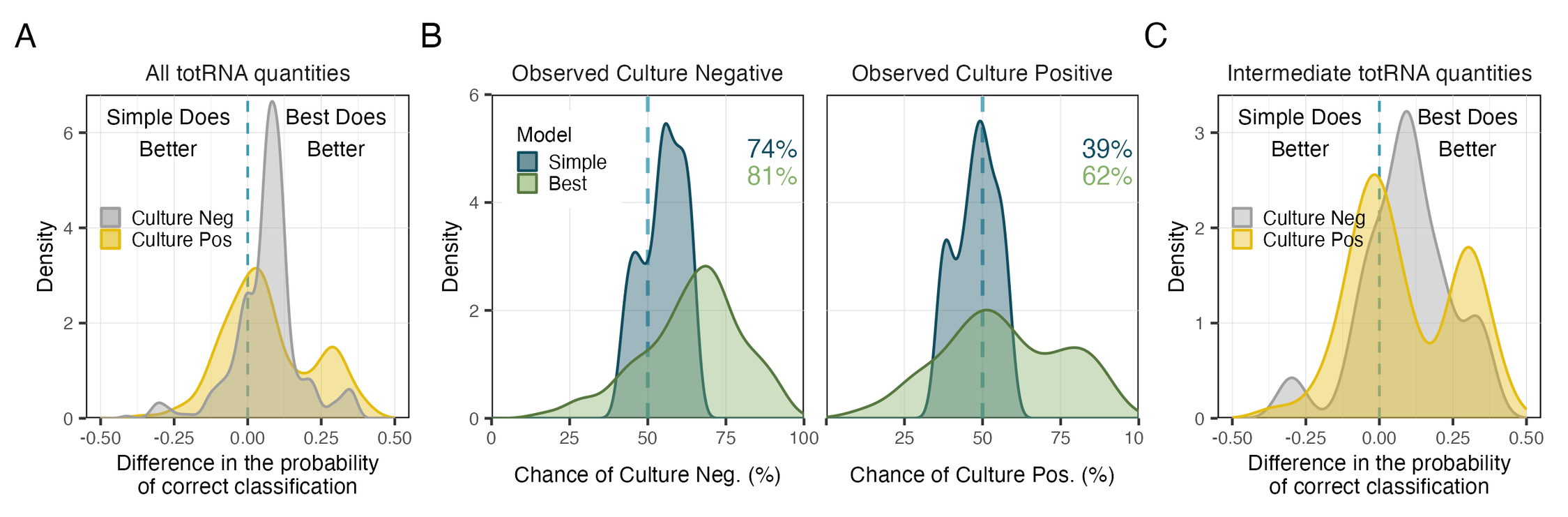

Supplement: S15 Fig — (A) Distribution of the differences between the predicted probabilities of both models for all totRNA-positive samples, stratified by whether the sample was culture positive (yellow) or negative (grey). Samples on the right side of the dashed blue line were predicted with higher confidence by the best model, while those on the left side were predicted with higher confidence by the simple model. (B) Distribution of median model-predicted chances of positive culture for intermediate totRNA-positive samples (6–8 log10 copies), stratified by model type and observed outcomes. Samples right of the dashed vertical line are correct predictions. The colored text gives the percent of samples that are correctly classified by each model. (C) As in panel A, except only for intermediate totRNA-positive samples (6–8 log10 copies). (TIF) [file ppat.1012171.s016.tif]

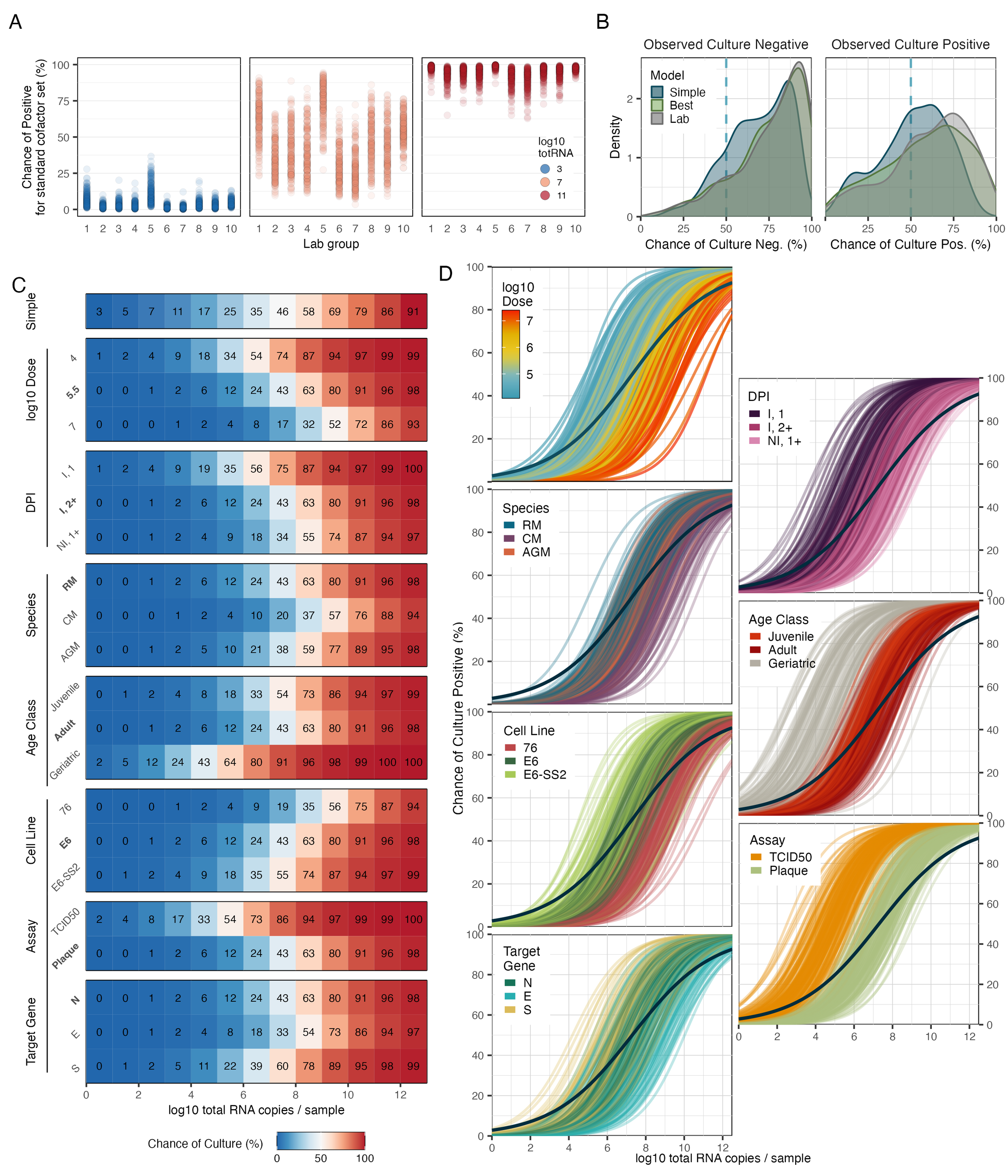

Supplement: S16 Fig — (A) The predicted chances of culture positivity for three key totRNA quantities (3 log10, blue; 7 log10, salmon; 11 log10, red), across the ten available lab groups and for the standard cofactor set. The article(s) included in each group are listed in S8 Table. Each point is one out of 200 samples generated for each lab group, with transparency to show the density of points. (B) As in Fig 5B, with additional predictions from the model including a lab effect (‘Lab’, grey). (C and D) As in Fig 5C and 5D, except showing the results from the model including a lab effect. In panels C and D, the predictions are not specific to a particular lab group (i.e., we set the lab effect term to zero to extract general patterns across all labs). (TIF) [file ppat.1012171.s017.tif]

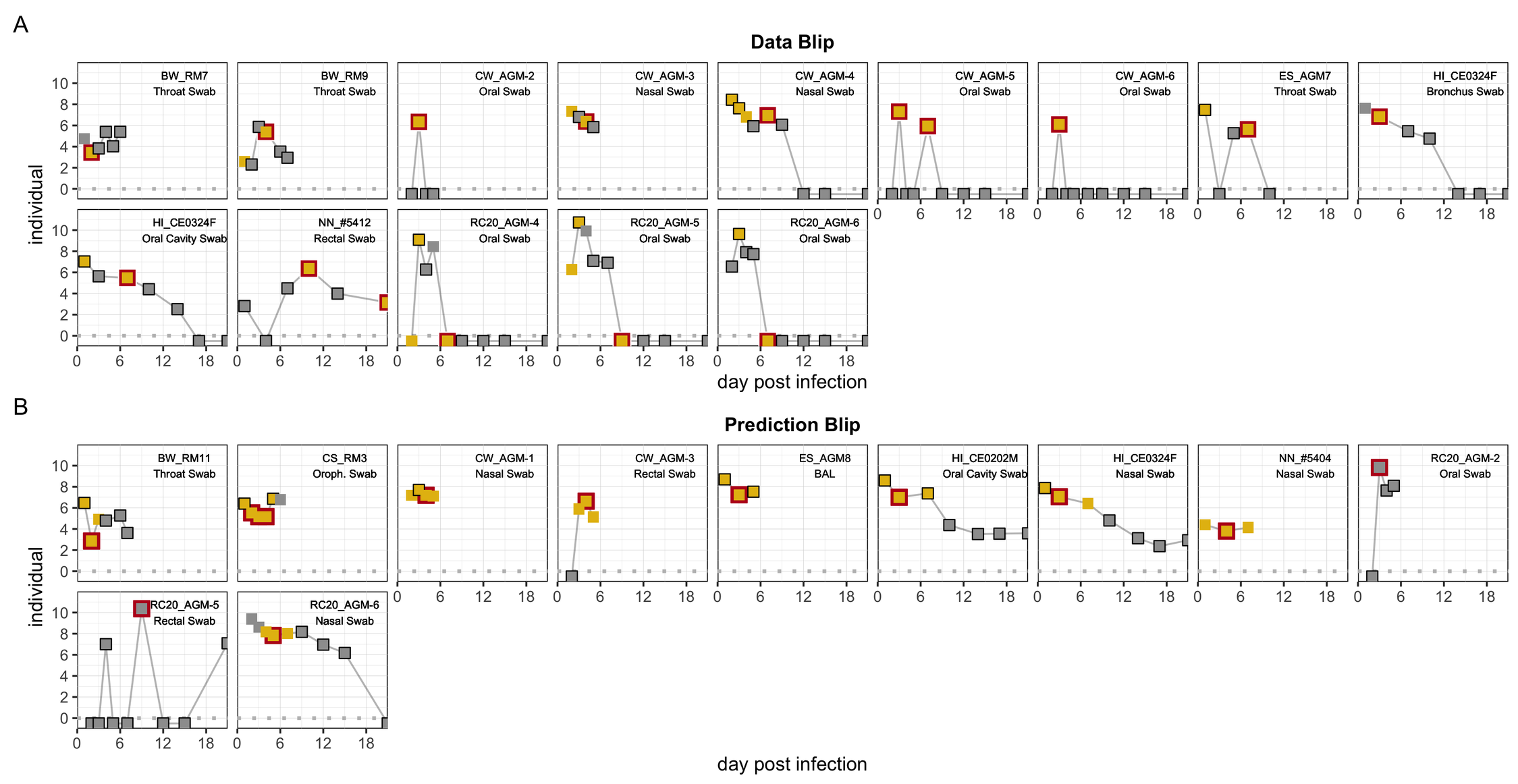

Supplement: S17 Fig — Viral load and culture trajectories for individuals with data blip (A) or prediction blip (B) error types. Panel-specific errors are indicated with red outlines. All other samples with prediction errors have no outline. Correct predictions are outlined in black. Yellow squares indicate known culture positive samples, while grey squares indicate known culture negative samples. Text in the upper right corner of each panel indicates the ID name and sample type of the individual from whom the data was derived. All totRNA-negative samples are plotted below the grey dashed line at zero. Note that individual NN_#5412 has an additional (true negative) sample available on a later day post infection, which is not shown for visual clarity. Six trajectories from one study cannot be included in this figure due to a data sharing agreement. (TIF) [file ppat.1012171.s018.tif]

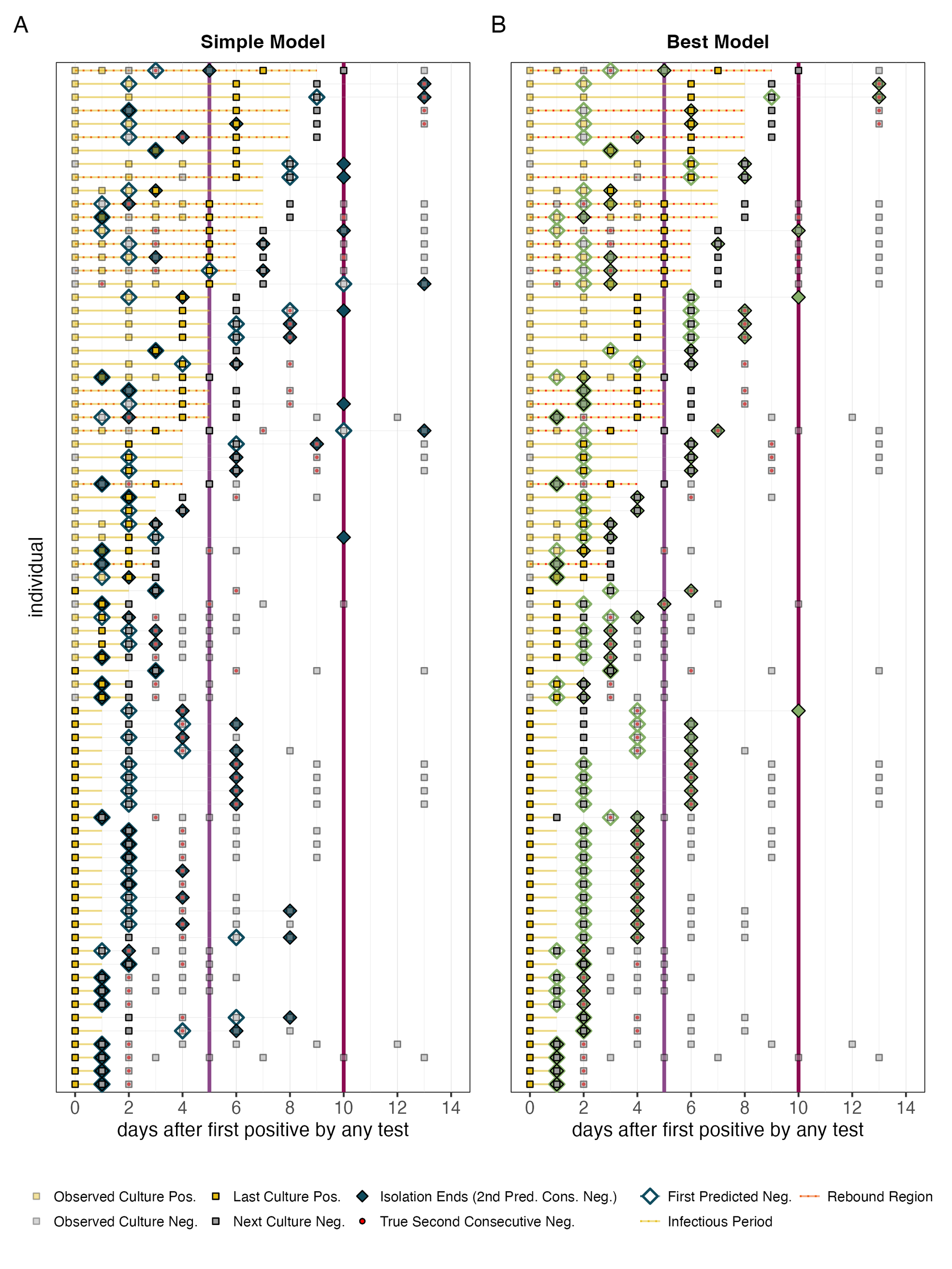

Supplement: S18 Fig — Isolation end times predicted by the simple (A) and best (B) culture models. Each row is a unique individual, and each panel displays all individuals included in the isolation analyses. The results of all samples after every individual’s first positive test (PCR or culture) are displayed, where culture positive samples are yellow and negative samples are grey. Each individual’s last culture positive and their subsequent culture negative times are plotted with more intensity for better visualization. For each individual, their isolation end time is shown with colored, filled diamonds (i.e., the time of their second consecutive predicted culture negative test). When isolation end time could not be determined by the model (i.e., the model did not predict a second consecutive negative), we conservatively set that individual’s end time to day 10. Each individual’s first predicted negative is shown by an empty diamond, and the true (observed) time of their second consecutive negative is shown with a small red point. With yellow lines, we show the time range that we consider each individual to be infectious, based on the data, which ranges from their first total RNA positive day up to the midpoint between their first culture negative test after their last observed culture positive test. For individuals with no observed negative after their last positive, we conservatively assumed their next observed negative to be day 10. With dashed red lines, we also indicate which individuals show evidence of a rebound infection (i.e., the individuals with at least one culture negative occurring between two culture positives). Finally, we use colored vertical lines to display the days on which the five- and ten-day protocols would release individuals from isolation. (TIF) [file ppat.1012171.s019.tif]

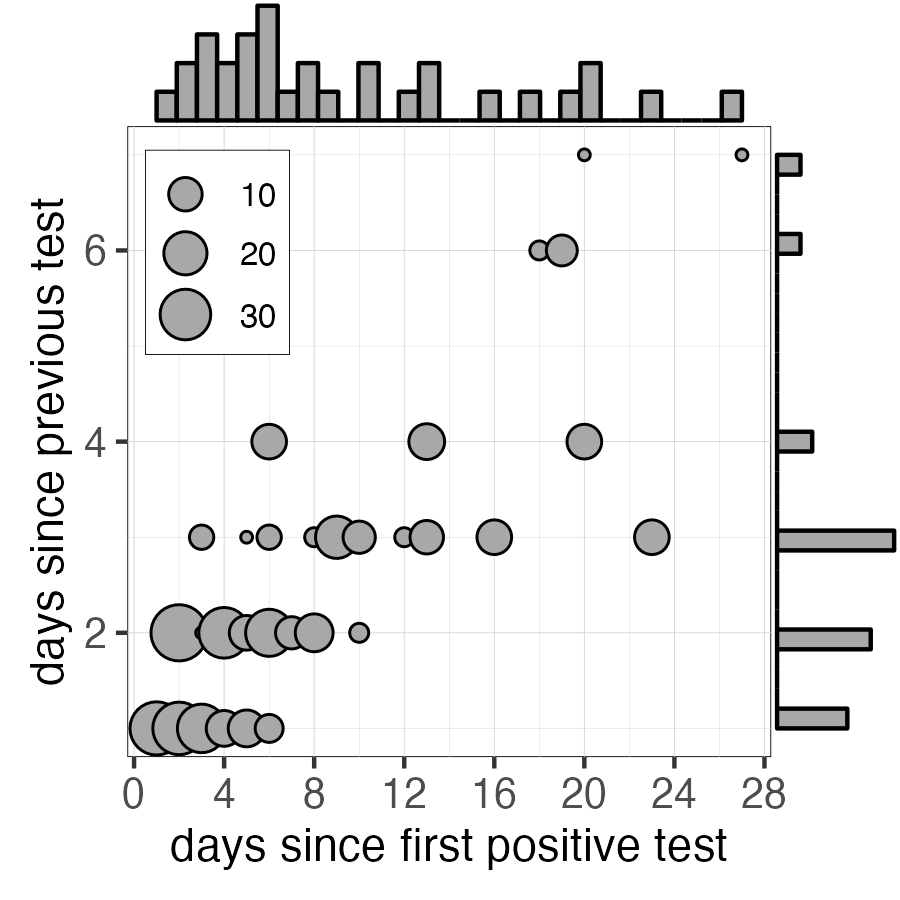

Supplement: S19 Fig — The size of the point shows the number of samples at the given coordinate. The marginal histograms show the distribution of points along each individual axis. (TIF) [file ppat.1012171.s020.tif]
