# Supplementary material for: Predicting the presence of infectious virus from PCR data: A meta-analysis of SARS-CoV-2 in non-human primates
Source: PLoS Pathog. 2024 Apr 29;20(4):e1012171. doi: 10.1371/journal.ppat.1012171 (PMC11081500; doi:10.1371/journal.ppat.1012171)
Supplement: S1 Table — Multiple rows for an individual article are included when the study involved multiple species and/or multiple exposure doses. In all columns, U indicates the detail is unknown. Sample sizes (N) are presented in the following format: number of available datapoints (number of individuals). Species abbreviations are as follows: RM, rhesus macaque; CM, cynomolgus macaque; AGM, African green monkey. Age class presents the standardized assignments according to our protocol (S1 Methods), and the abbreviations are: J, juvenile; A, adult; G, geriatric. Individuals inoculated via multiple routes are indicated by exposure routes joined by commas, where the abbreviations are: AE, aerosol; IT, intratracheal; IN, intranasal; IG, intragastric; OC, ocular; OR, oral. Exposure dose is presented as log10 plaque forming units, and an adjoining * indicates the dose was originally reported as TCID50, so those values were converted using the standard method described in the S1 Methods. NI indicates non-invasive sample types (i.e., swabs, biofluids, BAL), while I indicates invasive tissue samples obtained at necropsy. Sample location distinguishes between the following systems: URT, upper respiratory tract; LRT, lower respiratory tract; GI, gastrointestinal tract; and Other, all other locations. Sample time presents the days post infection with available samples according to our DPI predictor, where 1: 1 dpi, inoculated tissues, 2: 2+ dpi, inoculated tissues, 3: any dpi, non-inoculated tissue (further categorization information is in S9 Table). PCR target genes are stratified by total RNA (totRNA) and sgRNA. The level of the target gene predictor for the sgRNA model follows the sgRNA gene in parentheses: (1) totRNA-high/sgRNA-high, (2) totRNA-low/sgRNA-high, (3) totRNA-high/sgRNA-low, and (4) totRNA-low/sgRNA-low. The cell lines used for culture are indicated when available, with SS2 as an abbreviation for TMPRSS2. An adjoining † indicates the use of a TCID50 assay, while no symbol indicat [file ppat.1012171.s021.docx]

| Article (ref.) | N | Species | Sex | Age  class | Exposure  route | Exposure  dose | Viral  isolate | Sample  type | Sample  time | Sample  location | PCR target gene | | Culture  cell line |
| --- | --- | --- | --- | --- | --- | --- | --- | --- | --- | --- | --- | --- | --- |
|  |  |  |  |  |  |  |  |  |  |  | ***totRNA*** | ***sgRNA*** |  |
| Baum et al. 2020 (1) | 43 (4) | RM | F, M | U | IT, IN | 6.02 | USA/WA1/2020 | NI | 1, 2, 3 | URT, Other | N | E (3) | -- |
|  | 48 (6) | RM | F, M | A | IT, IN | 5.04 | USA/WA1/2020 | NI | 1, 2 | URT, LRT | N | E (3) | -- |
| Chandrashekar et al. 2020  (2) | 12 (3) | RM | U | A | IT, IN | 6.04 | USA/WA1/2020 | NI | 1, 2 | URT | N | E (3) | -- |
|  | 12 (3) | RM | U | A | IT, IN | 5.04 | USA/WA1/2020 | NI | 1, 2 | URT | N | E (3) | -- |
|  | 12 (3) | RM | U | A | IT, IN | 4.04 | USA/WA1/2020 | NI | 1, 2 | URT | N | E (3) | -- |
| Corbett et al. 2020 (3) | 50 (8) | RM | F, M | J, A | IT, IN | 5.88 | USA/WA1/2020 | NI | 1, 2 | URT, LRT | N | E (3) | -- |
| Cross et al. 2020 (4) | 124 (6) | AGM | F | A | IN | 6.45 | ITA/INMI1/2020 | NI | 2, 3 | URT, LRT, GI, Other | N | -- | Vero E6 |
| Dagotto et al. 2021 (5) | 16 (4) | RM | U | A | IT, IN | 4.04 | USA/WA1/2020 | NI | 1, 2 | LRT | N, E | E (3, 4) | -- |
| Deng et al. 2020 (6) | 7 (1) | RM | M | J | IT | 5.85* | CHN/WH-09/2020 | I | 3 | LRT | E | -- | Vero E6† |
|  | 7 (1) | RM | M | J | OC | 5.85* | CHN/WH-09/2020 | I | 3 | LRT | E | -- | Vero E6† |
| Gabitzsch et al. 2021 (7) | 24 (2) | RM | F, M | J | IT, IN | 5.85* | USA/WA1/2020 | NI | 1, 2 | URT, LRT | N | E (3) | -- |
| Ishigaki et al. 2021 (8) | 144 (3) | CM | F, M | A | IT, IN, OR, OC | 6.19* | JPN/WK-521/2020 | NI | 1, 2, 3 | URT, LRT, GI, Other | N | -- | Vero E6† |
| Jiao et al. 2021 (9) | 16 (3) | RM | M | U | IN | 7 | CHN/U | I | 3 | GI | N | -- | Vero E6† |
|  | 14 (3) | RM | M | U | IG | 7 | CHN/U | I | 1, 2, 3 | GI | N | -- | Vero E6† |
| Johnston et al. 2020 (10) | 60 (4) | RM | F, M | A | AE | 4.46 | USA/WA1/2020 | NI | 2, 3 | URT, GI | N | -- | Vero 76 |
|  | 45 (3) | AGM | F, M | A | AE | 4.58 | USA/WA1/2020 | NI | 2, 3 | URT, GI | N | -- | Vero 76 |
|  | 60 (4) | CM | F, M | J, A | AE | 4.69 | USA/WA1/2020 | NI | 2, 3 | URT, GI | N | -- | Vero 76 |
| Jones et al. 2021 (11) | 44 (4) | RM | F | A | IT, IN | 5.04 | USA/WA1/2020 | I, NI | 1, 2, 3 | URT, LRT | N | E (3) | -- |
| Kobiyama et al. 2021 (12) | 26 (2) | CM | F | A | IT, IN, OR, OC | 7.3 | U/U | NI | 1, 2 | URT, LRT, Other | N | -- | Vero E6-SS2† |
| Li et al. 2021 (13) | 148 (16) | CM | F, M | J, A | IT, IN | 5 | USA/WA1/2020 | NI | 2 | URT, LRT | E | E, N (4, 2) | -- |
| Munster et al. 2020 (14) | 53 (8) | RM | F, M | J, A | IT, IN, OR, OC | 6.26* | USA/WA1/2020 | I, NI | 1, 2, 3 | LRT, GI, Other | E | ORF7 (4) | Vero E6 |
| Nagata et al. 2021 (15) | 165 (6) | CM | F | A | IT, IN, OC | 7.42* | JPN/WK-521/2020 | I, NI | 1, 2, 3 | URT, LRT, GI, Other | N | N (1) | Vero E6-SS2† |
| Patel et al. 2021 (16) | 65 (5) | RM | F, M | J | IT, IN | 4.04 | USA/WA1/2020 | NI | 1, 2 | URT, LRT | N | E (3) | -- |
| Salguero et al. 2021 (17) | 63 (6) | RM | F, M | J | IT, IN | 6.7 | AUS/VIC01/2020 | I, NI | 1, 2, 3 | URT, LRT, Other | N | E (3) | Vero E6 |
|  | 58 (6) | CM | F, M | J | IT, IN | 6.7 | AUS/VIC01/2020 | I, NI | 1, 2, 3 | URT, LRT, Other | N | E (3) | Vero E6 |
| Shan et al. 2020 (18) | 108 (6) | RM | F, M | A | IT | 6.69* | CHN/WIV04/2019 | NI | 3 | URT, GI | S | -- | Vero E6† |
| Singh et al. 2020 (19) | 108 (16) | RM | F, M | G, J | IT, IN, OC | 6.02 | USA/WA1/2020 | I | 3 | LRT | N | E (3) | Vero E6 |
| Speranza et al. 2020 (20) | 194 (10) | AGM | F, M | A | IT, IN, OR, OC | 6.26* | USA/WA1/2020 | I, NI | 1, 2, 3 | URT, LRT, GI | E | E (4) | Vero E6 |
| van Doremalen et al. 2020 (21) | 72 (6) | RM | U | J | IT, IN, OR, OC | 6.26* | USA/WA1/2020 | I, NI | 1, 2, 3 | URT, LRT, GI, Other | -- | E | Vero E6† |
| Williamson et al. 2020 (22) | 135 (6) | RM | F, M | J | IT, IN, OR, OC | 6.26* | USA/WA1/2020 | I, NI | 1, 2, 3 | URT, LRT, GI | E | -- | Vero E6† |
| Woolsey et al. 2020 (23) | 132 (6) | AGM | F, M | A | IT, IN | 5.66 | ITA/INMI1/2020 | NI | 2, 3 | URT, LRT, GI, Other | N | -- | Vero E6 |
| Yu et al. 2020 (24) | 102 (10) | RM | U | A | IT, IN | 4.04 | U/U | NI | 1, 2 | URT, LRT | N | E (3) | -- |

**References**

1. Baum A, Ajithdoss D, Copin R, Zhou A, Lanza K, Negron N, et al. REGN-COV2 antibodies prevent and treat SARS-CoV-2 infection in rhesus macaques and hamsters. Science. 2020 Nov 27;370(6520):1110–5.
2. Chandrashekar A, Liu J, Martinot AJ, McMahan K, Mercado NB, Peter L, et al. SARS-CoV-2 infection protects against rechallenge in rhesus macaques. Science. 2020 May;eabc4776–eabc4776.
3. Corbett KS, Flynn B, Foulds KE, Francica JR, Boyoglu-Barnum S, Werner AP, et al. Evaluation of the mRNA-1273 Vaccine against SARS-CoV-2 in Nonhuman Primates. N Engl J Med. 2020 Jul;NEJMoa2024671–NEJMoa2024671.
4. Cross RW, Agans KN, Prasad AN, Borisevich V, Woolsey C, Deer DJ, et al. Intranasal exposure of African green monkeys to SARS-CoV-2 results in acute phase pneumonia with shedding and lung injury still present in the early convalescence phase. Virol J. 2020 Dec;17(1):125–125.
5. Dagotto G, Mercado NB, Martinez DR, Hou YJ, Nkolola JP, Carnahan RH, et al. Comparison of Subgenomic and Total RNA in SARS-CoV-2-Challenged Rhesus Macaques. J Virol. 2021 Mar 25;95(8).
6. Deng W, Bao L, Gao H, Xiang Z, Qu Y, Song Z, et al. Ocular conjunctival inoculation of SARS-CoV-2 can cause mild COVID-19 in rhesus macaques. Nat Commun. 2020 Dec;11(1):4400–4400.
7. Gabitzsch E, Safrit JT, Verma M, Rice A, Sieling P, Zakin L, et al. Dual-Antigen COVID-19 Vaccine Subcutaneous Prime Delivery With Oral Boosts Protects NHP Against SARS-CoV-2 Challenge. Front Immunol. 2021;12.
8. Ishigaki H, Nakayama M, Kitagawa Y, Nguyen CT, Hayashi K, Shiohara M, et al. Neutralizing antibody-dependent and -independent immune responses against SARS-CoV-2 in cynomolgus macaques. Virology. 2021 Feb 1;554:97–105.
9. Jiao L, Li H, Xu J, Yang M, Ma C, Li J, et al. The Gastrointestinal Tract Is an Alternative Route for SARS-CoV-2 Infection in a Nonhuman Primate Model. Gastroenterology. 2021 Apr 1;160(5):1647–61.
10. Johnston SC, Ricks KM, Jay A, Raymond JL, Rossi F, Zeng X, et al. Development of a coronavirus disease 2019 nonhuman primate model using airborne exposure. PLOS ONE. 2021 Feb 2;16(2):e0246366.
11. Jones BE, Brown-Augsburger PL, Corbett KS, Westendorf K, Davies J, Cujec TP, et al. The neutralizing antibody, LY-CoV555, protects against SARS-CoV-2 infection in nonhuman primates. Sci Transl Med. 2021 May 12;13(593):eabf1906.
12. Kobiyama K, Imai M, Jounai N, Nakayama M, Hioki K, Iwatsuki-Horimoto K, et al. Optimization of an LNP-mRNA vaccine candidate targeting SARS-CoV-2 receptor-binding domain. bioRxiv. 2021. Available from: <https://www.biorxiv.org/content/10.1101/2021.03.04.433852v1>
13. Li D, Edwards RJ, Manne K, Martinez DR, Schäfer A, Alam SM, et al. In vitro and in vivo functions of SARS-CoV-2 infection-enhancing and neutralizing antibodies. Cell. 2021 Aug 5;184(16):4203-4219.e32.
14. Munster VJ, Feldmann F, Williamson BN, van Doremalen N, Pérez-Pérez L, Schulz J, et al. Respiratory disease in rhesus macaques inoculated with SARS-CoV-2. Nature. 2020 Sep;585(7824):268–72.
15. Nagata N, Iwata-Yoshikawa N, Sano K, Ainai A, Shiwa N, Shirakura M, et al. The peripheral T cell population is associated with pneumonia severity in cynomolgus monkeys experimentally infected with severe acute respiratory syndrome coronavirus 2. bioRxiv. 2021. Available from: <https://www.biorxiv.org/content/10.1101/2021.01.07.425698v1>
16. Patel A, Walters JN, Reuschel EL, Schultheis K, Parzych E, Gary EN, et al. Intradermal-delivered DNA vaccine induces durable immunity mediating a reduction in viral load in a rhesus macaque SARS-CoV-2 challenge model. Cell Rep Med. 2021 Oct 19;2(10):100420.
17. Salguero FJ, White AD, Slack GS, Fotheringham SA, Bewley KR, Gooch KE, et al. Comparison of rhesus and cynomolgus macaques as an infection model for COVID-19. Nat Commun. 2021 Feb 24;12(1):1260.
18. Shan C, Yao YF, Yang XL, Zhou YW, Gao G, Peng Y, et al. Infection with novel coronavirus (SARS-CoV-2) causes pneumonia in Rhesus macaques. Cell Res. 2020 Jul;1–8.
19. Singh DK, Singh B, Ganatra SR, Gazi M, Cole J, Thippeshappa R, et al. Responses to acute infection with SARS-CoV-2 in the lungs of rhesus macaques, baboons and marmosets. Nat Microbiol. 2021 Jan;6(1):73–86.
20. Speranza E, Williamson BN, Feldmann F, Sturdevant GL, Pérez LP, Meade-White K, et al. Single-cell RNA sequencing reveals SARS-CoV-2 infection dynamics in lungs of African green monkeys. Sci Transl Med. 2021 Jan 27;13(578).
21. van Doremalen N, Lambe T, Spencer A, Belij-Rammerstorfer S, Purushotham JN, Port JR, et al. ChAdOx1 nCoV-19 vaccine prevents SARS-CoV-2 pneumonia in rhesus macaques. Nature. 2020 Jul;1–8.
22. Williamson BN, Feldmann F, Schwarz B, Meade-White K, Porter DP, Schulz J, et al. Clinical benefit of remdesivir in rhesus macaques infected with SARS-CoV-2. Nature. 2020 Jun;1–7.
23. Woolsey C, Borisevich V, Prasad AN, Agans KN, Deer DJ, Dobias NS, et al. Establishment of an African green monkey model for COVID-19 and protection against re-infection. Nat Immunol. 2021 Jan;22(1):86–98.
24. Yu J, Tostanoski LH, Peter L, Mercado NB, McMahan K, Mahrokhian SH, et al. DNA vaccine protection against SARS-CoV-2 in rhesus macaques. Science. 2020 May;eabc6284–eabc6284.
