# Supplementary material for: Predicting the presence of infectious virus from PCR data: A meta-analysis of SARS-CoV-2 in non-human primates
Source: PLoS Pathog. 2024 Apr 29;20(4):e1012171. doi: 10.1371/journal.ppat.1012171 (PMC11081500; doi:10.1371/journal.ppat.1012171)
Supplement: S3 Table — Models are ordered by increasing number of predictors, with the simplest (f1), best (f5.1), and full (f8.1) models noted in bold. We report expected log pointwise predictive density (ELPD) generated by 10-fold cross validation (cross-validation columns), where larger ELPD indicates better performance. The best logistic model was run in tandem with all tested linear components, so the ELPD reported here reflects the sum of the ELPD for the best logistic and the considered linear components. ELPD difference indicates the difference between ELPDs of the given model and the model with the largest ELPD (in this case model l5.1, the ‘best model’). The PSIS-LOO approximation columns present statistics generated by running Pareto-Smoothed Importance Sampling approximate leave-one-out cross validation, including ELPD and ELPD difference. Standard error (SE) is shown in parentheses following all relevant statistics. We also used multiple metrics to assess model predictions, which are all stratified by performance on training versus test data sets and were generated by 10-fold cross validation. MAE is the median difference between the observed value and the posterior predictive median (i.e., median absolute error around the median) for all samples with sgRNA above the LOD, and this metric was also scaled by one standard deviation (Scaled). ‘% within 50% PI’ and ‘% within 95% PI’ columns indicate the percent of sgRNA positive samples where the true, observed value fell within the sample-specific 50% and 95% prediction intervals, respectively. Note that all models included total RNA as a predictor, even though it is not specified in the predictor column. (DOCX) [file ppat.1012171.s023.docx]

|  |  | *Cross-validation* | | *PSIS-LOO Approximation* | | *Prediction* | | | | | | |
| --- | --- | --- | --- | --- | --- | --- | --- | --- | --- | --- | --- | --- |
| **Model** | **Predictors** | **ELPD**  **Difference**  **(SE)** | **ELPD**  **(SE)** | **ELPD**  **Difference**  **(SE)** | **ELPD**  **(SE)** | **MAE**  **(scaled)** | | **%**  **within**  **50% PI** | | | **%**  **within**  **95% PI** | |
|  |  |  |  |  |  | **train** | **test** | **train** | **test** | **train** | | **test** |
| **f1** |  | **-186.24 (17.05)** | **-1017.32 (29.41)** | **-196.49 (21.07)** | **-1017.12 (29.35)** | **0.58 (0.7)** | **0.58 (0.7)** | **47.5** | **48** | **94.9** | | **94.8** |
| f2.1 | DOSE | -114.04 (11.7) | -945.12 (32.24) | -111.91 (15.27) | -932.54 (30.49) | 0.6 (0.65) | 0.6 (0.65) | 46.9 | 46.6 | 95.9 | | 95.6 |
| f2.2 | ST | -98.38 (10.49) | -929.46 (32.38) | -95.87 (13.7) | -916.5 (30.74) | 0.56 (0.63) | 0.56 (0.63) | 49.9 | 50.3 | 96.5 | | 96 |
| f2.3 | SP | -109.47 (11.8) | -940.55 (32.74) | -106.94 (15.43) | -927.56 (31.08) | 0.56 (0.61) | 0.57 (0.62) | 50.7 | 49.4 | 96.2 | | 95.8 |
| f2.4 | AGE | -107.02 (12.6) | -938.1 (32.65) | -104.67 (16.34) | -925.29 (30.91) | 0.57 (0.62) | 0.57 (0.64) | 50.7 | 49.6 | 96.9 | | 96.5 |
| f2.5 | SEX | -120.9 (12.58) | -951.98 (32.98) | -129.78 (17.11) | -950.41 (32.6) | 0.56 (0.62) | 0.58 (0.64) | 50.6 | 48.5 | 96.2 | | 95.8 |
| f2.6 | DPI | -82.23 (9.7) | -913.31 (31.99) | -79.37 (12.66) | -900 (30.31) | 0.56 (0.65) | 0.55 (0.64) | 47.9 | 47.5 | 96.1 | | 96 |
| f2.7 | TG | -94.6 (11.02) | -925.68 (33.96) | -93.29 (14.53) | -913.91 (32.33) | 0.55 (0.61) | 0.57 (0.63) | 50.7 | 50.4 | 97.8 | | 97.7 |
| f3.1 | DPI + DOSE | -83.66 (9.65) | -914.74 (32.05) | -81.44 (12.63) | -902.06 (30.39) | 0.56 (0.65) | 0.56 (0.65) | 48 | 48.5 | 96.4 | | 96 |
| f3.2 | DPI + ST | -82.78 (9.59) | -913.86 (32.07) | -80.27 (12.56) | -900.9 (30.46) | 0.55 (0.65) | 0.55 (0.65) | 47.6 | 47.8 | 96.1 | | 96 |
| f3.3 | DPI + SP | -78.12 (9.53) | -909.2 (32.18) | -76.32 (12.54) | -896.95 (30.58) | 0.55 (0.65) | 0.55 (0.65) | 48.1 | 48.5 | 96 | | 95.8 |
| f3.4 | DPI + AGE | -74.78 (10.35) | -905.86 (32.11) | -72.99 (13.5) | -893.62 (30.54) | 0.54 (0.64) | 0.54 (0.65) | 49.6 | 48.9 | 96.3 | | 96.2 |
| f3.5 | DPI + SEX | -87.71 (10.23) | -918.79 (32.22) | -91.38 (13.37) | -912 (30.95) | 0.54 (0.64) | 0.57 (0.67) | 48.9 | 46.6 | 95.9 | | 95.5 |
| f3.6 | DPI + TG | -48.36 (8.3) | -879.44 (34.01) | -46.14 (10.88) | -866.77 (32.38) | 0.51 (0.61) | 0.52 (0.6) | 50.8 | 51 | 97.7 | | 97.7 |
| f4.1 | DPI + TG + DOSE | -40.6 (8.79) | -871.68 (35.31) | -38.35 (11.32) | -858.98 (33.91) | 0.49 (0.54) | 0.49 (0.53) | 52.1 | 52.2 | 96.6 | | 96.7 |
| f4.2 | DPI + TG + ST | -44.28 (7.39) | -875.36 (34.38) | -42.03 (9.66) | -862.65 (32.86) | 0.51 (0.6) | 0.51 (0.59) | 51.3 | 51.7 | 97.7 | | 97.6 |
| f4.3 | DPI + TG + SP | -2.82 (3.66) | -833.9 (34.41) | -1.33 (4.81) | -821.95 (33.02) | 0.46 (0.57) | 0.47 (0.57) | 53.1 | 52.7 | 96.5 | | 97 |
| f4.4 | DPI + TG + AGE | -36.03 (7.45) | -867.11 (33.05) | -34.98 (9.47) | -855.6 (31.27) | 0.48 (0.56) | 0.49 (0.59) | 53.8 | 52.9 | 97 | | 96.7 |
| f4.5 | DPI + TG + SEX | -52.59 (8.54) | -883.67 (34.03) | -53.68 (11.25) | -874.3 (32.66) | 0.5 (0.6) | 0.52 (0.61) | 51.6 | 50.8 | 97.6 | | 97.4 |
| **f5.1** | **DPI + TG + SP + DOSE** | **0 (0)** | **-831.08 (34.5)** | **0 (0)** | **-820.63 (33.24)** | **0.43 (0.53)** | **0.44 (0.54)** | **56** | **55** | **97** | | **96.9** |
| f5.2 | DPI + TG + SP + ST | -3.41 (3.65) | -834.49 (34.35) | -1.83 (4.82) | -822.45 (32.99) | 0.46 (0.57) | 0.48 (0.58) | 52.8 | 52.7 | 96.4 | | 96.7 |
| f5.3 | DPI + TG + SP + AGE | -4.88 (3.34) | -835.96 (34.24) | -4.13 (4.51) | -824.76 (32.79) | 0.46 (0.57) | 0.48 (0.58) | 53.5 | 53.1 | 96.6 | | 96.7 |
| f5.4 | DPI + TG + SP + SEX | -4.64 (3.6) | -835.72 (34.36) | -7.22 (4.65) | -827.85 (33.13) | 0.45 (0.57) | 0.48 (0.59) | 53.4 | 52.4 | 96.5 | | 96 |
| f6.1 | DPI + TG + SP + DOSE + ST | -1.11 (0.32) | -832.19 (34.53) | -0.68 (0.31) | -821.3 (33.14) | 0.43 (0.53) | 0.44 (0.54) | 56 | 54.5 | 97 | | 96.7 |
| f6.2 | DPI + TG + SP + DOSE + AGE | -1.51 (1.17) | -832.59 (34.4) | -1.01 (1.9) | -821.64 (32.86) | 0.43 (0.53) | 0.43 (0.53) | 56.5 | 55.3 | 97 | | 96.7 |
| f6.3 | DPI + TG + SP + DOSE + SEX | -1.5 (0.69) | -832.58 (34.48) | -5.76 (0.85) | -826.39 (33.37) | 0.43 (0.53) | 0.44 (0.54) | 56.6 | 54.5 | 96.9 | | 96.7 |
| f7.1 | DPI + TG + SP + DOSE + ST + AGE | -2.64 (1.22) | -833.72 (34.4) | -2.56 (1.85) | -823.19 (33.01) | 0.43 (0.52) | 0.43 (0.53) | 56.4 | 55.1 | 97 | | 96.7 |
| f7.2 | DPI + TG + SP + DOSE + ST + SEX | -2.72 (0.76) | -833.8 (34.54) | -6.76 (0.88) | -827.39 (33.4) | 0.43 (0.53) | 0.44 (0.54) | 56.4 | 54.1 | 96.9 | | 96.9 |
| **f8.1** | **DPI + TG + SP + DOSE + ST + AGE + SEX** | **-4.44 (2.39)** | **-835.52 (34.2)** | **-9.76 (4.12)** | **-830.38 (33.19)** | **0.43 (0.53)** | **0.45 (0.56)** | **57.2** | **54.5** | **96.9** | | **96.7** |
