# Supplementary material for: Predicting the presence of infectious virus from PCR data: A meta-analysis of SARS-CoV-2 in non-human primates
Source: PLoS Pathog. 2024 Apr 29;20(4):e1012171. doi: 10.1371/journal.ppat.1012171 (PMC11081500; doi:10.1371/journal.ppat.1012171)
Supplement: S5 Table — Models are ordered by increasing number of predictors, with the simplest (c1) and best/full (c10.1) models noted in bold. We report expected log pointwise predictive density (ELPD) generated by 10-fold cross validation (cross-validation columns), where larger ELPD indicates better performance. ELPD difference indicates the difference between ELPDs of the given model and the model with the largest ELPD (in this case model c10.1, our ‘best model’). The PSIS-LOO approximation columns present statistics generated by running Pareto-Smoothed Importance Sampling approximate leave-one-out cross validation, including ELPD and ELPD difference. The prediction column indicates the percent of samples (stratified by training and test sets) for which posterior predictions generated by 10-fold cross validation correctly classified them as below or above the limit of detection (i.e., where the per-sample posterior predictive distributions exhibited at least a probability of 0.5 for the true, observed classification). MCC is the Matthews correlation coefficient. Standard error (SE) is shown in parentheses following all relevant statistics. (DOCX) [file ppat.1012171.s025.docx]

|  |  | *Cross-validation* | | *PSIS-LOO Approximation* | | *Prediction* | | |
| --- | --- | --- | --- | --- | --- | --- | --- | --- |
| **Model** | **Predictors** | **ELPD**  **Difference**  **(SE)** | **ELPD**  **(SE)** | **ELPD**  **Difference**  **(SE)** | **ELPD**  **(SE)** | **MCC** | **% correctly**  **predicted** | |
|  |  |  |  |  |  |  | **train** | **test** |
| **c1** | **SG** | **-48.77 (17.87)** | **-350.19 (7.87)** | **-47.12 (9.22)** | **-350.38 (7.97)** | **0.43** | **80.8** | **80.8** |
| c2.1 | SG + CELL | -46.15 (17.66) | -347.58 (8.24) | -45.15 (9.11) | -348.41 (8.36) | 0.46 | 81.5 | 81.6 |
| c2.2 | SG + ASSAY | -43.94 (17.39) | -345.37 (8.14) | -42.73 (8.97) | -345.98 (8.26) | 0.44 | 81.1 | 80.9 |
| c2.3 | SG + DOSE | -49.75 (18.01) | -351.18 (8.13) | -48.24 (9.27) | -351.5 (8.2) | 0.42 | 80.6 | 80.5 |
| c2.4 | SG + ST | -44.12 (16.49) | -345.55 (8.51) | -42.39 (8.48) | -345.64 (8.65) | 0.47 | 82.1 | 81.9 |
| c2.5 | SG + SP | -47.13 (16.95) | -348.56 (8.21) | -45.76 (8.76) | -349.02 (8.31) | 0.43 | 81.1 | 80.9 |
| c2.6 | SG + AGE | -47.52 (17.64) | -348.94 (8.27) | -46.21 (9.12) | -349.47 (8.34) | 0.44 | 80.9 | 80.9 |
| c2.7 | SG + SEX | -48.93 (17.77) | -350.35 (8.13) | -47.77 (9.19) | -351.03 (8.24) | 0.44 | 81.3 | 81.1 |
| c2.8 | SG + DPI | -16.3 (10.84) | -317.73 (10.23) | -14.63 (5.69) | -317.89 (10.32) | 0.52 | 83.5 | 83.4 |
| c2.9 | SG + TG | -49.06 (17.9) | -350.49 (8.11) | -47.51 (9.23) | -350.77 (8.19) | 0.43 | 80.7 | 80.8 |
| c3.1 | SG + DPI + CELL | -15.02 (10.4) | -316.44 (10.4) | -13.52 (5.47) | -316.78 (10.51) | 0.54 | 84 | 84 |
| c3.2 | SG + DPI + ASSAY | -13.09 (9.9) | -314.52 (10.36) | -11.79 (5.18) | -315.05 (10.47) | 0.52 | 83.8 | 83.6 |
| c3.3 | SG + DPI + DOSE | -17.31 (11.11) | -318.74 (10.42) | -15.68 (5.81) | -318.93 (10.47) | 0.51 | 83.6 | 83.3 |
| c3.4 | SG + DPI + ST | -16.67 (10.63) | -318.1 (10.31) | -15.35 (5.58) | -318.61 (10.4) | 0.52 | 83.5 | 83.5 |
| c3.5 | SG + DPI + SP | -17.26 (10.42) | -318.68 (10.34) | -15.78 (5.46) | -319.04 (10.46) | 0.51 | 83.6 | 83 |
| c3.6 | SG + DPI + AGE | -16.06 (10.62) | -317.49 (10.56) | -14.41 (5.57) | -317.67 (10.6) | 0.52 | 83.4 | 83.6 |
| c3.7 | SG + DPI + SEX | -16.15 (10.48) | -317.57 (10.45) | -14.71 (5.53) | -317.97 (10.51) | 0.53 | 83.9 | 83.7 |
| c3.8 | SG + DPI + TG | -15.41 (10.6) | -316.83 (10.55) | -14 (5.57) | -317.26 (10.63) | 0.52 | 83.8 | 83.5 |
| c4.1 | SG + DPI + ASSAY + CELL | -13.64 (9.66) | -315.06 (10.51) | -12.25 (5.08) | -315.5 (10.63) | 0.52 | 84 | 83.6 |
| c4.2 | SG + DPI + ASSAY + DOSE | -14.07 (10.26) | -315.49 (10.75) | -12.81 (5.33) | -316.07 (10.82) | 0.53 | 83.8 | 83.7 |
| c4.3 | SG + DPI + ASSAY + ST | -10.94 (8.41) | -312.36 (10.56) | -9.72 (4.42) | -312.98 (10.7) | 0.53 | 84.4 | 83.8 |
| c4.4 | SG + DPI + ASSAY + SP | -14.16 (9.73) | -315.59 (10.53) | -13.13 (5.08) | -316.39 (10.68) | 0.52 | 83.9 | 83.5 |
| c4.5 | SG + DPI + ASSAY + AGE | -10.83 (8.96) | -312.26 (10.81) | -9.42 (4.68) | -312.67 (10.9) | 0.52 | 84.3 | 83.7 |
| c4.6 | SG + DPI + ASSAY + SEX | -13.32 (9.61) | -314.74 (10.58) | -12.4 (5.06) | -315.66 (10.68) | 0.52 | 83.9 | 83.4 |
| c4.7 | SG + DPI + ASSAY + TG | -12.51 (9.7) | -313.93 (10.66) | -11.52 (5.09) | -314.78 (10.77) | 0.53 | 83.9 | 83.8 |
| c5.1 | SG + DPI + ASSAY + AGE + CELL | -11.65 (8.76) | -313.07 (10.92) | -10.11 (4.62) | -313.36 (11) | 0.52 | 84.2 | 83.7 |
| c5.2 | SG + DPI + ASSAY + AGE + DOSE | -11.86 (9.18) | -313.28 (11.06) | -10.32 (4.78) | -313.58 (11.1) | 0.52 | 84.1 | 83.5 |
| c5.3 | SG + DPI + ASSAY + AGE + ST | -9.45 (7.96) | -310.87 (11.01) | -8.18 (4.19) | -311.43 (11.13) | 0.54 | 84.3 | 84.2 |
| c5.4 | SG + DPI + ASSAY + AGE + SP | -10.83 (8.15) | -312.25 (10.94) | -9.82 (4.29) | -313.08 (11.07) | 0.53 | 84.2 | 83.8 |
| c5.5 | SG + DPI + ASSAY + AGE + SEX | -10.02 (8.18) | -311.44 (10.99) | -9.01 (4.29) | -312.27 (11.07) | 0.52 | 84.5 | 83.7 |
| c5.6 | SG + DPI + ASSAY + AGE + TG | -10.65 (8.79) | -312.07 (11.01) | -9.56 (4.62) | -312.82 (11.13) | 0.54 | 84.3 | 84.1 |
| c6.1 | SG + DPI + ASSAY + AGE + ST + CELL | -10.58 (7.89) | -312.01 (11.14) | -9.22 (4.17) | -312.48 (11.25) | 0.53 | 84.2 | 83.8 |
| c6.2 | SG + DPI + ASSAY + AGE + ST + DOSE | -10.03 (7.98) | -311.46 (11.3) | -8.65 (4.17) | -311.91 (11.39) | 0.54 | 84.6 | 84.2 |
| c6.3 | SG + DPI + ASSAY + AGE + ST + SP | -9.89 (7.24) | -311.31 (11.13) | -8.99 (3.84) | -312.25 (11.31) | 0.53 | 84.5 | 84 |
| c6.4 | SG + DPI + ASSAY + AGE + ST + SEX | -8.37 (6.83) | -309.8 (11.19) | -7.57 (3.62) | -310.83 (11.28) | 0.53 | 84.4 | 83.9 |
| c6.5 | SG + DPI + ASSAY + AGE + ST + TG | -8.33 (7.55) | -309.76 (11.33) | -7.31 (4.01) | -310.57 (11.48) | 0.54 | 84.5 | 84.1 |
| c7.1 | SG + DPI + ASSAY + AGE + ST + TG + CELL | -9.56 (7.57) | -310.99 (11.47) | -8.36 (4.02) | -311.62 (11.61) | 0.53 | 84.5 | 83.8 |
| c7.2 | SG + DPI + ASSAY + AGE + ST + TG + DOSE | -8.94 (7.49) | -310.37 (11.51) | -7.84 (3.95) | -311.1 (11.61) | 0.54 | 84.7 | 84.2 |
| c7.3 | SG + DPI + ASSAY + AGE + ST + TG + SP | -9.35 (7.08) | -310.77 (11.39) | -8.76 (3.78) | -312.02 (11.61) | 0.54 | 84.7 | 84.3 |
| c7.4 | SG + DPI + ASSAY + AGE + ST + TG + SEX | -7.14 (6.32) | -308.56 (11.46) | -6.8 (3.39) | -310.06 (11.63) | 0.54 | 84.7 | 84.3 |
| c8.1 | SG + DPI + ASSAY + AGE + ST + TG + SEX + CELL | -7.25 (5.87) | -308.67 (11.57) | -6.67 (3.13) | -309.93 (11.69) | 0.56 | 85 | 84.6 |
| c8.2 | SG + DPI + ASSAY + AGE + ST + TG + SEX + DOSE | -7.92 (6.52) | -309.35 (11.57) | -7.34 (3.45) | -310.6 (11.7) | 0.55 | 84.8 | 84.5 |
| c8.3 | SG + DPI + ASSAY + AGE + ST + TG + SEX + SP | -8.19 (5.88) | -309.62 (11.54) | -8.03 (3.19) | -311.29 (11.76) | 0.55 | 84.9 | 84.4 |
| c9.1 | SG + DPI + ASSAY + AGE + ST + TG + SEX + CELL + DOSE | -2.88 (4.95) | -304.3 (11.8) | -2.43 (2.57) | -305.69 (11.95) | 0.55 | 85.2 | 84.6 |
| c9.2 | SG + DPI + ASSAY + AGE + ST + TG + SEX + CELL + SP | -5.56 (3.71) | -306.99 (11.64) | -5.3 (2.01) | -308.56 (11.85) | 0.56 | 85.2 | 84.8 |
| **c10.1** | **SG + DPI + ASSAY + AGE + ST + TG + SEX + CELL + DOSE + SP** | **0 (0)** | **-301.42 (11.91)** | **0 (0)** | **-303.26 (12.15)** | **0.56** | **85.7** | **84.9** |
