# Supplementary material for: Predicting the presence of infectious virus from PCR data: A meta-analysis of SARS-CoV-2 in non-human primates
Source: PLoS Pathog. 2024 Apr 29;20(4):e1012171. doi: 10.1371/journal.ppat.1012171 (PMC11081500; doi:10.1371/journal.ppat.1012171)
Supplement: S6 Table — These intervals correspond with the predictions in Fig 3C and 3H. (DOCX) [file ppat.1012171.s026.docx]

| TotRNA | | | 0 | 1 | 2 | 3 | 4 | 5 | 6 | 7 | 8 | 9 | 10 |
| --- | --- | --- | --- | --- | --- | --- | --- | --- | --- | --- | --- | --- | --- |
| **sgRNA Logistic** | **Dose** | 4 | 0, 0 | 0, 1 | 1, 4 | 9, 19 | 43, 61 | 84, 92 | 97, 99 | 100, 100 | 100, 100 | 100, 100 | 100, 100 |
|  |  | 5.5 | 0, 0 | 0, 0 | 0, 1 | 2, 6 | 16, 27 | 6, 72 | 91, 95 | 98, 99 | 100, 100 | 100, 100 | 100, 100 |
|  |  | 7 | 0, 0 | 0, 0 | 0, 0 | 0, 2 | 4, 10 | 23, 43 | 69, 84 | 94, 98 | 99, 100 | 100, 100 | 100, 100 |
|  | **Species** | RM | 0, 0 | 0, 0 | 0, 1 | 2, 6 | 16, 27 | 6, 72 | 91, 95 | 98, 99 | 100, 100 | 100, 100 | 100, 100 |
|  |  | CM | 0, 0 | 0, 0 | 0, 1 | 2, 7 | 12, 33 | 5, 77 | 87, 96 | 98, 100 | 100, 100 | 100, 100 | 100, 100 |
|  |  | AGM | 0, 0 | 0, 0 | 0, 0 | 0, 2 | 3, 12 | 17, 49 | 59, 88 | 91, 98 | 98, 100 | 100, 100 | 100, 100 |
|  | **Target Gene** | T↑SG↑ | 0, 3 | 3, 17 | 18, 58 | 61, 91 | 91, 99 | 99, 100 | 100, 100 | 100, 100 | 100, 100 | 100, 100 | 100, 100 |
|  |  | T↓SG↑ | 0, 1 | 1, 8 | 6, 38 | 3, 81 | 75, 97 | 95, 100 | 99, 100 | 100, 100 | 100, 100 | 100, 100 | 100, 100 |
|  |  | T↑SG↓ | 0, 0 | 0, 0 | 0, 1 | 2, 6 | 16, 27 | 60, 72 | 91, 95 | 98, 99 | 100, 100 | 100, 100 | 100, 100 |
|  |  | T↓SG↓ | 0, 0 | 0, 0 | 0, 3 | 4, 15 | 24, 55 | 69, 90 | 94, 98 | 99, 100 | 100, 100 | 100, 100 | 100, 100 |
| **sgRNA Linear** | **Dose** | 4 | -1.16, -0.5 | -0.23, 0.39 | 0.7, 1.28 | 1.63, 2.17 | 2.55, 3.07 | 3.47, 3.97 | 4.38, 4.87 | 5.29, 5.79 | 6.2, 6.7 | 7.1, 7.62 | 8, 8.55 |
|  |  | 5.5 | -1.4, -0.88 | -0.46, 0 | 0.48, 0.89 | 1.41, 1.77 | 2.34, 2.66 | 3.27, 3.56 | 4.18, 4.46 | 5.09, 5.37 | 5.99, 6.29 | 6.88, 7.22 | 7.77, 8.15 |
|  |  | 7 | -1.76, -1.14 | -0.83, -0.25 | 0.1, 0.64 | 1.02, 1.53 | 1.94, 2.43 | 2.86, 3.33 | 3.77, 4.24 | 4.68, 5.15 | 5.58, 6.06 | 6.48, 6.98 | 7.38, 7.91 |
|  | **DPI** | I, 1 | -1.96, -1.39 | -1.02, -0.5 | -0.09, 0.38 | 0.84, 1.28 | 1.77, 2.17 | 2.69, 3.06 | 3.61, 3.97 | 4.52, 4.88 | 5.42, 5.79 | 6.32, 6.71 | 7.2, 7.64 |
|  |  | I, 2+ | -1.4, -0.88 | -0.46, 0 | 0.48, 0.89 | 1.41, 1.77 | 2.34, 2.66 | 3.27, 3.56 | 4.18, 4.46 | 5.09, 5.37 | 5.99, 6.29 | 6.88, 7.22 | 7.77, 8.15 |
|  |  | NI, 1+ | -1.01, -0.41 | -0.08, 0.47 | 0.86, 1.35 | 1.79, 2.24 | 2.72, 3.13 | 3.65, 4.03 | 4.57, 4.92 | 5.48, 5.83 | 6.38, 6.74 | 7.28, 7.66 | 8.18, 8.58 |
|  | **Target Gene** | T↓SG↑ | 0.01, 0.6 | 0.94, 1.49 | 1.87, 2.38 | 2.79, 3.28 | 3.71, 4.18 | 4.62, 5.09 | 5.53, 6 | 6.43, 6.91 | 7.33, 7.83 | 8.22, 8.76 | 9.11, 9.69 |
|  |  | T↑SG↓ | -1.4, -0.88 | -0.46, 0 | 0.48, 0.89 | 1.41, 1.77 | 2.34, 2.66 | 3.27, 3.56 | 4.18, 4.46 | 5.09, 5.37 | 5.99, 6.29 | 6.88, 7.22 | 7.77, 8.15 |
|  |  | T↓SG↓ | -1.05, -0.48 | -0.11, 0.4 | 0.81, 1.29 | 1.74, 2.19 | 2.66, 3.08 | 3.57, 3.99 | 4.48, 4.9 | 5.39, 5.82 | 6.28, 6.73 | 7.18, 7.66 | 8.07, 8.59 |
|  | **Species** | RM | -1.4, -0.88 | -0.46, 0 | 0.48, 0.89 | 1.41, 1.77 | 2.34, 2.66 | 3.27, 3.56 | 4.18, 4.46 | 5.09, 5.37 | 5.99, 6.29 | 6.88, 7.22 | 7.77, 8.15 |
|  |  | CM | -2.42, -1.74 | -1.49, -0.86 | -0.55, 0.03 | 0.38, 0.92 | 1.3, 1.81 | 2.23, 2.71 | 3.14, 3.61 | 4.05, 4.52 | 4.96, 5.43 | 5.86, 6.34 | 6.76, 7.26 |
|  |  | AGM | -1.36, -0.65 | -0.43, 0.23 | 0.5, 1.12 | 1.43, 2.01 | 2.35, 2.9 | 3.28, 3.8 | 4.2, 4.7 | 5.11, 5.61 | 6.02, 6.51 | 6.92, 7.43 | 7.82, 8.35 |
