# Supplementary material for: Predicting the presence of infectious virus from PCR data: A meta-analysis of SARS-CoV-2 in non-human primates
Source: PLoS Pathog. 2024 Apr 29;20(4):e1012171. doi: 10.1371/journal.ppat.1012171 (PMC11081500; doi:10.1371/journal.ppat.1012171)
Supplement: S7 Table — These were generated for the models without a lab effect. (DOCX) [file ppat.1012171.s027.docx]

| Model | Parameter | 5% CI | Median | 95% CI | Standard Dev. |
| --- | --- | --- | --- | --- | --- |
| **PCR Logistic** | intercept | -3.8 | -2.15 | -0.49 | 1 |
|  | T | 1.75 | 1.96 | 2.19 | 0.13 |
|  | DOSE | -1.16 | -0.93 | -0.71 | 0.14 |
|  | SP [RM] | -0.59 | 0.4 | 1.41 | 0.61 |
|  | SP [CM] | -0.65 | 0.36 | 1.36 | 0.61 |
|  | SP [AGM] | -2.14 | -1.06 | -0.04 | 0.64 |
|  | TG [T↑ SG↑] | 1.23 | 2.32 | 3.41 | 0.66 |
|  | TG [T↓ SG↑] | 0.13 | 1.24 | 2.35 | 0.68 |
|  | TG [T↑ SG↓] | -3.29 | -2.32 | -1.38 | 0.58 |
|  | TG [T↓ SG↓] | -2.46 | -1.51 | -0.57 | 0.58 |
| **PCR Linear** | intercept | -2.48 | -1.25 | -0.03 | 0.75 |
|  | T | 0.88 | 0.91 | 0.94 | 0.02 |
|  | DOSE | -0.34 | -0.21 | -0.08 | 0.08 |
|  | DPI [I, 1] | -0.81 | 0.08 | 0.95 | 0.54 |
|  | DPI [I, 2+] | -0.26 | 0.61 | 1.5 | 0.54 |
|  | DPI [NI, 1+] | 0.14 | 1.03 | 1.93 | 0.54 |
|  | SP [RM] | -0.05 | 0.85 | 1.75 | 0.55 |
|  | SP [CM] | -0.99 | -0.09 | 0.8 | 0.54 |
|  | SP [AGM] | 0.07 | 0.98 | 1.88 | 0.55 |
|  | TG [T↓ SG↑] | 0.36 | 1.25 | 2.14 | 0.53 |
|  | TG [T↑ SG↓] | -1.09 | -0.19 | 0.71 | 0.54 |
|  | TG [T↓ SG↓] | -0.71 | 0.18 | 1.06 | 0.54 |
| **Culture** | intercept | -1.56 | -0.04 | 1.47 | 0.92 |
|  | T | 0.69 | 0.8 | 0.91 | 0.07 |
|  | DOSE | -1.19 | -0.85 | -0.52 | 0.2 |
|  | DPI [I, 1] | 0.41 | 1.39 | 2.4 | 0.61 |
|  | DPI [I, 2+] | -1.09 | -0.14 | 0.83 | 0.58 |
|  | DPI [NI, 1+] | -1.26 | -0.28 | 0.69 | 0.59 |
|  | SP [RM] | -0.18 | 0.79 | 1.77 | 0.59 |
|  | SP [CM] | -1.58 | -0.55 | 0.46 | 0.62 |
|  | SP [AGM] | -0.28 | 0.72 | 1.74 | 0.61 |
|  | AGE [Juvenile] | -1.53 | -0.48 | 0.57 | 0.64 |
|  | AGE [Adult] | -1.93 | -0.94 | 0.07 | 0.61 |
|  | AGE [Geriatric] | 1.2 | 2.39 | 3.57 | 0.72 |
|  | TG [N] | -0.56 | 0.51 | 1.55 | 0.65 |
|  | TG [E] | -1.73 | -0.69 | 0.31 | 0.62 |
|  | TG [S] | 0.06 | 1.13 | 2.23 | 0.66 |
|  | ASSAY | -2.56 | -1.75 | -0.96 | 0.49 |
|  | CELL [76] | -0.48 | 0.54 | 1.56 | 0.61 |
|  | CELL [E6] | -1.81 | -0.78 | 0.27 | 0.63 |
|  | CELL [E6-SS2] | 0.49 | 1.71 | 2.92 | 0.73 |
