# Supplementary material for: Predicting the presence of infectious virus from PCR data: A meta-analysis of SARS-CoV-2 in non-human primates
Source: PLoS Pathog. 2024 Apr 29;20(4):e1012171. doi: 10.1371/journal.ppat.1012171 (PMC11081500; doi:10.1371/journal.ppat.1012171)
Supplement: S8 Table — The group number used to display lab effects in S12A and S12E, and S16A Figs are provided in the first column. The number for culture analyses (C) precedes the one for the sgRNA analyses (SG). (DOCX) [file ppat.1012171.s028.docx]

| Lab number  (C / SG) | Articles (ref.) | Location of primate studies  (if multiple articles) |
| --- | --- | --- |
| 1 / -- | Deng et al. 2020 (1) |  |
| 2 / -- | Ishigaki et al. 2021 (2) |  |
| 3 / -- | Johnston et al. 2020 (3) |  |
| 4 / -- | Kobiyama et al. 2021 (4) |  |
| 5 / 3 | Jiao et al. 2021 (5);  Nagata et al. 2021 (6) | Kunming Primate Center |
| 6 / 5 | Salguero et al. 2021 (7) |  |
| 7 / 6 | Munster et al. 2020 (8);  Speranza et al. 2020 (9);  van Doremalen et al. 2020 (10);  Williamson et al. 2020 (11) | Rocky Mountain Laboratories |
| 8 / -- | Shan et al. 2020 (12) |  |
| 9 / 7 | Singh et al. 2020 (13) |  |
| 10 / -- | Cross et al. 2020 (14);  Woolsey et al. 2020 (15) | University of Texas Medical Branch, Galveston |
| -- / 1 | Gabitzsch et al. 2021 (16) |  |
| -- / 2 | Baum et al. 2020 (17);  Chandrashekar et al. 2020 (18);  Corbett et al. 2020 (19);  Dagotto et al. 2020 (20);  Jones et al. 2021 (21);  Patel et al. 2021 (22) | Bioqual |
| -- / 4 | Li et al. 2021 (23) |  |
| -- / 8 | Yu et al. 2020 (24) |  |

**References**

1. Deng W, Bao L, Gao H, Xiang Z, Qu Y, Song Z, et al. Ocular conjunctival inoculation of SARS-CoV-2 can cause mild COVID-19 in rhesus macaques. Nat Commun. 2020 Dec;11(1):4400–4400.
2. Ishigaki H, Nakayama M, Kitagawa Y, Nguyen CT, Hayashi K, Shiohara M, et al. Neutralizing antibody-dependent and -independent immune responses against SARS-CoV-2 in cynomolgus macaques. Virology. 2021 Feb 1;554:97–105.
3. Johnston SC, Ricks KM, Jay A, Raymond JL, Rossi F, Zeng X, et al. Development of a coronavirus disease 2019 nonhuman primate model using airborne exposure. PLOS ONE. 2021 Feb 2;16(2):e0246366.
4. Kobiyama K, Imai M, Jounai N, Nakayama M, Hioki K, Iwatsuki-Horimoto K, et al. Optimization of an LNP-mRNA vaccine candidate targeting SARS-CoV-2 receptor-binding domain. bioRxiv. 2021. Available from: <https://www.biorxiv.org/content/10.1101/2021.03.04.433852v1>
5. Jiao L, Li H, Xu J, Yang M, Ma C, Li J, et al. The Gastrointestinal Tract Is an Alternative Route for SARS-CoV-2 Infection in a Nonhuman Primate Model. Gastroenterology. 2021 Apr 1;160(5):1647–61.
6. Nagata N, Iwata-Yoshikawa N, Sano K, Ainai A, Shiwa N, Shirakura M, et al. The peripheral T cell population is associated with pneumonia severity in cynomolgus monkeys experimentally infected with severe acute respiratory syndrome coronavirus 2. bioRxiv. 2021. Available from: <https://www.biorxiv.org/content/10.1101/2021.01.07.425698v1>
7. Salguero FJ, White AD, Slack GS, Fotheringham SA, Bewley KR, Gooch KE, et al. Comparison of rhesus and cynomolgus macaques as an infection model for COVID-19. Nat Commun. 2021 Feb 24;12(1):1260.
8. Munster VJ, Feldmann F, Williamson BN, van Doremalen N, Pérez-Pérez L, Schulz J, et al. Respiratory disease in rhesus macaques inoculated with SARS-CoV-2. Nature. 2020 Sep;585(7824):268–72.
9. Speranza E, Williamson BN, Feldmann F, Sturdevant GL, Pérez LP, Meade-White K, et al. Single-cell RNA sequencing reveals SARS-CoV-2 infection dynamics in lungs of African green monkeys. Sci Transl Med. 2021 Jan 27;13(578).
10. van Doremalen N, Lambe T, Spencer A, Belij-Rammerstorfer S, Purushotham JN, Port JR, et al. ChAdOx1 nCoV-19 vaccine prevents SARS-CoV-2 pneumonia in rhesus macaques. Nature. 2020 Jul;1–8.
11. Williamson BN, Feldmann F, Schwarz B, Meade-White K, Porter DP, Schulz J, et al. Clinical benefit of remdesivir in rhesus macaques infected with SARS-CoV-2. Nature. 2020 Jun;1–7.
12. Shan C, Yao YF, Yang XL, Zhou YW, Gao G, Peng Y, et al. Infection with novel coronavirus (SARS-CoV-2) causes pneumonia in Rhesus macaques. Cell Res. 2020 Jul;1–8.
13. Singh DK, Singh B, Ganatra SR, Gazi M, Cole J, Thippeshappa R, et al. Responses to acute infection with SARS-CoV-2 in the lungs of rhesus macaques, baboons and marmosets. Nat Microbiol. 2021 Jan;6(1):73–86.
14. Cross RW, Agans KN, Prasad AN, Borisevich V, Woolsey C, Deer DJ, et al. Intranasal exposure of African green monkeys to SARS-CoV-2 results in acute phase pneumonia with shedding and lung injury still present in the early convalescence phase. Virol J. 2020 Dec;17(1):125–125.
15. Woolsey C, Borisevich V, Prasad AN, Agans KN, Deer DJ, Dobias NS, et al. Establishment of an African green monkey model for COVID-19 and protection against re-infection. Nat Immunol. 2021 Jan;22(1):86–98.
16. Gabitzsch E, Safrit JT, Verma M, Rice A, Sieling P, Zakin L, et al. Dual-Antigen COVID-19 Vaccine Subcutaneous Prime Delivery With Oral Boosts Protects NHP Against SARS-CoV-2 Challenge. Front Immunol. 2021;12.
17. Baum A, Ajithdoss D, Copin R, Zhou A, Lanza K, Negron N, et al. REGN-COV2 antibodies prevent and treat SARS-CoV-2 infection in rhesus macaques and hamsters. Science. 2020 Nov 27;370(6520):1110–5.
18. Chandrashekar A, Liu J, Martinot AJ, McMahan K, Mercado NB, Peter L, et al. SARS-CoV-2 infection protects against rechallenge in rhesus macaques. Science. 2020 May;eabc4776–eabc4776.
19. Corbett KS, Flynn B, Foulds KE, Francica JR, Boyoglu-Barnum S, Werner AP, et al. Evaluation of the mRNA-1273 Vaccine against SARS-CoV-2 in Nonhuman Primates. N Engl J Med. 2020 Jul;NEJMoa2024671–NEJMoa2024671.
20. Dagotto G, Mercado NB, Martinez DR, Hou YJ, Nkolola JP, Carnahan RH, et al. Comparison of Subgenomic and Total RNA in SARS-CoV-2-Challenged Rhesus Macaques. J Virol. 2021 Mar 25;95(8).
21. Jones BE, Brown-Augsburger PL, Corbett KS, Westendorf K, Davies J, Cujec TP, et al. The neutralizing antibody, LY-CoV555, protects against SARS-CoV-2 infection in nonhuman primates. Sci Transl Med. 2021 May 12;13(593):eabf1906.
22. Patel A, Walters JN, Reuschel EL, Schultheis K, Parzych E, Gary EN, et al. Intradermal-delivered DNA vaccine induces durable immunity mediating a reduction in viral load in a rhesus macaque SARS-CoV-2 challenge model. Cell Rep Med. 2021 Oct 19;2(10):100420.
23. Li D, Edwards RJ, Manne K, Martinez DR, Schäfer A, Alam SM, et al. In vitro and in vivo functions of SARS-CoV-2 infection-enhancing and neutralizing antibodies. Cell. 2021 Aug 5;184(16):4203-4219.e32.
24. Yu J, Tostanoski LH, Peter L, Mercado NB, McMahan K, Mahrokhian SH, et al. DNA vaccine protection against SARS-CoV-2 in rhesus macaques. Science. 2020 May;eabc6284–eabc6284.
