# Supplementary material for: Predicting the presence of infectious virus from PCR data: A meta-analysis of SARS-CoV-2 in non-human primates
Source: PLoS Pathog. 2024 Apr 29;20(4):e1012171. doi: 10.1371/journal.ppat.1012171 (PMC11081500; doi:10.1371/journal.ppat.1012171)
Supplement: S9 Table — Statistics are stratified by predictor(s) and the dataset used for fitting, including the full dataset (based on sgRNA predictions; ‘all data’) and the subset containing only samples with known sgRNA and totRNA results (‘data subset’). Prediction accuracy reflects aggregate performance on test data across the full 10 train-test folds, stratified by all available samples (Overall), only known positive samples, and only known negative samples. MCC corresponds to the Matthews correlation coefficient. Note that we do not report ELPD because these models were fit with different quantities of data and so ELPD is not comparable. * includes imputed data. † includes data with observed sgRNA outcomes but no observed totRNA outcomes. (DOCX) [file ppat.1012171.s029.docx]

|  | Predictors | Prediction Accuracy (%) | | | MCC |
| --- | --- | --- | --- | --- | --- |
|  |  | **Overall** | **Positive** | **Negative** |  |
| **Data subset** | T | 90.4 | 72.2 | 94.3 | 0.67 |
|  | SG | 90.0 | 70.4 | 94.3 | 0.66 |
|  | T + SG | 90.0 | 68.5 | 94.7 | 0.65 |
| **All**  **data** | T | 81.9 | 53.7 | 91.4 | 0.49 |
|  | SG*^†^ | 80.8 | 46.7 | 91.7 | 0.43 |
|  | T + SG* | 81.7 | 51.8 | 91.7 | 0.48 |
|  | Best T | 84.7 | 60.7 | 92.8 | 0.57 |
|  | Best SG*^†^ | 84.9 | 57.7 | 93.6 | 0.56 |
