# Supplementary material for: Predicting the presence of infectious virus from PCR data: A meta-analysis of SARS-CoV-2 in non-human primates
Source: PLoS Pathog. 2024 Apr 29;20(4):e1012171. doi: 10.1371/journal.ppat.1012171 (PMC11081500; doi:10.1371/journal.ppat.1012171)
Supplement: S10 Table — These intervals correspond with the predictions in Fig 5C. (DOCX) [file ppat.1012171.s030.docx]

| TotRNA | | 0 | 1 | 2 | 3 | 4 | 5 | 6 | 7 | 8 | 9 | 10 | 11 | 12 |
| --- | --- | --- | --- | --- | --- | --- | --- | --- | --- | --- | --- | --- | --- | --- |
| **Dose** | 4 | 0, 4 | 1, 7 | 2, 14 | 4, 26 | 10, 44 | 19, 63 | 35, 79 | 55, 90 | 72, 95 | 85, 98 | 92, 99 | 96, 100 | 98, 100 |
|  | 5.5 | 0, 1 | 0, 2 | 1, 4 | 2, 7 | 4, 15 | 8, 27 | 17, 44 | 31, 64 | 50, 80 | 68, 90 | 82, 96 | 90, 98 | 95, 99 |
|  | 7 | 0, 0 | 0, 1 | 0, 1 | 0, 2 | 1, 5 | 2, 10 | 5, 19 | 11, 33 | 21, 52 | 37, 71 | 56, 85 | 73, 93 | 85, 97 |
| **DPI** | I, 1 | 0, 5 | 1, 9 | 3, 18 | 6, 31 | 13, 49 | 25, 67 | 43, 82 | 63, 91 | 79, 96 | 89, 98 | 95, 99 | 97, 100 | 99, 100 |
|  | I, 2+ | 0, 1 | 0, 2 | 1, 4 | 2, 7 | 4, 15 | 8, 27 | 17, 44 | 31, 64 | 50, 80 | 68, 90 | 82, 96 | 90, 98 | 95, 99 |
|  | NI, 1+ | 0, 1 | 0, 2 | 1, 3 | 1, 7 | 3, 13 | 7, 24 | 15, 41 | 28, 61 | 46, 78 | 65, 89 | 80, 95 | 89, 98 | 95, 99 |
| **Species** | RM | 0, 1 | 0, 2 | 1, 4 | 2, 7 | 4, 15 | 8, 27 | 17, 44 | 31, 64 | 50, 80 | 68, 90 | 82, 96 | 90, 98 | 95, 99 |
|  | CM | 0, 0 | 0, 1 | 0, 1 | 0, 2 | 1, 5 | 2, 9 | 4, 19 | 9, 33 | 19, 53 | 34, 72 | 52, 85 | 70, 93 | 83, 97 |
|  | AGM | 0, 1 | 0, 2 | 1, 3 | 2, 6 | 4, 12 | 9, 21 | 20, 36 | 36, 55 | 55, 73 | 73, 87 | 85, 94 | 92, 97 | 96, 99 |
| **Age**  **Class** | Juvenile | 0, 1 | 1, 3 | 1, 5 | 3, 10 | 6, 19 | 14, 33 | 27, 52 | 45, 71 | 64, 85 | 79, 93 | 89, 97 | 94, 99 | 97, 99 |
|  | Adult | 0, 1 | 0, 2 | 1, 4 | 2, 7 | 4, 15 | 8, 27 | 17, 44 | 31, 64 | 50, 80 | 68, 90 | 82, 96 | 90, 98 | 95, 99 |
|  | Geriatric | 3, 24 | 6, 40 | 13, 59 | 25, 76 | 42, 87 | 62, 94 | 78, 97 | 89, 99 | 94, 99 | 97, 100 | 99, 100 | 99, 100 | 100, 100 |
| **Cell Line** | 76 | 0, 1 | 0, 2 | 1, 4 | 2, 7 | 4, 15 | 8, 27 | 17, 44 | 31, 64 | 50, 80 | 68, 90 | 82, 96 | 90, 98 | 95, 99 |
|  | E6 | 0, 0 | 0, 1 | 0, 1 | 0, 2 | 1, 4 | 2, 9 | 5, 17 | 11, 31 | 23, 49 | 39, 68 | 59, 83 | 75, 92 | 87, 96 |
|  | E6-SS2 | 0, 4 | 1, 7 | 2, 14 | 4, 26 | 8, 43 | 16, 62 | 31, 78 | 49, 89 | 68, 95 | 82, 98 | 91, 99 | 95, 100 | 98, 100 |
| **Assay** | TCID50 | 1, 5 | 2, 10 | 4, 18 | 8, 33 | 17, 51 | 32, 70 | 51, 84 | 69, 92 | 83, 96 | 91, 98 | 96, 99 | 98, 100 | 99, 100 |
|  | Plaque | 0, 1 | 0, 2 | 1, 4 | 2, 7 | 4, 15 | 8, 27 | 17, 44 | 31, 64 | 50, 80 | 68, 90 | 82, 96 | 90, 98 | 95, 99 |
| **Target**  **Gene** | N | 0, 1 | 0, 2 | 1, 4 | 2, 7 | 4, 15 | 8, 27 | 17, 44 | 31, 64 | 50, 80 | 68, 90 | 82, 96 | 90, 98 | 95, 99 |
|  | E | 0, 0 | 0, 1 | 0, 2 | 0, 4 | 1, 7 | 2, 15 | 3, 28 | 8, 46 | 15, 65 | 28, 81 | 47, 91 | 65, 96 | 80, 98 |
|  | S | 0, 2 | 0, 4 | 1, 9 | 2, 17 | 5, 31 | 10, 49 | 20, 68 | 36, 83 | 55, 92 | 72, 96 | 85, 98 | 92, 99 | 96, 100 |
