# Supplementary material for: Predicting the presence of infectious virus from PCR data: A meta-analysis of SARS-CoV-2 in non-human primates
Source: PLoS Pathog. 2024 Apr 29;20(4):e1012171. doi: 10.1371/journal.ppat.1012171 (PMC11081500; doi:10.1371/journal.ppat.1012171)
Supplement: S11 Table — For every inoculation route, only the tissues with data available for that route are displayed. Because fluid is administered in the trachea for intratracheal (IT) inoculations, which is connected directly to the bronchioles, we include bronchus as an exposure tissue for IT inoculations. We also consider BAL an inoculated tissue for IT exposures since this procedure collects fluid from similar areas where the inoculum is administered. Exposure route abbreviations are: AE, aerosol; IT, intratracheal; IN, intranasal; IG, intragastric; OC, ocular; OR, oral. (DOCX) [file ppat.1012171.s031.docx]

| Inoculation Route(s) | Inoculated Locations | Non-inoculated Locations |
| --- | --- | --- |
| AE | Nose/Nasopharynx, Oropharynx | Anus/Rectum |
| IT |  | Anus/Rectum, Lung, Nose/Nasopharynx, Oropharynx |
| IN | Nose/Nasopharynx | Anus/Rectum, BAL, Colon, Mouth, Small intestine, Stomach |
| IG | Stomach | Anus/Rectum, Colon, Small intestine |
| OC |  | Lung |
| IT, IN | BAL, Nose/Nasopharynx, Oropharynx, Trachea | Anus/Rectum, Lung, Mouth, Tonsil |
| IT, IN, OC | Eye, Nose/Nasopharynx, Oropharynx, Trachea | Anus/Rectum, Brain, Cervical LN, Colon, Kidney, Liver, Lung, Mesenteric LN, Salivary gland, Small intestine, Spleen, Tonsil |
| IT, IN, OR, OC | BAL, Bronchus, Eye, Mouth, Nose/Nasopharynx, Oropharynx, Tonsil, Trachea | Anus/Rectum, Cervical LN, Colon, Heart, Lung, Mediastinal LN, Small intestine, Stomach |
