# Supplementary material for: Predicting the presence of infectious virus from PCR data: A meta-analysis of SARS-CoV-2 in non-human primates
Source: PLoS Pathog. 2024 Apr 29;20(4):e1012171. doi: 10.1371/journal.ppat.1012171 (PMC11081500; doi:10.1371/journal.ppat.1012171)
Supplement: Attachment — Submitted filename: reviewer-responses.pdf [file ppat.1012171.s032.pdf]

We thank the reviewers and the editor for their comments, and we are very grateful for the opportunity to resubmit our revised manuscript. In the blue text below, please find a general description of our changes followed by our full point-by-point response to the reviewers' comments.

We have added one new figure to the main text (Figure 7), which displays the results from our analyses on more clinically relevant metrics, as requested by the reviewers and editor. We have also added one supplementary figure to support these clinical analyses (Figure S18), in addition to another two supplementary figures that support our analysis of lab effects (Figures S12, S16) and one that further compares the performance of the simple and best culture models (Figure S15). We have also added two new supplementary tables. One shows how we grouped articles into labs for our lab effect analyses (Table S8), while the other provides the median and credible intervals for all parameters estimated by our best models (Table S7). All of our text changes to accommodate new analyses and to address the reviewers' comments are noted with red text in the new manuscript file.

Although the authors perform a detailed meta-analysis to predict infectious virus from PCR data, the reviewers (nr1 and nr 2) and the editor found the focus on non human primate samples and lack of a clinical application a major weakness of the study. I am afraid that in the absence of evidence that (as stated by the authors in the discussion, line 600) "The framework also shows particular potential for clinical diagnostics... ", I have to reject this version of the manuscript for publication in PLOS Pathogens.

We thank the editors and the reviewers for this feedback, which has made us realize we need to communicate our study's goals and contributions more clearly. We did not intend to suggest that our quantitative model results can or should be used directly for generating predictions on human data, or that our model trained on NHP data is immediately applicable to humans. We have focused our study on non-human primates because they are the gold standard animal model for human infections, and NHP studies play an irreplaceable role in studies from basic pathology to preclinical drug and vaccine trials. Thus, our focus on NHP studies brings the advantages of animal challenge experiments (controlled conditions; known dose, route, and timing of inoculation; and opportunities for repeated, invasive, or lethal sampling) with highest relevance to human health (both as a model for humans, as NHP studies are interpreted widely in the literature, and by directly supporting trials of drugs and vaccines). Our primary goals are:

(i) to probe the fundamental relationship between PCR assays and presence of infectious virus *in vivo*, in the most relevant experimental model, and in particular to address the debate about the relative value of sgRNA data.
(ii) to provide a quantitative tool to support the analysis and interpretation of SARS-CoV-2 NHP challenge studies, strengthening the inferences that can be drawn from PCR data in such studies, and addressing outstanding concerns about the effects of inoculation dose and route in these studies.
(iii) to offer our approach as a standardized framework for developing models to analyze similar relationships in other pathogen-host systems, which could be modified to suit their particular characteristics, and which (after being trained on the relevant data) could possibly achieve similar aims to (i) and (ii) in those other systems.
(iv) to provide a case study showing the power (and feasibility) of meta-analyses of virological data, which will become essential techniques as data-sharing becomes the norm in virology, and which help to surmount challenges arising from necessarily small sample sizes in NHP studies.

Regarding clinical applications to humans, we believe our analysis contributes to the literature on some salient questions (such as the interpretation of sgRNA vs. total RNA levels, and the potential for accurate prediction of culture results from PCR data) and shows the potential for real-world applications such as determining when to end case isolation. But quantitative application of our model framework to human data was intended as a future direction, which could be built on the foundation of the current work. We apologize that we did not make that sufficiently clear.

In sum, our primary goal with this work is to advance fundamental understanding of the biology of SARS-CoV-2 in primates and to provide methods to achieve similar understanding in other systems. We believe that the clinical applications will follow in future studies. Indeed, we submitted the work to *PLOS Pathogens*, rather than a more clinically oriented journal, because we believed the work aligned well with the stated aim of the journal to publish "work with a strong emphasis on discovery that informs our understanding of pathogenesis or pathogen biology." We have taken two broad approaches to revising the manuscript in response to the concerns expressed about clinical applicability, namely by clarifying our study's intent and contributions, and by adding new analyses that illustrate the potential for our methods to lead to clinically meaningful tools in future studies.

1. We have made revisions throughout our manuscript to clarify the study's goals and contributions, and to clarify that our NHP model results should not be used directly for generating predictions on human data. Instead, the model should be adapted and re-trained on human data (which will differ in important ways from NHP challenge data), where its performance will guide the potential application of our framework in clinical settings. We offer thoughts about these future directions in the discussion (lines 820-853). Notably, this will require the compilation and in-depth analysis of an analogous database from humans, which constitutes an entirely new study of comparable scale to the current manuscript. We have been careful in our discussion to present this clearly as a future direction.

2. To provide an explicit example of the potential for clinical application, we have conducted new analyses of our model predictions using clinically relevant metrics, which are presented in a new section in the results (*The best culture model shows potential for accurate, individualized isolation practices;* lines 584-642). We focus on our model's ability to predict when an individual is no longer infectious (i.e., no longer tests culture positive). Relative to standard clinical protocols of isolating for ten or five days after the first positive test for SARS-CoV-2, we show that our model can reduce the number of days spent isolating, thus limiting personal and economic burdens imposed by prolonged isolation. Our approach can also reduce the number of days that individuals may still be infectious after release from isolation, compared to the five-day protocol. Given our model's strong performance on non-human primates, which are the gold standard animal model for human disease, we believe our model framework shows promise for clinical settings, though we emphasize that this is an illustrative analysis and requires confirmation from a comparable study run on human data.

We hope that this combination of greater clarity on our goals and contributions, and a more explicit example of the potential for future clinical relevance, will address the concerns about clinical applicability raised by the reviewers and editor. We think that this contribution is well aligned with the stated mission of *PLOS Pathogens*, which we also believe is the ideal journal for our work to reach the scientists that would be most interested in our findings, namely those studying experimental virology, pathogenesis, and infectious disease dynamics.

Part I - Summary
Please use this section to discuss strengths/weaknesses of study, novelty/significance, general execution, and scholarship.

Reviewer #1:

The study used various experimental data in NHP models to predict infectious viruses.

Reviewer #2:

In this manuscript, Snedden and Lloyd-Smith perform a meta-analysis of 24 studies and develop Bayesian statistical models to evaluate the utility of molecular markers as predictors of cell culture infectivity of SARS-CoV-2 from 3 species of experimentally infected non-human primates. The gold standard for infectivity of SARS-CoV-2 is demonstration of viral viability in cell culture. Molecular assays to detect infectivity would obviate the need for time consuming and expensive cell culture techniques that are not practical in a clinical setting. The authors are specifically interested in the utility of RT-qPCR assays of total SARS-CoV-2 RNA which represent the vast majority of RT-qPCRs tests used in a clinical setting. The authors also evaluate the utility of RT-qPCR assays of subgenomic RNA because early studies suggested that it may more closely reflect duration of infectivity. Subgenomic RNA assays, have not been used in clinical diagnostics despite multiple papers on the subject. The authors indicate their model can be adapted to support medical decisions and public health guidelines and generate predictions on infectiousness.

The authors results show that their models can predict subgenomic RNA results from total RNA and predict culture positivity from these two RT-qPCR assays with a high degree of accuracy. They explore multiple cofactors including host characteristics, and assays in their model and highlight the importance of these cofactors in culture prediction.

The strength of this study that the authors use a large number of data points under controlled conditions. The study is carefully conducted, and the data is fully explored, with extensive analysis, including in-depth exploration of reasons for unexpected patterns seen in the data. It does seem that this type of analysis would be a powerful tool especially if applied to the accumulating data from clinical samples and especially so in populations where duration of infectivity is less clear such as in immunosuppressed hosts or during infection with different viral variants. The manuscript is well written.

The study has some limitations which are mostly well outlined in the discussion. Because the authors indicate that their model can be used to support medical decisions and public health guidelines, they should address the difficulty in using rt-qPCR assays in clinical decision making. There is likely a copy number of sgRNA and TotRNA where patients are no longer infectious. However, in clinical practice RT-qPCR assays have not generally been used to determine infectivity for many reasons. One reason is that it is very difficult to compare rt-qPCR results (either copy number or CT value) across labs without a universal calibrator for viral load. It also remains to be seen whether predictions models using Rt-qPCR data can improve the ability to predict infectivity allowing patients to be removed from in-hospital isolation in less than the current 10 days recommended for immunocompetent hosts. If possible, it may strengthen the manuscript to specifically indicate how the best model improves predictions on duration of infectivity when compared to the simple model.

Thank you for this succinct and perceptive summary of our work, and its strengths and limitations. We hope our response above, and the more detailed responses and revisions below, help to clarify how we are thinking about applications to clinical data. We have added a new analysis specifically targeted to comparing decision rules for the end of isolation, including the 'simple' and 'best' models based on RT-qPCR data, and both 5- and 10-day rules that have been in effect for SARS-CoV-2 isolation. We believe this analysis (shown in Figure 7) is a valuable addition to the study, and we're grateful for the suggestion.

Reviewer #3:

Snedden and Lloyd-Smith investigated the relationship between total RNA, sub-genomic RNA and infectious culture positivity by compiling experimental data from 24 studies of SARS-CoV-2 infection in non-human primates. They developed Bayesian statistical models to quantify their relationships and evaluated how co-factors, such as exposure conditions, host traits, and assay protocols, influence model predictions. The main conclusion of the work is that total RNA as measured by PCR can predict culture positivity with a high accuracy and they did not find evidence that the sgRNA level is a superior predictor of culture positivity.

Overall, the work addresses an important question during the COVID-19 pandemic, i.e. how various measures of SARS-CoV-2 load could be used to estimate infectiousness of individuals. The model and results here will be very useful to understand the various assays and how their results could be used to make clinical or public health decisions in the pandemic. The work is well conducted, and well-written.

Thank you for this positive assessment of our work.

Part II – Major Issues: Key Experiments Required for Acceptance
Please use this section to detail the key new experiments or modifications of existing experiments that should be absolutely required to validate study conclusions.

Reviewer #1:

The reviewer has the concerning concerns:

1. How does it relevant to human infection?

Thank you for this suggestion. In response to this critique as well as concerns posed by the editor and other reviewers, we have made substantial changes in our manuscript to clarify that our NHP results were not intended to be used for generating predictions in humans. However, we did include a new paragraph in our discussion that draws parallels between our results and patterns that have been previously noted in human studies. For example, our simple model predicts that samples with around 7 log10 totRNA copies have approximately 50% chance of being culture positive, which is very similar to quantities noted in human data (5-9 log10 RNA copies). Please find the full details on lines 755-770.

To further probe the potential relevance of our modeling approach for human infection, we also conducted additional analyses that characterized our model's performance on more clinically relevant metrics – namely, its ability to detect when an individual is no longer infectious. Our best culture model performed remarkably well, showing clear improvements over standard guidelines of isolating for five or ten days after the first positive test. While this suggests our framework could possibly be useful in clinical settings, it must first be modified to handle the unique complexities of human systems, after which it must then be rigorously tested on human data. Please find the details of these analyses and our suggestions for future work to test our approach for clinical settings in lines 584-642 and 820-853, respectively.

Finally, we note (and have noted in lines 858-862 in the manuscript) that NHPs are the gold standard experimental model for preclinical trials of vaccines and therapeutics, and, as a consequence, any improvements to the scientific foundation of NHP challenge studies will have direct bearing on human health. This was indisputably the case for SARS-CoV-2, and indeed much of our data was derived from the control arms of NHP preclinical studies.

2. Considering most of the human individuals have prior immune responses, either by infection or vaccination, what is the potential use of their findings in human infections?

If and when a future study applies our approach to human data, our model framework can definitely include prior infection and vaccination history as candidate cofactors to determine whether / how they impact the relationship between RNA quantities and culture outcomes. The fundamental question could also be investigated using non-human primate data, given that primate challenge studies are widely used in preclinical trials for candidate vaccines. Because the data used in the current study only includes unvaccinated and naïve individuals, it is difficult to speculate on the possible effects of immunity. However, we have mentioned them in our discussion as key cofactors to include if the model is extended to humans. This reads (lines 839-842):

"To realize the clinical potential of this approach, however, the model framework must be trained on human data. This would involve some model modifications, including the consideration of other cofactors such as viral variant, prior infection, vaccination history, disease severity, and co-morbidities."

3. The authors used a vast number of NHP studies in the analyses. But for major outbreaks (or pandemics), it would be much easier to have clinical samples from infected cased to demonstrate "infectivity". How can their findings help to develop a better strategy to control/contain a disease in future?

As clarified above, the current study is focused on the fundamental question and establishing this quantitative modeling framework. For these purposes, the controlled conditions of NHP studies are ideal. We agree that our framework could be adapted for use in an outbreak scenario to design evidence-based guidelines for isolation. You are quite correct that the model could be immediately tested in such a scenario on clinical data, instead of waiting for NHP studies. We have outlined this approach in our revised discussion on lines (lines 849-853):

"Once the modeling pipeline is established, it could be readily tailored to any other pathogen with sufficient clinical data, either to improve management strategies of existing

viruses or even to help characterize and contain an emerging one. With these tools, public health officials and clinicians would be better-equipped to weigh transmission risk with medical resource availability and economic burden to designate evidence-based (and pathogen-specific) hospital discharge criteria and public health guidelines."

4. Consider different respiratory virus infections are different clinical profile, the reviewer is not sure whether the authors intend to restrict the interpretations to COVID-19 or beyond. Please clarify.

Thank you for sharing this concern, as it helped us realized that we had not sufficiently explained our aims or the scope of our quantitative results. We did not intend to extend the specific interpretations of our model outputs to any pathogen-host systems outside of SARS-CoV-2 in non-human primates. We have made many small text edits in our discussion to clarify that the particular results apply to SARS-CoV-2 and non-human primates. However, we do believe our framework can be used with analogous data in other systems to determine which biological and methodological factors drive the relationships between key assays, and potentially to develop quantitative tools to make clinically-relevant decisions. We have added a sentence into the discussion to clarify these intentions (lines 820-822). It reads:

"*While the quantitative results of our models should not be used directly to predict culture results for any host-pathogen system besides non-human primates and SARS-CoV-2, the general framework could be adapted easily to generate similar predictions for other host species, other viruses, or other assays."*

In the text that follows (lines 822-853), we also outline how our framework could be adapted and tested on other systems, including other human respiratory viruses.

Reviewer #2:

Thank you for your careful reading of our manuscript and for your attention to the experimental and statistical details, as well as our interpretation. We have carefully considered and addressed all of your suggestions (as outlined below), and we believe this has meaningfully enhanced the clarity and potential impact of our work.

#1:
Line 97
"Our results have many applications including: 1. to improve PCR-based clinical decisions with more accurate predictions of infectious status"

Unclear if study does this in a clinically significant way. For instance, would small gains in prediction accuracy percent seen in figure 2 (81% to ~85% for culture logistic) make a meaningful clinical difference (ie days to ending isolation). Does use of additional predictors decrease the error in predictions of days an animal is infectious such that it would have clear effect on duration of isolation. The goal of this paper seems to be an attempt to improve on current best practices by using a model to determine infectivity. If they can demonstrate this in a clinically meaningful way, it would strengthen the impact of this study.

Thank you for drawing attention to this point, and for this suggestion to enhance the clinical relevance of our study. As summarized above, we have made two types of revision in response. First, we have been much more careful to specify what are the immediate aims and

contributions of our work, and what are the possible future extensions (including adaptation to human data and direct clinical applicability). Second, in direct response to this comment, we have conducted new analyses that compare our simple and best model's ability to accurately identify when individual NHPs are no longer infectious, as an illustration of potential future decision support for human cases.

Under our test-based protocol, we explore the tradeoff in unnecessary days spent in isolation versus the risk of releasing individuals that are still infectious (measured in infectious days). We also benchmark these metrics against standard guidelines to isolate for five or ten days after the first positive test. To summarize, we show that our best model results in the smallest number of days spent isolating, with minimal impact on the number of days an individual may still be infectious. We also show that our best model is consistently more confident than our simple model in predicting the correct culture classification, which could be important in clinical settings where patient management decisions may require additional certainty. Finally, we demonstrate that the threshold probability at which a sample is predicted to be culture positive can be manipulated to find the optimal balance of isolation and infectious days, depending on context and local priorities. Please find the full details of this analysis in the new results section (*The best culture model shows potential for accurate, individualized isolation practices;* lines 584-642).

#2:
I have concerns about the comparability of data across studies because even "absolute copy number" is likely relative to the particular standard used in each study. Interestingly, most studies used invitro transcribed RNA for sgE standards however, some did not. (**see below for method used in studies). It is not clear if the same invitro transcribed and quantitated RNA was used across studies. If the authors could explore the data to evaluate whether lab or study influences variation seen, this may alleviate concerns about comparing quantities across studies.

Related to above concern:
Lines 302-303: Referring to Figure 3E. Is it possible that "considerable unexplained variation" noted is an artifact of comparisons across studies where copy numbers are determined using different standards. In figure 3E for the linear comparison (red data points) it almost appears as there are 2 clusters of data with an upper cluster of data points and a parallel lower cluster of points. Do these apparent clusters correlate with different studies or labs?

Line 571-572 "multiple factors enable us to rigorously analyze our aggregate database: (i) …RNA copy numbers, which are internally standardized as opposed to unstandardized CT values (68)."

Caution is warranted in interpreting copy number reported as absolute and comparable. It is unlikely that a universal RNA standard was used across studies. Differences in invitro transcription, RNA extraction, and efficiency of qPCR reactions (to name a few variable) across laboratories could account for differences seen. The authors allude to this issue in the discussion Line 591-593: "…it remains possible that lab or study effects impacted our results…"

**Methods of generating standards
Baum: In vitro transcribed RNA for sgE standard
Chandrashekar: In vitro transcribed RNA for sgE standard
Corbert: In vitro transcribed RNA for sgE standard
Patel: In vitro transcribed RNA for sgE Standard
Salguero: In vitro transcribed of RNA for SgE standard
Li and Edwards: In vitro transcribed E and N

Dagotto: In vitro transcribed sgE for standard

Gabitzsch
“A standard curve comprised of synthetic RNA containing the corresponding target sequence from SARS-CoV-2 isolate WA1 sequence” Bio-synthesis Inc.

This synthetic RNA should not contain sgRNA but only gRNA and should not amplify with sgRNA primers. I suspect they used the gRNA standard to quantitate sgRNA but they use N primers for gRNA

Sparanza: “In each run, standard dilutions of counted RNA standards were run in parallel to calculate copy numbers in the samples”. Unclear if this was invitro transcribed or copy number determined using an alternative such as digital PCR.

> Thank you for sharing this concern, and for highlighting some of the specific technical issues that could underlie lab or study effects. We have incorporated additional analyses that we believe will alleviate worries that our results are primarily driven by unmodelled differences in the methods of disparate lab groups. In summary, we ran our best sgRNA model and also our best culture model with an additional categorical predictor for lab effects, where we grouped articles based on where they conducted their primate study (Table S8). Notably, all but two articles that you identified used in vitro transcribed RNA for sgE standard were grouped together into one lab category under this method (the only exceptions are Li and Salguero).
>
> Our qualitative findings did not change between the models with and without a lab effect (Figures S12, S16), with only one exception. This exception was for the linear component of the sgRNA model, where the dose effect weakened and reversed in the model with lab effect. We describe this in the associated results section (lines 393-395), and we offer our thoughts on what mechanism could drive this pattern in the discussion (though we note future experimental work is needed to thoroughly investigate; lines 701-706). We have added text throughout the manuscript to further describe and discuss all of these lab-effect analyses.

Reviewer #3: None.

Part III – Minor Issues: Editorial and Data Presentation Modifications
Please use this section for editorial suggestions as well as relatively minor modifications of existing data that would enhance clarity.

Reviewer #1: See below.

Please see our responses to this reviewer's concerns in Part II.

Reviewer #2: Line 30: Not sure clinical significance of infectivity of non-inoculated tissues. Other tissues (brain, spleen, intestines) lively have much less influence, if any, on infectivity compared to respiratory tract where virus is shed. Consider removing this example from author summary as non-inoculated samples are not sampled in clinical setting.

We appreciate your suggestion, but this example is intended to inform animal challenge experiments, where non-inoculated tissues are regularly sampled to understand pathology and where interpretations of early infectivity in inoculated tissues must consider the effect of residual inoculum. We have added text to clarify the relevance of these results. This sentence now reads (lines 31-34):

"For example, we show that inoculated tissues are more likely to be culture-positive (for a given PCR result) on the first day post infection than all later days post infection or non-inoculated tissue on any day – a finding that will clarify interpretation of results in experimental studies."

We have, however, included an additional sentence that mentions the potential clinical application of our model. That sentence reads (lines 34-36):

"Beyond these biological findings, we also showed that our framework can accurately identify when an individual is no longer infectious, showing the potential for future versions (trained on human data) to offer an individualized approach to ending isolation."

Line 32/33. Unclear what "quantitative predictions of infectiousness" means. Does this refer to viral load at which animals are no longer infectious? Please clarify.

We have rephrased this sentence to clarify that we mean our model can predict viral culture outcomes, which is our best available proxy for an individual's infectiousness. This sentence is now on lines 36-38, and it reads:

"*Overall, our work presents a standardized framework to quantitatively predict viral culture outcomes based on faster and cheaper assays, which can be readily adapted to any other pathogen-host system with relevant data."*

Line 55: sgRNA has not been widely used on clinical diagnostics (I am not aware of its use ever in clinical diagnostics in humans). Please clarify in text.

We were able to find a report in the literature where pediatric clinicians used sgRNA results to inform therapeutic management and to determine whether surgical procedures could proceed. The reference is:

Osborn et al. Clinical utility of SARS-CoV-2 subgenomic RT-PCR in a pediatric quaternary care setting. *J Clin Virol*. 2023 Jul;164:105494. doi: 10.1016/j.jcv.2023.105494

We have altered our language and added another sentence to emphasize that, while retrospective analysis of clinical samples with sgRNA assays are common, sgRNA use for clinical diagnostics is very rare. This passage now reads (lines 56-62):

"Since sgRNAs are only transcribed after cellular entry and are generally not packaged into mature virions (5), sgRNA-specific assays for SARS-CoV-2 were developed as a proxy for replicating virus (6), and they have been used in various contexts, including to distinguish between replicating virus and residual inoculum in animal challenge experiments (7,8). Many studies have also retrospectively analyzed clinical samples with sgRNA assays to gauge evidence of local replication (6,9–12), but reports of using sgRNA for point-of-care clinical decisions are exceptionally rare (13)."

Line 58: Degradation rate of subgenomic RNA not established in paper cited (Speranza et al.) Limited time points in that study make estimates of differential degradation very difficult. Speranza results between days 1-3 could be explained by lower total expression levels of sgRNA compared to total RNA. The paper also does not provide a calculated half-life for each RNA species.

Our rationale for this statement is based on the individuals administered with irradiated inoculum, in which both gRNA and sgRNA were detectable at high quantities before the inoculation procedure (indicated by points on the y axis of Figure 1A of Speranza et al.).The assay-specific limits of detection are not available, but they can be approximated by the smallest reported quantity for each assay (~2 log10 for gRNA, and ~3 log10 for sgRNA). Based on these estimated LODs and the starting quantities of each RNA type, gRNA reduced by around 2 log10 copies for AGM1 and about 4 log10 copies for AGM2 within the first day, whereas sgRNA reduced by *at least* 3 log10 copies for both individuals (and possibly more, depending on LOD) in the same time frame. Given the authors who know these additional details have also interpreted their results as rapid sgRNA decay, we believe this study provides supporting evidence although not definitive proof that these RNA species may have differential degradation rates. We have updated the language of our sentence to reflect this uncertainty, and it now reads (lines 64-66):

"Based on evidence that sgRNA may degrade faster than gRNA(8), is not found in virions (5), and correlates better with viral culture results (9–12), some consider sgRNA a better indicator of recent replication and infectiousness (6,13)."

Line 84. "…thus, no best practices exist to predict and individuals infectiousness."

There are "best practices" outlined by CDC and are generally based on duration of infectivity from cell culture studies. The authors should consider discussing these or remove the sentence from intro. There are also test-based "best practices" to end isolation with antigen testing. I think the goal of this study is to improve on current "best practices". The authors should demonstrate this if they can.

As the reviewer suggested, we have summarized the CDC guidelines for isolation, and we have noted that an individualized approach could improve these practices. This passage now reads (lines 88-99):

“Despite considerable effort to correlate RNA presence with culture outcomes, no study yet has jointly evaluated these various cofactors to identify and quantify their effects, and thus no method exists to integrate all of this information to quantitatively predict an individual’s infectiousness on a per-sample basis. Instead, public health agencies have recommended isolating until obtaining two consecutive negative tests or until ten days after an individual’s first positive test, where the latter was later revised to only five days depending on symptom severity and other risk factors (31). However, some individuals experience prolonged shedding, and many individuals cease to be infectious well before testing PCR or antigen negative (6,14,19,32). An individualized, evidence-based method to ending isolation (i.e., a precision medicine approach) could improve these practices substantially, by alleviating personal and economic burdens imposed by unnecessarily long isolation while also reducing the number of days individuals may still be infectious after release under static guidelines.”

Line 256/257: Is “simple model” used here a simple linear regression? Please clarify.

We have edited this sentence to clarify, and it now reads (lines 308-310):

“To benchmark our analysis against prior work, we also evaluated the ‘simple model,’ for which the logistic and linear components contain PCR results as the sole predictor (i.e., it is a hurdle model comprised of a simple logistic regression and a simple linear regression).”

Line 270: Figure 2. Culture logistic. It appears visually that best model is T+DPI+AGE+TG+Aassy+SP+Dose, which seems to have a higher prediction accuracy and MCC than T+DPI+AGE+TG+Aassy+SP+Dose+Cell. Please clarify which is best model.

To choose the best model, we required it to have similar or better performance as the full model on prediction accuracy, MCC, and ELPD. The model without Cell was not chosen because, although it outperformed the full model on MCC and prediction accuracy, it underperformed on ELPD. This is because the ELPD for the full model was larger than the ELPD for this model by more than our threshold of 4 units. These criteria are outlined in the methods, and a full component-by-component description of our model selection is included in the Supplementary Methods. We have added a sentence in our figure legend to briefly explain this result, and we have also explicitly referenced the Methods and Supplementary Methods. These additions read as follows (lines 328-332):

“For the culture logistic component, the model with seven predictors was not chosen because, although it outperformed the full model on MCC and prediction accuracy, it underperformed on ELPD. This is because the ELPD for the full model was larger than the ELPD for this model by more than our threshold of 4 units. Please see the Methods for more details about our selection criteria and the Supplementary Methods for a full description of the selection procedure.”

Line 292: Any conclusions about the finding that AGM having “highest chance of sgRNA detection” should be made with caution. There is only one study that included AGM and sgE RNA levels (Sparanza et al). Sparanza et al. don’t explicitly indicate how they generated standard curves. Most other studies of sgE used invitro transcribed RNA. The differences observed could be technical not biological. Finally, it appears from figure 3C that AGM have a lower chance of detection of sgRNA

(31% AGM vs 65% CM and 66% RM) not a higher chance (this may be stated correctly in discussion starting line 525).

Thank you for noticing our typo. We have now correctly stated in line 349 that African green monkeys have the *smallest* chance of sgRNA detection.

Our results for the differences between these species were consistent between the models with and without a lab effect, which we have shown in Figure S12. However, we have reduced the emphasis on our species results throughout the manuscript in response to your broader concerns about data limitations surrounding species effects.

Line 326-329: Again, caution when making conclusions about species and sgRNA expression Comparisons are based on copy numbers obtained from standards curves generated in different labs. If this is a biologic finding, it would suggest something interesting about subgenomic RNA expression in different species.

Cynomolgus macaques were predicted to have the smallest sgRNA quantities regardless of whether a lab effect was included in the model or not. However, the difference was much smaller when lab effects were included. We have described these results while also emphasizing that further work is necessary to confirm whether the patterns reflect true biological differences. This is now in lines 401-406 and reads:

"The best model also predicted that sgRNA quantities vary by species. Regardless of whether a lab effect was included, rhesus macaques and African green monkeys had highly similar predictions. Cynomolgus macaques were predicted to have lower median sgRNA quantities for any given totRNA quantity, though this effect was substantially reduced when lab effects were included. Given that only one lab group had data from both cynomolgus macaques and another species (rhesus macaques), we view this species effect as an intriguing but tentative finding that warrants further investigation."

Line 339-340: Not clear how grey boxes are determined. How is a difference determined to generate these boxes? For instance, in figure 3C, it appears that there are differences in chance of positivity across cofactors that are not within the grey boxes.

We have added an example to the figure legend to help clarify how these boxes were determined. It reads (lines 415-420):

"For the simple model, the grey box encloses totRNA copies where classifications differ among the simple model and any possible combination of cofactors in the best model, based on our standard prediction threshold of 50%. For all other rows, grey boxes enclose regions where classifications differ within the displayed cofactor group for the standardized cofactor set. For example, 5 log10 totRNA copies / sample is enclosed for 'Species' because African green monkeys are predicted to be negative while both other species are predicted to be positive. The rows for the other cofactor groups (e.g., target gene) do not influence the grey boxes for 'Species'."

Line 487/488. "Our models identify key sources of biological variation (including exposure conditions, demographics and assay protocols)." Not clear how assay protocols represent biological variation. Please clarify.

We have edited this sentence to clarify that our model identifies sources of both biological and methodological variation (lines 677).

Line 488-489: "We showed that because standard, simple regression models ignore this variation…" -This is the first mention of "simple regression models" make clear that the "simple model" referred to in this study is a "simple linear regression" if this is the case.

In response to this comment and the one on lines 256-257, we have clarified that our 'simple models' are single regression models with only one predictor variable. We made these changes on lines 308-310 and 679.

Line 521: Not sure you can distinguish between active replication and "high stability" of RNA in vivo. The manuscript cited (63) evaluated ex-vivo stability in mucus samples so no replication occurs. Clarify or removed comment about RNA stability.

We have clarified that this study was conducted *ex-vivo*. The sentence now reads (lines 715-718):

"*These patterns are consistent with most inoculated virions having infected cells, dispersed to other tissues, or been cleared by the immune system within the first two days of infection, whereas the high stability of RNA (at least in human respiratory fluids monitored ex vivo (63)) could enable its prolonged detection.*"

Line 522: Discussion makes clear that species differences are limited to small samples sizes. It seems that this finding is over emphasized throughout manuscript.

As stated above, we have altered our language and added sentences in our results sections to communicate an appropriate degree of uncertainty surrounding the species results.

Line 537: Does this discussion imply that S gene would be a better predictor of infectivity? Because of variation found in Spike, primer and probe binding and PCP efficiency could be affected using this as a target in clinical studies. It would be difficult to compare copy number across variants and it would not be great for quantitative diagnostics.

We did not intend to suggest that the S gene is a better predictor of infectivity – all genes can function equally well for generating predictions, they just require different quantities of RNA to expect the same chance of culture positivity. We have clarified this in lines 741-744, which read:

"Notably, this result does not imply that spike assays better predict infectivity. Different genes simply require different RNA quantities to expect the same chance of culture positivity, and so other considerations should motivate choice of target gene (e.g., selecting target sequences that are conserved across variants)."

Line 554: In this part of the discussion, the authors identify prediction errors at "transition periods". It seems that this is a critical finding as prediction of infectivity must occur in these transition periods between culture positivity and negativity. It would be interesting to know how the best model compares to the simple model in transition periods (but not necessary).

The simple model had a large increase in the number of edge errors relative to the best model. The simple model also had a few more transition errors, and many of the edge errors appeared during what could be a transition between positive and negative states (i.e., they may also be transition errors). We have added text in this results section that compare error types between the simple and best models (throughout lines 553-559). It reads as follows:

"When considering all prediction errors, we find that edge errors are the most common for both the best (n=51/131; 38.9%) and simple (n=78/171; 45.6%) models. Transition errors, however, are of particular interest, given that the shift from positive to negative states determines the end of infectivity. The best model made 44 transition errors (n=44/131; 33.6%), while the simple model made 49 transition errors (n=49/171; 28.7%). We then calculated how many edge errors could also be considered transition errors, and once again we found that the best model made fewer such errors (23 vs. 34). Thus, model accuracy appears to improve at this critical point during infection when accounting for key covariates."

We also further compared our simple and best model's prediction accuracy on all samples with intermediate totRNA quantities (6-8 log10 copies), as they often occur during the critical transition between culture positive and negative states. We found that the best model's improvement over the simple model is especially pronounced in this range, with the best model correctly identifying 23.3% more culture positive samples. These results are discussed on lines 467-471, with Figure S15 as the supporting figure.

Reviewer #3:

Here below I list a few minor suggestions to strengthen the paper:
1. In the abstract, the authors claim that 'our model framework can be adapted to compare any assays, in any host species, and for any virus'. While I agree that the framework is a useful tool that can be adapted to make valuable predictions in other systems (e.g. a novel virus that has potential to cause the next pandemic), I think this statement is too strong for two reasons. First, it is very likely that other viruses and the corresponding PCR developed may have their own characteristics (that were not considered in this work). In this case, careful model development is still needed. Second, the authors did not show the applicability of this model to other systems, which makes the statement speculative.

This is an excellent point, which we tried to convey with the original phrasing, but which evidently needed to be clearer. We have altered this sentence in our abstract to express that model development is still required in other scenarios and that those models could (but won't necessarily) be useful. The new sentence is (lines 20-22):

"Our analytical pipeline offers a framework to develop similar predictive tools in other virus-host systems, including models trained on human data, which could support laboratory analyses, medical decisions, and public health guidelines."

We have also added text in the discussion section to explicitly state that rigorous model development in new scenarios is still required. This reads (lines 825-828):

"Notably, when applying the framework to other scenarios, careful model development is still necessary, especially given that different viruses and assays may have other defining

characteristics that could affect their relationships, which should influence the choice of candidate cofactors."

2. I think it'd be useful to have a table listing the best fit values (and the credible intervals) for the best models. This would be useful for readers to better use the model to make their own predictions from newly generated data.

Thank you for this suggestion. We have now included an additional table in the supplementary material (Table S7) that includes the median estimated parameters, their 90% credible intervals, and their standard deviations. We have also added sentences in our results sections (e.g., lines 483-484) to bring this table to the reader's attention.

3. Since the ultimate goal of the work is to use/adapt the model to human data, I think it'd strengthen the paper if the authors discuss the results of the work in the context of other efforts trying to relate SARS-CoV-2 load to infectiousness in humans. For example:
Ref. 77 in the manuscript, Ke et al. PNAS 118 (49), e2111477118, and Aurélien, et al. Elife 10 (2021): e69302.

Thank you for this suggestion. We have added a paragraph in our discussion that relates our results to patterns observed in studies conducted on humans, highlighting many points of concordance. For example, our simple model predicts that samples with around 7 log10 totRNA copies have approximately 50% chance of being culture positive, which falls within the range estimated by multiple other studies conducted using human data (5-9 log10 RNA copies). Other work has also found evidence that age influences culture results. We have summarized these (and other) similarities in our new paragraph (lines 755-770).
